# hnRNPC facilitates coronavirus replication by directly binding the frameshift-stimulatory element of viral genomic RNA

**DOI:** 10.1371/journal.ppat.1014491

**Published:** 2026-08-07

**Authors:** Jingchen Xu, Hongying Li, Jianrui Li, Biao Dong, Hu Li, Han Sun, Jiayu Zhang, Jiujiao Gao, Yue Gong, Xinyue Liu, Zidie Nian, Tong Wang, Ning Li, Jiandong Jiang, Zonggen Peng

**Affiliations:** 1 CAMS Key Laboratory of Antiviral Drug Research, Institute of Medicinal Biotechnology, Chinese Academy of Medical Sciences & Peking Union Medical College, Beijing, China; 2 Beijing Key Laboratory of Technology and Application for Anti-Infective New Drugs Research and Development, Institute of Medicinal Biotechnology, Chinese Academy of Medical Sciences & Peking Union Medical College, Beijing, China; 3 State Key Laboratory of Bioactive Substance and Function of Natural Medicines, Institute of Medicinal Biotechnology, Chinese Academy of Medical Sciences & Peking Union Medical College, Beijing, China; Stanford University, UNITED STATES OF AMERICA

## Abstract

Translation of key viral replicative proteins in coronaviruses requires a programmed –1 ribosomal frameshifting (–1 PRF) event controlled by the viral frameshift-stimulatory element (FSE). Although previous studies have analyzed host factor dependencies of coronaviruses, how host cellular factors alter –1 PRF efficiency and affect viral replication remains poorly understood. Here, using RNA pull-down combined with LC-MS/MS analysis, we identified heterogeneous nuclear ribonucleoprotein C (hnRNPC) as a major interacting protein of FSE RNA. Coronavirus infection triggers hnRNPC mRNA decay, alters hnRNPC protein levels, and induces its cytoplasmic relocalization, where it appears to bind directly to FSE RNA through residues Asn7 and Asn83. This binding is associated with increased –1 PRF efficiency and may facilitate coronavirus replication. Deletion mapping analysis shows that hnRNPC preferentially binds U-rich regions of the FSE RNA. Finally, we demonstrated that the small molecule Elbasvir directly binds hnRNPC, disrupting the interaction between hnRNPC and FSE RNA and inhibiting coronavirus replication by decreasing –1 PRF efficiency. Collectively, our study identifies hnRNPC as a key host cofactor for coronaviruses and provides a novel target for broad-spectrum antiviral drug development.

## Author summary

Coronaviruses rely on a programmed -1 ribosomal frameshifting (–1 PRF) event to produce the viral proteins required for replication. This process is controlled by the frameshift-stimulatory element (FSE) within the viral genome, but the role of host cell proteins in –1 PRF remains under investigation. Here, we identified the host protein heterogeneous nuclear ribonucleoprotein C (hnRNPC) as a key regulator of this process through its direct interaction with the FSE. Coronaviruses alter the levels and cellular location of hnRNPC, allowing it to relocalize to the cytoplasm, bind viral RNA, and increase viral protein production. As a result, viral replication is enhanced. We also showed that this interaction depends on specific regions within the viral RNA. Importantly, we found that the small molecule Elbasvir can disrupt the interaction between hnRNPC and the FSE, thereby inhibiting coronavirus replication. Together, our findings provide new insight into how coronaviruses exploit host cell machinery and identify a host-directed target that may be useful for developing broad-spectrum antiviral therapies.

## Introduction

Coronaviruses (CoVs) are enveloped, single-stranded, positive-sense RNA viruses classified into four genera, *Alphacoronavirus*, *Betacoronavirus*, *Gammacoronavirus*, and *Deltacoronavirus*, which primarily infect birds and mammals. The seven coronaviruses known to infect humans belong to the genera *Alphacoronavirus* and *Betacoronavirus*. In humans, the circulating coronaviruses HCoV-229E and HCoV-NL63 are *Alphacoronaviruses*, while HCoV-HKU1, SARS-CoV, MERS-CoV, HCoV-OC43, and SARS-CoV-2 are *Betacoronaviruses* [1]. HCoV-229E, HCoV-NL63, HCoV-OC43, and HCoV-HKU1 generally cause mild upper respiratory illness in humans, while SARS-CoV, MERS-CoV, and SARS-CoV-2 can cause severe respiratory illness. Although the general genome organization and replication cycle are conserved across all human coronaviruses, they differ in receptor usage, internalization mechanisms, and the number of accessory genes produced [2]. The coronavirus disease 2019 (COVID-19) pandemic, caused by SARS-CoV-2, has become an unprecedented global public health crisis, posing a substantial threat to health security worldwide [3]. SARS-CoV-2 causes extensive damage to various organ systems through the host immune response. Several studies have investigated host-virus interactions [4–8] and viral pathogenesis [2] to develop prevention and treatment strategies. However, COVID-19 remains a relatively new disease, and its full pathogenesis has not yet been fully elucidated, particularly because the key cellular and molecular mechanisms underlying viral replication are not completely understood. Moreover, effective and specific antiviral therapies targeting SARS-CoV-2 remain limited. Therefore, identifying potential therapeutic targets for future treatment and prevention of SARS-CoV-2 infection is an urgent and critical need.

A key feature of SARS-CoV-2 and many other positive-strand RNA viruses is the occurrence of programmed -1 ribosomal frameshifting (–1 PRF), which enables the translation of multiple proteins from a single mRNA transcript. In coronaviruses, –1 PRF at the pp1a/1b junction is critical for efficient viral replication and translation of the viral RNA genome. The –1 PRF process is driven by a structured RNA element at the 3’ end of open reading frame (ORF) 1a, known as the frameshift stimulation element (FSE). This element has a typical RNA pseudoknot structure consisting of a heptameric slippery sequence (UUUAAAC), a short spacer, and a 3-stem-loop architecture [9]. The 3-stemmed pseudoknot structure interacts with the ribosome at the entrance to the mRNA channel, blocking the backward slippage of the ribosome [10,11]. When frameshifting does not occur, the viral RNA genome is translated to produce the ORF1a-encoded polyprotein pp1a. When –1 PRF occurs, the ribosome shifts backward by one nucleotide, enabling the translation of ORF1b. The full-length polyprotein pp1ab, translated from ORF1ab, encodes the complete set of nsp1–nsp10 and nsp12–nsp16, all of which are essential for viral replication.

Multiple host factors have been identified that modulate viral –1 PRF efficiency. Among these, some exert antiviral effects by targeting the –1 PRF process. For example, Shiftless has been shown to broadly inhibit –1 PRF across a variety of viruses [12–16], while the short isoform of the zinc-finger antiviral protein (ZAP-S) directly binds SARS-CoV-2 FSE RNA and interferes with the proper folding of the FSE, thereby disrupting frameshifting [17]. In addition, certain host proteins have been shown to promote –1 PRF in specific viral contexts. Notably, nsp1β and cellular poly(C)-binding proteins form a complex that acts in trans to activate –1 PRF in porcine reproductive and respiratory syndrome virus [18]. Recent studies have further shown that eukaryotic initiation factor 2A (eIF2A) and stem loop binding protein (SLBP) specifically enhance –1 PRF in SARS-CoV-2 [19,20]. Therefore, host factors represent important regulators of –1 PRF and may serve as potential targets for antiviral intervention.

To identify potential therapeutic targets for disrupting coronavirus –1 PRF, we systematically determined the direct protein interactors of the SARS-CoV-2 frameshift RNA element using RNA pull-down combined with LC-MS/MS. Among more than 300 proteins identified, we found that the RNA-binding protein heterogeneous nuclear ribonucleoprotein C (hnRNPC) was the most prominent interaction partner. hnRNPC, a member of the heterogeneous nuclear ribonucleoprotein (hnRNP) family with over 20 members, consists of three C1 subunits and one C2 subunit. The C2 isoform contains 306 amino acids, while the C1 isoform lacks 13 amino acids in the 108–120 region relative to C2. It primarily localizes to the host cell nucleus and plays key roles in RNA processing, including mRNA splicing, retention and packaging, maintenance of mRNA stability and export. Additionally, hnRNPC has been implicated in translation initiation through its binding to internal ribosomal entry sites on mRNA [21,22]. Here, we demonstrated that hnRNPC acts as a cofactor that promotes coronavirus –1 PRF. Using a multidisciplinary approach, we further investigated the key molecular mechanism by which hnRNPC promotes –1 PRF, revealing the specific binding mode between hnRNPC and FSE RNA. Targeting hnRNPC with Elbasvir also inhibited HCoV-OC43 and HCoV-229E infection *in vitro* by interrupting the interaction between hnRNPC and FSE RNA. Together, these findings identify hnRNPC as a host cofactor of coronaviruses and a therapeutic target against coronavirus variants.

## Results

### SARS-CoV-2 FSE RNA capture identifies novel host interactors

**Fig 1:**
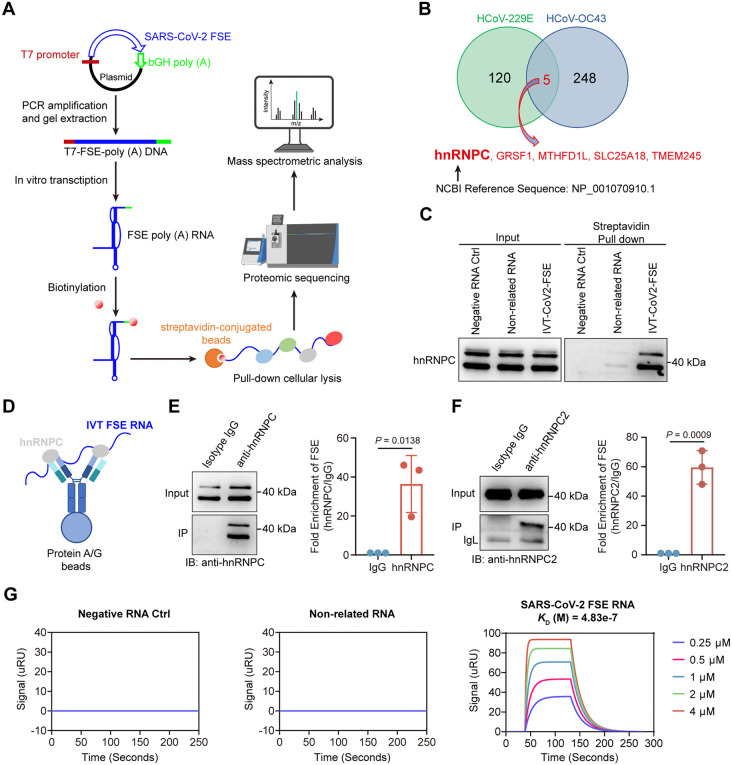
Discovery of protein interactors of the SARS-CoV-2 FSE RNA. **(A)** Schematic of *in vitro* interactome capture from protein interactors of biotinylated IVT SARS-CoV-2 FSE RNA. **(B)** Host proteins identified from HCoV-OC43-infected H460 cells and HCoV-229E-infected Huh7 cells. **(C)** RNA pull-down assays. hnRNPC proteins were detected by immunoblotting in Huh7 cell lysates. **(D-F)** Graphic illustration of RNA immunoprecipitation (RIP) experiments (**D**) using anti-hnRNPC (**E**) or anti-hnRNPC2 (**F**) antibody to capture IVT FSE RNA. Representative immunoblot showing hnRNPC protein in input and immunoprecipitation (IP) samples for RIP assay (left panel). SARS-CoV-2 FSE RNA was quantified by qRT-PCR after enrichment from RIP (right panel). **(G)** SPR analysis of affinity binding between hnRNPC and RNA. Data were shown as mean  ±  s.d. with individual data points. Statistical analyses were performed using an unpaired, two-tailed Student’s *t-test*.

To identify potential cellular RNA-binding proteins (RBPs) that interact with the FSE RNA of SARS-CoV-2, biotinylated *in vitro* transcription (IVT) FSE RNA from the SARS-CoV-2 genome was incubated with lysates of H460 cells infected with HCoV-OC43 and Huh7 cells infected with HCoV-229E. To exclude non-specific binders, a non-related RNA was used as a control. RNAs were captured using streptavidin magnetic beads, and interacting proteins were identified by liquid chromatography-tandem mass spectrometry (LC-MS/MS) analysis (Fig 1A).

We identified more than 300 proteins that bind to FSE RNA but are not enriched in the non-related RNA. GO and KEGG enrichment analyses of these proteins revealed that most are highly associated with ribosomes and function as RNA-binding proteins (S1 Fig). Among these, five proteins were identified in both HCoV-OC43-infected H460 cells and HCoV-229E-infected Huh7 cells, including hnRNPC, G-rich RNA sequence binding factor 1 (GRSF1), methylenetetrahydrofolate dehydrogenase (NADP+ dependent) 1 like (MTHFD1L), solute carrier family 25 member 18 (SLC25A18) and transmembrane protein 245 (TMEM245) (Fig 1B and S1 Table). Several of these interactors have been shown to play roles in RNA processing, including splicing (hnRNPC) [23] and RNA surveillance (GRSF1) [24]. MTHFD1L is involved in the synthesis of tetrahydrofolate in the mitochondrion and provides activated methyl groups needed for nucleotide biosynthesis and viral replication [25]. SLC25A18 is a transporter of glutamate and H^+^ across the mitochondrial inner membrane [26], while TMEM245 is associated with tumor development [27,28]; the roles of both in viral infections have not been reported. hnRNPC has been reported to be involved in the infection process of various viruses [29–31], but its interaction mechanism with coronavirus FSE RNA has not been studied. Therefore, we selected this host factor for investigation.

To confirm the LC-MS/MS results, we used immunoblotting to examine the binding of hnRNPC to biotinylated IVT CoV-2 RNA. Negative control RNAs did not bind to hnRNPC, while IVT CoV2 RNAs bound more hnRNPC than non-related RNAs (Fig 1C). We further evaluated the interaction between hnRNPC and the FSE RNAs of HCoV-OC43 and HCoV-229E, given the conservation of FSE RNA sequences among coronaviruses (S2 Fig). We observed similar results with HCoV-229E and HCoV-OC43 FSE RNA pull-downs (S3A Fig). Next, we transfected IVT CoV-2 RNAs into Huh7 cells and performed RNA immunoprecipitation (RIP) experiments by immunoprecipitating hnRNPC from cell lysates, followed by detection of hnRNPC-bound RNAs (Fig 1D). When incubated with equal amounts of anti-hnRNPC antibody or isotype-matched IgG antibody (Fig 1E, left panel immunoblot), IVT CoV-2 FSE RNAs bound to hnRNPC compared to the IgG control (Fig 1E, right panel). Transfected IVT HCoV-OC43 and HCoV-229E FSE RNAs were also enriched in Huh7 cells (S3B and S3C Fig). Additionally, we performed hnRNPC2 RIP using anti-hnRNPC2 antibody or isotype-matched IgG to determine whether the longest isoform of hnRNPC could bind IVT CoV-2 FSE RNAs. We found that IVT CoV-2 FSE RNA binds to hnRNPC2 (Fig 1F), consistent with the hnRNPC results. We also used surface plasmon resonance (SPR) spectroscopy to test whether hnRNPC interacts directly with coronaviral FSE RNA. hnRNPC bound to the SARS-CoV-2 FSE RNA but not to the negative RNA control or non-related FSE RNA (Fig 1G). Similar results were obtained with HCoV-OC43 FSE RNA (S3D Fig) and HCoV-229E FSE RNA (S3E Fig). Altogether, these data show that hnRNPC binds to coronaviral FSE RNAs.

### Asn7 and Asn83 of hnRNPC are required for the specific binding of FSE RNA

**Fig 2:**
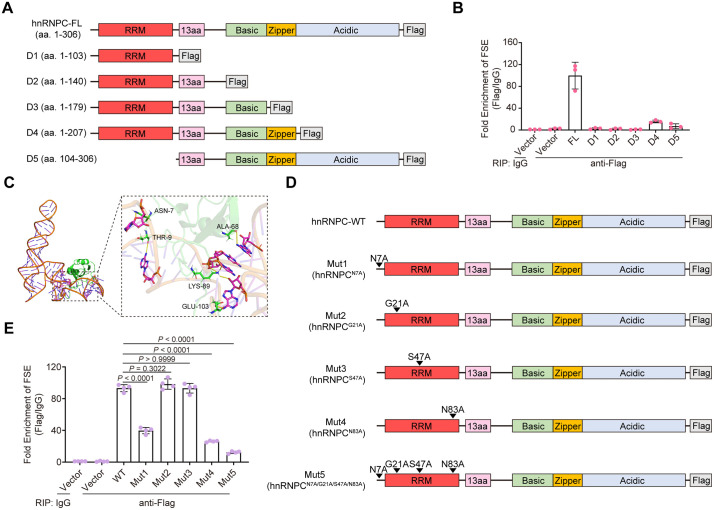
SARS-CoV-2 FSE RNA binds to ASN7 and ASN83 of hnRNPC. **(A)** Domain mapping of Flag-tagged full-length (FL) and truncated hnRNPC. **(B)** RNA binding activity of FL and truncated hnRNPC with SARS-CoV-2 FSE RNA in Huh7 cells. **(C)** Binding mode between SARS-CoV-2 FSE RNA and hnRNPC predicted by molecular docking. **(D)** Schematic representation of wild-type and mutated hnRNPC proteins. **(E)** RNA binding activity of WT and mutated hnRNPC with SARS-CoV-2 FSE RNA in Huh7 cells. Data were shown as mean  ±  s.d. with individual data points. Statistical analyses were performed using one-way ANOVA followed by Dunnett’s post hoc comparison test.

To investigate the structural domains of hnRNPC involved in the interaction with FSE RNA, we first constructed five Flag-tagged truncated hnRNPC fragments based on its structural domains (Figs 2A and S4). RIP assays using an anti-Flag antibody showed that all hnRNPC deletion mutants lost RNA-binding activity (Fig 2B), suggesting that the full structural integrity of hnRNPC is required for RNA binding. Molecular dynamics simulation further revealed the binding mode of hnRNPC with SARS-CoV-2 FSE RNA. The results showed that five residues of hnRNPC (ASN7, THR9, ALA68, LYS89, and GLU103) formed interactions with FSE RNA (Fig 2C). The predicted binding free energy value (∆G) of the complex was -22.7 kcal/mol, indicating a stable interaction between the two molecules. Based on the molecular docking model and previously reported crystal structures of the hnRNPC RRM in complex with 5’-AUUUUUC-3’ RNA (PDB ID: 2MXY) [32], we generated a series of hnRNPC mutants (Fig 2D) and found that mutation of Asn7 or Asn83, either alone or together, disrupted the interaction between FSE RNA and hnRNPC, as indicated by RIP assays (Figs 2E and S5). These data indicate that Asn7, Asn83, and the structural integrity of hnRNPC are crucial for its binding to FSE RNA, supporting a specific and structurally dependent binding mode between hnRNPC and FSE RNA.

### hnRNPC binds to U-rich sequences of SARS-CoV-2 FSE RNA

**Fig 3:**
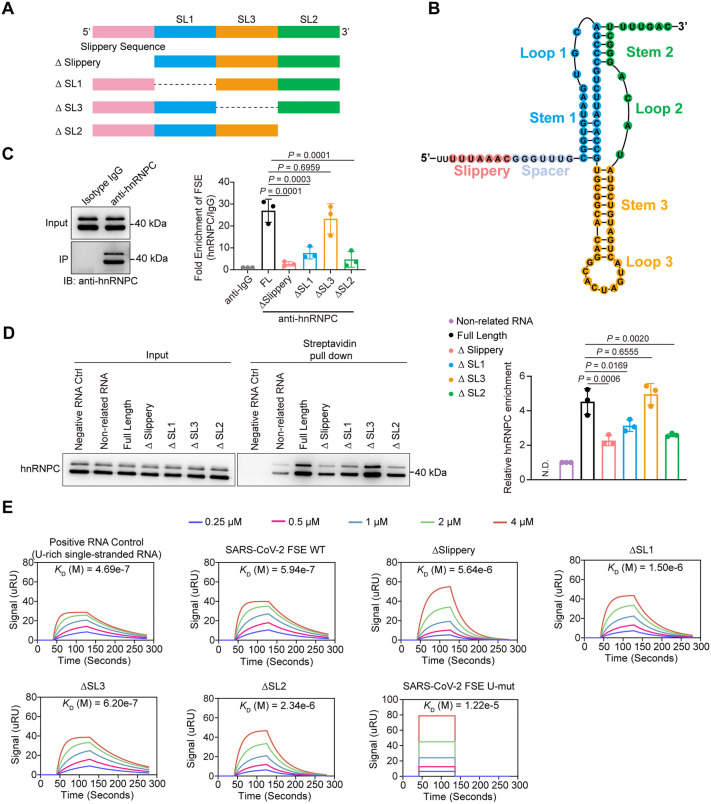
hnRNPC binds to U-rich sequences of SARS-CoV-2 FSE RNA. (**A**) Schematic view of FL and deleted fragments of SARS-CoV-2 FSE RNA. (**B**) Two-dimensional representations of the SARS-CoV-2 FSE RNA pseudoknot. (**C**) Representative immunoblot examining hnRNPC protein in input and immunoprecipitation (IP) samples for RNA immunoprecipitation (RIP) assay (left panel). FL and deleted SARS-CoV-2 FSE RNA quantified by qRT-PCR after enrichment from RIP (right panel). (**D**) RNA pull-down assay showing the binding activity of hnRNPC with FL or deleted SARS-CoV-2 FSE RNA. (**E**) SPR assays monitoring hnRNPC binding to RNA. U-rich single-stranded RNA served as the positive RNA control. For the RNA sequences used in the SPR assays, see also S4 Table. Data were shown as mean  ±  s.d. with individual data points. Statistical analyses were performed using one-way ANOVA followed by Dunnett’s post hoc comparison test.

We next constructed four deleted FSE RNA fragments (Fig 3A) based on the elements of the full-length FSE RNA pseudoknot (Fig 3B) to investigate the RNA structural elements that contribute to this interaction. Anti-hnRNPC RIP combined with qPCR experiments showed that the slippery site is essential for the interaction between hnRNPC and FSE RNA (Fig 3C). In addition, deletion of both SL1 and SL2 reduced the binding affinity between hnRNPC and FSE RNA (Fig 3C). Notably, as previously reported, the slippery site, SL1, and SL2 regions contain uninterrupted uridine (U) tracts (U-rich), consistent with the established binding preference of hnRNPC [32,33]. To confirm these results, we used a biotinylated RNA pull-down assay to examine the binding of hnRNPC to biotinylated IVT FSE RNA deleted mutants. Similarly, we found that deletion of the SL3 region did not affect hnRNPC enrichment, while deletion of the slippery site, SL1 and SL2 regions all decreased hnRNPC enrichment (Fig 3D). SPR analysis further confirmed that hnRNPC directly interacts with the slippery site, SL1, and SL2 regions, but not with SL3 (Fig 3E). Importantly, the binding affinity of hnRNPC to wild-type FSE RNA was comparable to that of a U-rich positive RNA control, whereas mutation of the U-rich tract within the FSE markedly decreased binding affinity, further supporting that uridine-rich sequences are critical determinants for hnRNPC recognition (Fig 3E). Altogether, our results demonstrate that the slippery site, SL1, and SL2 regions of FSE RNA, which contain U-rich sequences, are essential for mediating hnRNPC binding. The results further suggest the specificity of the binding between hnRNPC and FSE RNA.

### Coronavirus facilitates hnRNPC mRNA decay

**Fig 4:**
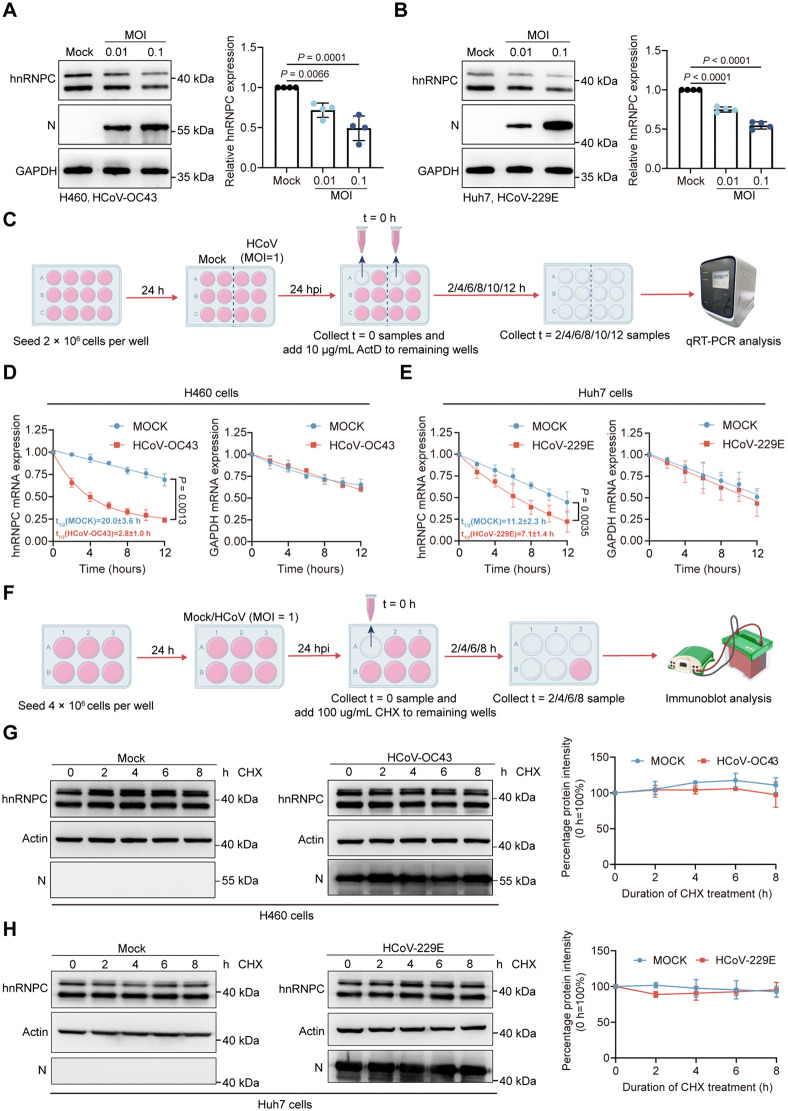
Effect of coronavirus infection on hnRNPC stability. **(A-B)** Protein levels measured by immunoblotting in HCoV-OC43-infected H460 cells (**A**) and HCoV-229E-infected Huh7 cells (**B**) at 48 hours post infection. **(C)** Schematic of the hnRNPC mRNA stability assay. (**D-E**) mRNA stability in HCoV-OC43-infected H460 cells (**D**) and HCoV-229E-infected Huh7 cells **(E)**. MOI = 1. **(F)** Schematic of the protein stability assay. **(G-H)** Protein levels measured by immunoblotting in HCoV-OC43-infected H460 cells (**G**) and HCoV-229E-infected Huh7 cells (**H**) treated with cycloheximide (CHX). MOI = 1. Data were shown as mean  ±  s.d. with individual data points. Statistical analyses were performed using unpaired, two-tailed Student’s *t-test* **(A-B)**, or paired, two-tailed Student’s *t-test* (**D, E, G, H**).

To further clarify the role of the interaction between hnRNPC and coronavirus, we measured hnRNPC expression levels in mock-infected and coronavirus-infected cells. We observed that HCoV-OC43 and HCoV-229E infection decreased hnRNPC protein abundance in H460 and Huh7 cells (Fig 4A and 4B). Next, we investigated the regulatory mechanism by which coronavirus decreases hnRNPC abundance. We treated different cells with actinomycin D (ActD) at different time points post infection, measured hnRNPC mRNA levels, calculated the half-life of the target RNA, and evaluated its stability (Fig 4C). We found that the hnRNPC mRNA half-life under mock conditions was 20.0 hours, while it was 2.8 hours in HCoV-OC43-infected cells (Fig 4D). There was no significant difference in the decay rate of GAPDH mRNA between the two conditions (Fig 4D). Similarly, HCoV-229E infection shortened the half-life of hnRNPC mRNA but did not significantly affect GAPDH mRNA (Fig 4E). To determine whether coronavirus infection affects hnRNPC protein stability, we monitored hnRNPC protein decay following the addition of the protein synthesis inhibitor cycloheximide (CHX) (Fig 4F). We found that hnRNPC protein abundance remained stable after CHX treatment during HCoV-OC43 and HCoV-229E infection (Fig 4G and 4H). These results suggest that the decrease in hnRNPC expression after coronavirus infection may be related to virus-facilitated mRNA decay.

### hnRNPC intracellular localization is regulated by coronavirus infection

**Fig 5:**
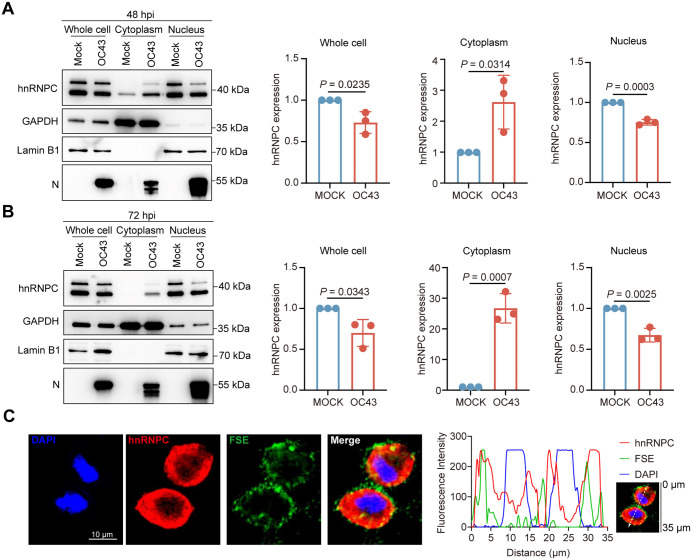
HCoV-OC43 infection facilitates hnRNPC entry into the cytoplasm. **(A-B)** Protein levels measured by immunoblotting in whole cell, nuclear and cytoplasmic fractions of H460 cells infected with HCoV-OC43 (MOI = 0.01) at 48 hpi (**A**) and 72 hpi **(B)**. GAPDH and Lamin B1 served as cytoplasmic and nuclear endogenous controls, respectively. **(C)** Co-localization image assessed with specific probes against HCoV-OC43 FSE RNA (green) and the specific antibody against hnRNPC (red). Scale bar, 10 μm. Nuclei were stained with DAPI (blue). Fluorescence intensity was calculated using ImageJ software. Data were shown as mean  ±  s.d. with individual data points. Statistical analyses were performed using an unpaired, two-tailed Student’s *t-test.*

In normal cells, hnRNPC is predominantly localized in the nucleus, with only a subset translocating to the cytoplasm during the G2/M phase [34,35]. We further investigated whether coronavirus infection promotes hnRNPC entry into the cytoplasm and its co-localization with FSE RNA. Notably, analysis of whole cell lysates revealed that the overall protein level of hnRNPC was reduced upon HCoV-OC43 infection. However, subcellular protein fractionation assays from the same experiment showed an increase in cytoplasmic hnRNPC accompanied by a decrease in its nuclear levels at 48 and 72 hpi (Fig 5A and 5B). Fluorescence in situ hybridization (FISH) and immunofluorescence (IF) colocalization assays demonstrated that hnRNPC and HCoV-OC43 FSE RNA partially colocalize in the cytoplasm of H460 cells (Fig 5C). These findings suggest that hnRNPC translocates from the nucleus to the cytoplasm in coronavirus-infected cells and physically binds to the FSE RNA of coronavirus.

### hnRNPC directly promotes coronavirus frameshifting

**Fig 6:**
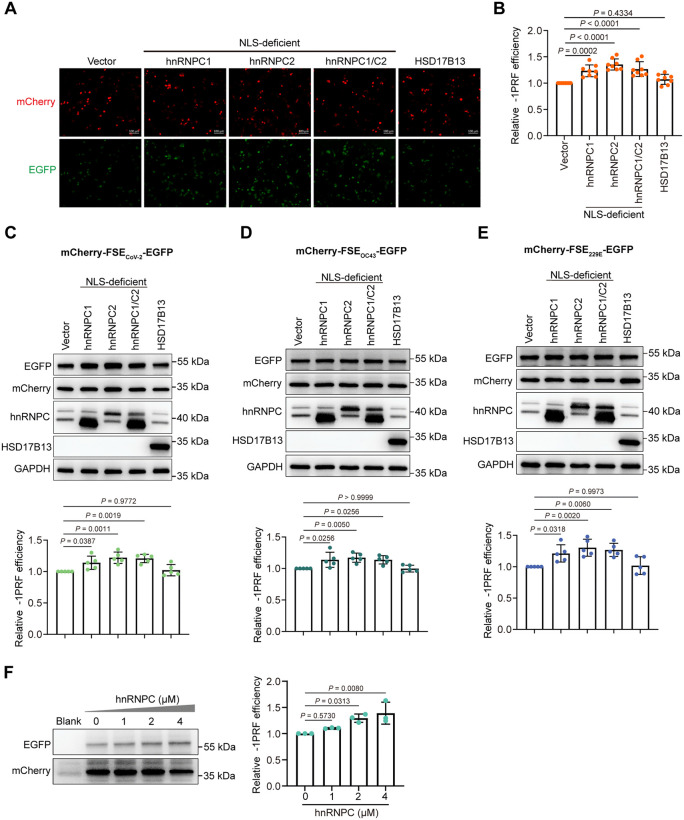
hnRNPC specifically promotes coronavirus frameshifting. (**A**) Representative images of mCherry-FSE_CoV-2_-EGFP reporters in Huh7 cells transfected with protein overexpression plasmid. Scale bar, 100 μm. (**B**) Effects of protein overexpression on mCherry-FSE_CoV-2_-EGFP reporters in Huh7 cells detected by fluorescence. (**C-E**) Protein levels measured by immunoblotting in Huh7 cells transfected with mCherry-FSE_CoV-2_-EGFP (**C**), mCherry-FSE_OC43_-EGFP (**D**), and mCherry-FSE_229E_-EGFP reporters (**E**), respectively. (**F**) Frameshifting reporter RNAs were translated in RRL in the presence of increasing concentrations of hnRNPC ranging from 0 to 4 µM and measured by immunoblotting. Data were shown as mean  ±  s.d. with individual data points. Statistical analyses were performed using one-way ANOVA followed by Dunnett’s post hoc comparison test.

We previously developed and used a dual fluorescent protein-based reporter to quantify SARS-CoV-2 –1 PRF efficiency [36]. To investigate the effect of hnRNPC on SARS-CoV-2 frameshifting, we targeted hnRNPC to the cytoplasm by disrupting its nuclear localization sequence (NLS). First, NLS-deficient hnRNPC was overexpressed in Huh7 cells; overexpression of 17-beta-hydroxysteroid dehydrogenase 13 (HSD17B13) served as a negative control, as it was not identified by RNA pull-down/LC-MS/MS. Huh7 cells were then transfected with the mCherry-FSE_CoV-2_-EGFP (-1) reporter, and the fluorescence intensity of mCherry and EGFP was examined using a microplate reader. The cells were subsequently harvested for protein expression analysis by immunoblotting. Using this fluorescence reporter assay, we observed that overexpressing NLS-deficient hnRNPC subunit or tetramer in Huh7 cells significantly increased SARS-CoV-2 –1 PRF (Fig 6A and 6B). We also measured mCherry and EGFP expression by immunoblotting and found increased levels of EGFP protein in NLS-deficient hnRNPC overexpression cells (Fig 6C). In contrast, overexpression of hnRNPC had no significant effect on the non-frameshifted control reporter, as determined by both fluorescence analysis and immunoblotting (S6 Fig). To determine whether hnRNPC-mediated enhancement of coronavirus –1 PRF is broad-spectrum, we examined its effect on –1 PRF of HCoV-OC43 and HCoV-229E. The results showed that hnRNPC promoted the efficacy of the –1 PRFs controlled by the different FSEs of HCoV-OC43 and HCoV-229E (Fig 6D and 6E), demonstrating broad-spectrum activity of hnRNPC in facilitating –1 PRF among coronaviruses. Furthermore, compared with wild-type hnRNPC, both hnRNPC^N7A^ and hnRNPC^N83A^ mutants exhibited a reduced ability to enhance –1 PRF efficiency of HCoV-OC43, whereas the hnRNPC^N7A/N83A^ double mutant completely lost this activity (S7 Fig), indicating that direct binding of hnRNPC to the FSE is essential for its ability to promote coronavirus –1 PRF efficiency. We next characterized hnRNPC mediated regulation of frameshifting *in vitro* using the rabbit reticulocyte lysate (RRL) translation system and recombinant hnRNPC. We observed a corresponding increase in –1 PRF. At the highest concentration of hnRNPC (4 µM), the efficacy of –1 PRF increased by 40% (Fig 6F), suggesting that hnRNPC acts directly on SARS-CoV-2 mRNA and does not require cofactors for its action.

We then analyzed whether hnRNPC facilitates the expression of viral non-structural proteins, which are essential for viral RNA replication. Ribosome profiling was performed on hnRNPC1, hnRNPC2 and HSD17B13 overexpressing Huh7 cells infected with HCoV-229E (S8A Fig). Interestingly, hnRNPC overexpression facilitated translation of all viral non-structural proteins (S8B Fig). Notably, the protein ratios of ORF1b/ORF1a-translated and RdRp/3CLpro increased after hnRNPC overexpression (S8C and S8D Figs). Taken together, these results further suggest that hnRNPC directly promotes coronavirus frameshifting.

### hnRNPC facilitates coronavirus replication

**Fig 7:**
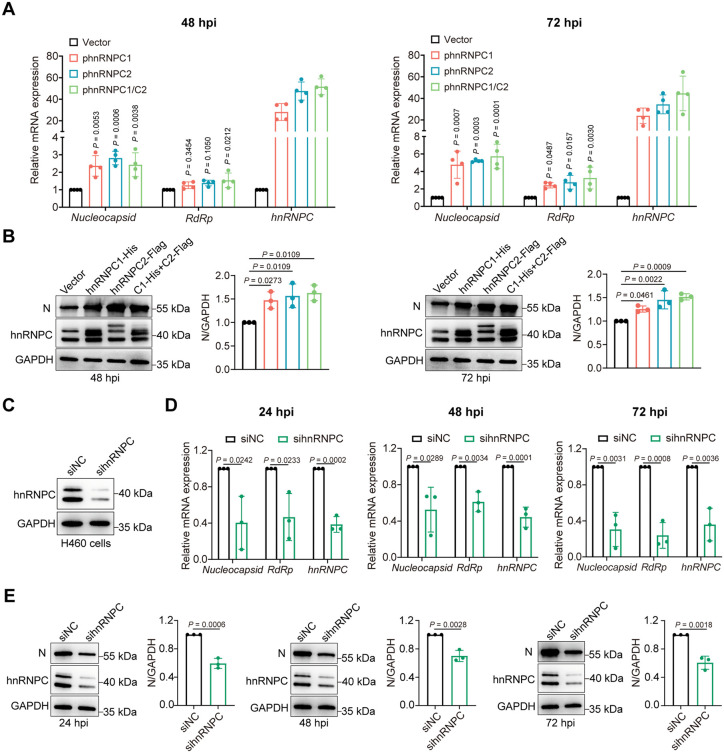
hnRNPC acts as a host cofactor for HCoV-OC43 infection. (**A-B**) RNA levels quantified by qRT-PCR (**A**) and protein levels detected by immunoblotting (**B**) in H460 cells after 48 and 72 hours of HCoV-OC43 infection (MOI = 0.01). (**C**) Protein levels measured by immunoblotting in H460 cells transfected with siRNA. (**D-E**) mRNA levels quantified by qRT-PCR (**D**) and protein levels detected by immunoblotting (**E**) in siRNA-treated H460 cells after HCoV-OC43 infection (MOI = 0.1) for 24, 48 and 72 hours. Data were shown as mean  ±  s.d. with individual data points. Statistical analyses were performed using one-way ANOVA followed by Dunnett’s post hoc comparison test (**A-B**); unpaired, two-tailed Student’s *t-test* (**D-E**).

In coronaviruses, –1 PRF is essential for efficient viral replication and transcription of the viral genome [37]. Therefore, we hypothesize that the interaction between hnRNPC and FSE RNA can affect coronavirus replication. We first measured the expression of HCoV-OC43 nucleocapsid and RdRp mRNA after overexpressing hnRNPC1, hnRNPC2, and hnRNPC1/C2 in H460 cells using qRT-PCR. The results showed that HCoV-OC43 nucleocapsid and RdRp mRNA levels were increased in hnRNPC-overexpressing cells compared with vector control cells at 48 and 72 hpi (Fig 7A). Consistently, HCoV-OC43 N protein levels were higher in hnRNPC-overexpressing cells (Fig 7B). As expected, all hnRNPC deletion mutants had little or no effect on HCoV-OC43 replication (S9 Fig). Moreover, the hnRNPC^N7A^ and hnRNPC^N83A^ mutants exhibited a markedly reduced ability to facilitate HCoV-OC43 replication, and the hnRNPC^N7A/N83A^ double mutant completely failed to facilitate viral replication (S10 Fig). To determine whether hnRNPC exhibits broad-spectrum activity for coronavirus replication, we further tested the effect of hnRNPC overexpression on viral infection using HCoV-229E. We observed higher viral mRNA and N protein expression in hnRNPC-overexpressing cells infected with HCoV-229E (S11A and 11B Fig).

We then knocked down hnRNPC in H460 and Huh7 cells using siRNA, and verified hnRNPC expression by immunoblotting (Figs 7C and S11C). qRT-PCR assays showed that hnRNPC knockdown significantly decreased HCoV-OC43 viral RNA levels at 24, 48 and 72 hpi (Fig 7D) in H460 cells. Consistently, these cells had lower N protein levels (Fig 7E). Similar results were observed in Huh7 cells infected with HCoV-229E (S11D and 11E Fig). Together, these results indicate that hnRNPC is essential for effective coronavirus replication and acts as a host cofactor for coronavirus.

### Elbasvir directly binds to hnRNPC, disrupting the interaction between hnRNPC and FSE RNA and specifically inhibiting coronavirus replication by decreasing –1 PRF efficiency

**Fig 8:**
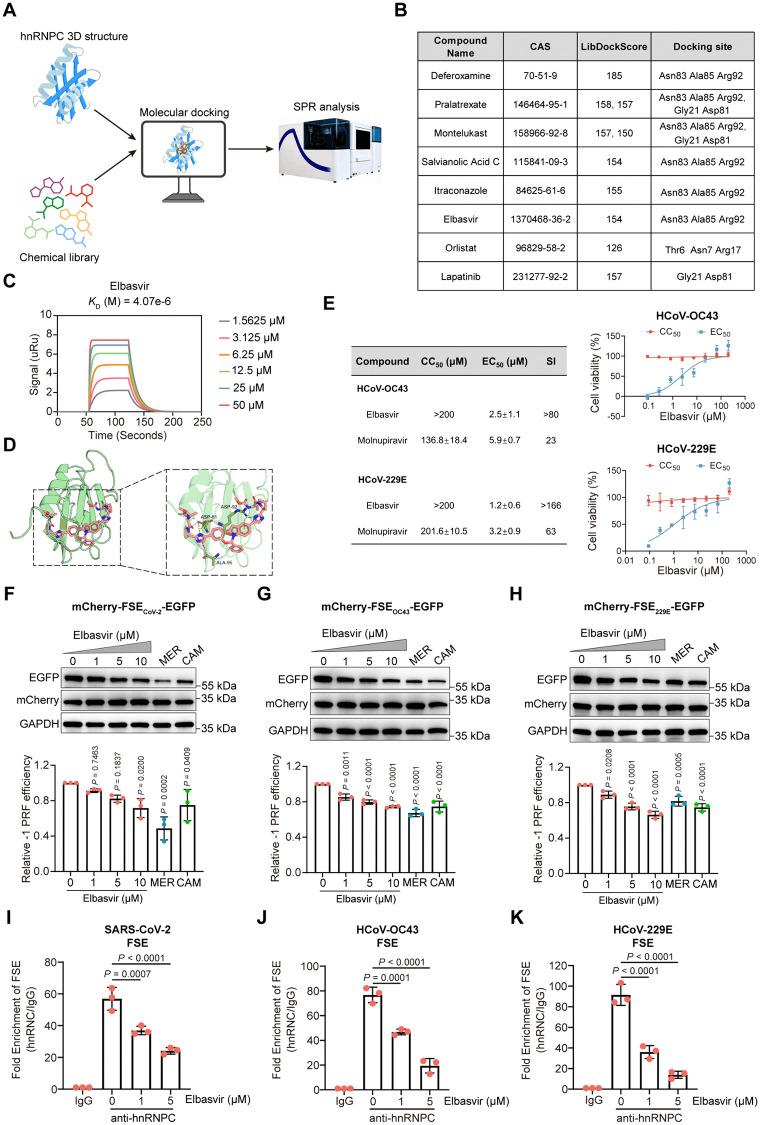
Elbasvir specifically inhibits –1 PRF efficiency of coronaviruses by blocking the binding of hnRNPC to FSE RNA. **(A)** Schematic representation of the high-throughput compound screening procedure. **(B)** Binding sites and LibDockScores of compound with hnRNPC. **(C)** Binding affinity between hnRNPC and Elbasvir analyzed by SPR. **(D)** Binding mode between Elbasvir and hnRNPC predicted by molecular docking. **(E)** Antiviral efficacy of Elbasvir against HCoV-OC43 and HCoV-229E detected by CCK-8 assay at 72 hours of drug treatment. MOI = 1. Molnupiravir was served as a positive drug control. CC_50_, half-maximal cytotoxic concentration. EC_50_, half-maximal effective concentration. SI, selection index (CC_50_ / EC_50_). **(F-H)** Elbasvir reduced the efficacy of –1 PRF reporters for SARS-CoV-2 **(F)**, HCoV-OC43 **(G)**, and HCoV-229E (**H**) in Huh7 cells detected by immunoblotting. Merafloxacin (MER) and Carrimycin (CAM) were served as positive drug controls. **(I-K)** FSE RNA levels of SARS-CoV-2 **(I)**, HCoV-OC43 **(J)**, and HCoV-229E (**K**) quantified by qRT-PCR after enrichment from RIP in Huh7 cells treated with Elbasvir. Data were shown as mean  ±  s.d. with individual data points. Statistical analyses were performed using one-way ANOVA followed by Dunnett’s post hoc comparison test.

Given the strong inhibition of HCoV-OC43 infection achieved by hnRNPC knockdown, and the ongoing evaluation of hnRNPC as a therapeutic target in SARS-CoV-2 infection, we established a model to screen small-molecule inhibitors of hnRNPC. Using DiscoveryStudio software, we selected five previously reported active pockets of hnRNPC [32] and performed molecular docking with small molecules from the commercial compound libraries L1000 and L6000. Compounds with a Libdockscore greater than 120 were further evaluated by surface plasmon resonance (SPR) analysis (Fig 8A). Based on docking scores and literature review, eight small molecules were selected for SPR assay (Fig 8B). SPR results showed that Deferoxamine, Pralatrexate, Montelukast, Salvianolic Acid C, and the known HCV NS5A inhibitor Elbasvir were capable of binding to hnRNPC, with Elbasvir exhibiting the highest binding activity, with a *K*_D_ value of 4.07 μM (Figs 8C and S12). No interaction between hnRNPC and the –1 PRF inhibitors Carrimycin [36] or Merafloxacin [38] was detected in the SPR assays (S12 Fig). Molecular docking analysis showed that three residues of the hnRNPC subunit (Asp81, Asp85 and Asp92) formed interactions with Elbasvir (Fig 8D).

To validate the antiviral activity of Elbasvir, we performed dose-response assays using CCK-8 staining. Elbasvir showed strong inhibitory activity against HCoV-OC43 and HCoV-229E without detectable cytotoxicity in H460 and Huh7 cells (Fig 8E), consistent with reports that Elbasvir inhibits SARS-CoV-2 in Vero E6 cells [39]. These results suggest that pharmacological inhibition of hnRNPC limits the replication of coronaviruses *in vitro*.

We then tested the effect of Elbasvir on coronavirus –1 PRF using the dual fluorescent reporter system. Elbasvir robustly inhibited –1 PRF efficiency in SARS-CoV-2 in a dose-dependent manner without affecting the non-frameshifted control reporter (Figs 8F and S13). Moreover, Elbasvir attenuated the hnRNPC-mediated enhancement of –1 PRF in SARS-CoV-2 (S14 Fig). Having observed its anti-frameshifting activity in SARS-CoV-2 PRF reporter assays, we next tested whether Elbasvir inhibits –1 PRF in HCoV-OC43 and HCoV-229E. Elbasvir inhibited –1 PRF of both coronaviruses with equal efficacy (Fig 8G and 8H). Interestingly, the inhibitory effect of Elbasvir on HCoV-229E –1 PRF was greater than that of Merafloxacin (Fig 8H). These results indicate that Elbasvir acts as a coronavirus –1 PRF inhibitor.

To further explore the mechanism by which Elbasvir inhibits coronavirus –1 PRF, we used RIP assays to determine whether Elbasvir blocks the binding between hnRNPC and FSE RNA. RIP experiments showed that the level of hnRNPC-associated viral FSE RNA progressively decreased with increasing concentrations of Elbasvir in Huh7 cells (Fig 8I-8K). In addition, Carrimycin blocked the binding between hnRNPC and FSE RNA by binding directly to the conserved viral FSE RNA pseudoknot [36], while Merafloxacin had no effect (S15 Fig). Since Elbasvir and Carrimycin block the interaction between hnRNPC and coronavirus FSE RNA through distinct binding sites, a synergistic antiviral assay against HCoV-OC43 and HCoV-229E with these two compounds was conducted using checkerboard microdilution assays. The combination of Elbasvir with Carrimycin exhibited additive effects against coronavirus, with zero interaction potency (ZIP) synergy scores of 2.419 ± 2.91 and 5.745 ± 5.35, respectively (S16 Fig). Collectively, these findings indicate that Elbasvir inhibits replication by blocking the interaction between hnRNPC and FSE RNA, thereby identifying hnRNPC as a host cofactor.

## Discussion

**Fig 9:**
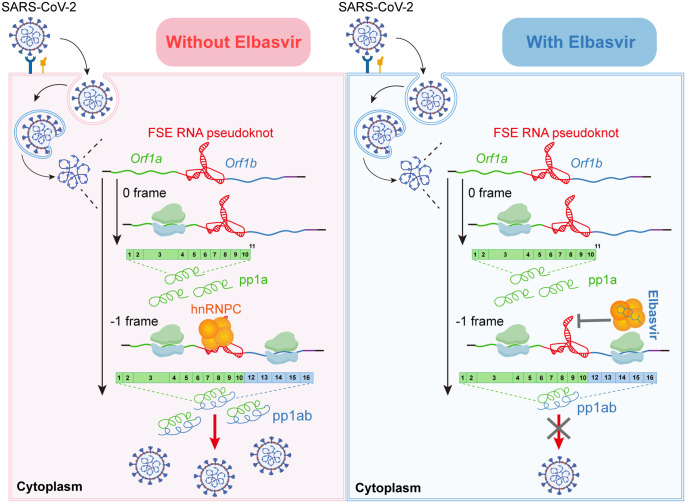
Schematic diagram of hnRNPC facilitating SARS-CoV-2 replication and serving as a therapeutic target. hnRNPC directly binds to the SARS-CoV-2 FSE RNA to promote SARS-CoV-2 –1 PRF and enhance viral replication (Left). Elbasvir inhibits SARS-CoV-2 replication by directly binding to hnRNPC and blocking the interaction between hnRNPC and the viral FSE RNA (Right).

When viral RNA is released into the cytoplasm, it hijacks host cellular RNA-binding proteins involved in all stages of the mRNA life cycle to regulate translation and replication in the challenging environment of host cells [40]. hnRNPs have been shown to interact extensively with viral RNA, and several hnRNPs play key roles in the life cycle of coronaviruses and other positive-strand RNA viruses, facilitating RNA synthesis and translation [29,41–46]. In this study, we demonstrated that hnRNPC increases the efficiency of –1 PRF by binding directly to the conserved viral FSE RNA pseudoknot, resulting in the release of the viral protein translation switch from ORF1a to ORF1b. This increases the amount of core components of viral replication and transcription complexes, facilitating viral replication. Although the observed modulation of –1 PRF efficiency by hnRNPC is relatively modest (~1.2–1.5-fold), even small changes in frameshifting efficiency can significantly affect the stoichiometry of viral proteins, potentially resulting in non-linear effects on viral replication [14,47]. We also found that treatment with the hnRNPC antagonist Elbasvir reduced coronavirus replication (Fig 9). These findings highlight that hnRNPC is a cofactor of coronaviruses and suggest that pharmacological hnRNPC inhibitors may be therapeutically beneficial to prevent or reduce the impact of SARS-CoV-2 infection.

Previous studies have shown that the occurrence of –1 PRF is generally associated with interactions among host factors, viral proteins, and viral RNA elements. For example, in encephalomyocarditis virus, the viral 2A protein promotes –1 PRF by directly interacting with sequences near the FSE RNA slippery site [48]. Similarly, in porcine reproductive and respiratory syndrome virus, the viral NSP1b protein interacts with host poly(C)-binding proteins (PCBPs), thereby enhancing -2/-1 ribosomal frameshifting within ORF1a [18]. Recent studies have also shown that eIF2A and SLBP promote SARS-CoV-2 –1 PRF [19,20]. Notably, these previously identified factors exhibit distinct binding specificities to different regions of the SARS-CoV-2 FSE. eIF2A preferentially binds CG-rich RNA motifs, while SLBP shows affinity for SL1 and SL3 of the FSE. In addition, ZAP-S, another known FSE-interacting protein, specifically targets SL2 and SL3. In this study, we identified hnRNPC as a novel host factor that promotes coronavirus –1 PRF. hnRNPC directly binds to the FSE RNA and enhances the stability of its pseudoknot structure. hnRNPC displays a sequence preference for U-rich regions [32,33]. Consistent with this, SARS-CoV-2 FSE RNA contains U-rich sequences within the slippery site, SL1, and SL2 regions, all of which were found to be preferred binding sites of hnRNPC. Although the RNA recognition motif (RRM) domain of hnRNPC is generally considered the primary RNA-binding region [49–53], our truncation experiments showed that constructs containing only the RRM and basic domains failed to bind the FSE RNA, suggesting that recognition of the pseudoknot structure may depend on the full-length protein and the integrity of its tertiary structure.

Interestingly, we observed that coronavirus infection leads to a global reduction in hnRNPC protein levels, which may result from virus-induced mRNA decay. It is well established that coronavirus infection can activate RNase L [54–57], and both structural and non-structural viral proteins facilitate the decay of multiple host mRNAs [7,58,59]. Although the present study excluded the possibility that the decrease in hnRNPC protein levels is caused by altered protein stability, it did not directly measure the rate of hnRNPC nascent protein synthesis upon viral infection. Hence, the potential contribution of impaired translational efficiency to the reduction in hnRNPC protein levels remains to be considered. Therefore, the precise mechanism underlying the reduction of hnRNPC remains to be elucidated. Under normal physiological conditions, hnRNPC is predominantly localized in the nucleus. Previous studies have reported that under pathological conditions, such as cancer and heart disease, a portion of hnRNPC can relocalize to the cytoplasm [49,60]. Similarly, hnRNPC relocalization to the cytoplasm has been reported during Sindbis virus, poliovirus and human rhinovirus infections [61–63]. In line with these findings, we found that hnRNPC undergoes cytoplasmic re-localization following coronavirus infection. This observation may be linked to the ability of coronaviruses to arrest the host cell cycle at the G2/M phase [64–66], a condition under which certain nuclear proteins are known to accumulate in the cytoplasm. In addition, reports suggest that hnRNPC can be exported to the cytoplasm via nuclear transport proteins [67,68], although the detailed mechanisms governing this relocalization require further investigation.

Previous studies showed that hnRNPC is required for the stabilization and translation of target mRNAs [69–73]. Studies in poliovirus have shown that hnRNPC can function as an RNA chaperone to regulate viral RNA structure, promoting positive-strand RNA synthesis by binding to the anti-cloverleaf at the 3’ end of the negative-strand RNA. This binding stabilizes transient single-stranded regions and facilitates recruitment of the viral polymerase [46]. In addition, circRNA has been reported to be involved in the hnRNPC mediated CRK-mTOR pathway by interacting with hnRNPC, thus facilitating MERS-CoV and SARS-CoV-2 replication [43]. The data in this study reveal a novel role for hnRNPC in the modulation of FSE RNA and –1 PRF in coronavirus infections. These findings provide new insights into the molecular mechanisms by which hnRNPC contributes to viral replication. However, hnRNPC overexpression not only increased the orf1b/orf1a ratio but also elevated overall ribosome occupancy on orf1ab. This observation suggests that hnRNPC may also be involved in the general regulation of RNA translation due to its inherent function [41]. Therefore, while our findings indicate that hnRNPC promotes coronavirus replication by enhancing FSE-mediated –1 PRF efficiency, we cannot rule out the possibility that it may also contribute to viral replication through its broader roles in RNA metabolism, such as translational regulation. From a therapeutic standpoint, our *in vitro* experiments demonstrated that the hnRNPC antagonist Elbasvir suppressed replication of HCoV-OC43 and HCoV-229E, consistent with earlier reports on SARS-CoV-2 [39], suggesting that this compound may act as a broad-spectrum inhibitor of coronaviruses. In addition, we observed that combining Elbasvir with Carrimycin produced an additive antiviral effect, which offers a preliminary rationale for further exploring this combination as a potential antiviral strategy.

In summary, our findings indicate that coronavirus infection promotes the cytoplasmic relocalization of hnRNPC, where it binds to FSE RNA and enhances –1 PRF efficiency, contributing to viral replication. Moreover, pharmacological blockade or knockdown of hnRNPC significantly reduced coronavirus replication *in vitro*. Collectively, hnRNPC is a host cofactor of coronavirus and a candidate host target for developing broad-spectrum antivirals against coronaviruses.

## Materials and methods

### Cells, viruses, and reagents

Huh7 cells (human hepatocellular carcinoma cell line) were cultured in Dulbecco’s Modified Eagle Medium (Gibco, USA) supplemented with 10% (v/v) fetal bovine serum (FBS) (Gibco,16000–044) and 1% penicillin–streptomycin (Beyotime, C0222). H460 cells (human lung cancer cell line) were cultured in RPMI 1640 medium (Gibco, USA) supplemented with 10% FBS and antibiotics. Cells were cultured at 37°C in a 5% CO_2_ incubator (Thermo Fisher). Virus strains HCoV-229E (VR-740) and HCoV-OC43 (VR-1558) were purchased from ATCC and used as surrogates for SARS-CoV-2. Actinomycin D, Cycloheximide and Elbasvir were obtained from MedChemExpress (Shanghai, China). Merafloxacin was obtained from TargetMol (Shanghai, China). Carrimycin was provided by Professor Weiqing He (Peking Union Medical College, Beijing, China).

### Sample preparation and assay conditions for LC-MS/MS

Briefly, 8 × 10^7^ H460 or Huh7 cells were infected with HCoV-OC43 or HCoV-229E, then lysed in IP Lysis Buffer (Thermo, 87788) containing Protease and Phosphatase Inhibitor Cocktail (Thermo, 78440). RNA-binding proteins were enriched using the RNA-Protein Pull-Down Kit (Thermo, 20164) according to the manufacturer’s instructions.

For LC-MS/MS, eluted proteins were resuspended in 50 mM NH_4_HCO_3_ with 8 M urea to denature proteins. 10 mM DTT was added and incubated at 55°C for 30 min, followed by 15 mM IAA for 30 min in the dark. The urea concentration was then diluted to less than 1 M with 25 mM NH_4_HCO_3_. Trypsin was added for overnight digestion at 37°C. Peptides were desalted using C18 stage tips and lyophilized. LC-MS/MS was performed by Oebiotech Co., Ltd. (Shanghai, China) and analyzed as described previously [74]. Bioinformatic analysis was performed using OECloud tools (https://cloud.oebiotech.com).

### Immunoblotting

Lysates were prepared by removing the medium from the cells, washing once with PBS, and adding cold radioimmunoprecipitation assay (RIPA) buffer containing 1 mM benzamidine and phenylmethylsulfonyl fluoride. Protein samples were boiled for 10 min at 100°C. Total cell lysate (10–20 μg) was separated by sodium dodecyl sulfate (SDS) polyacrylamide gel electrophoresis and transferred to polyvinylidene difluoride membranes (Millipore). The membranes were blocked in 5% nonfat dry milk for 1 hour and incubated at 4°C overnight with the indicated primary antibodies: anti-hnRNPC (catalog no. sc-32308, Santa Cruz Biotechnology), anti-hnRNPC2 (catalog no. ab133607, Abcam), anti-Lamin B1 (catalog no. 13435S, CST), anti-EGFP (catalog no. ab184601, Abcam), anti-mCherry (catalog no. 26765–1-AP, Proteintech), anti-Flag (catalog no. 66008–4-Ig, Proteintech), anti-HCoV-229E N protein (catalog no. 40640-T62, SinoBiological), anti-HCoV-OC43 N protein (catalog no. 40643-T62, SinoBiological), anti-Actin (catalog no. ET1702–52, HUABio), or anti-GAPDH (catalog no. 60004–1-Ig, Proteintech). Membranes were washed three times with Tris-buffered saline containing 1% Tween 20, then incubated for 1 hour at room temperature with horseradish peroxidase-conjugated secondary antibodies (goat anti-rabbit; catalog no. ZB-2301, ZSGB-BIO; goat anti-mouse; catalog no. ZB-2305, ZSGB-BIO). Protein bands were detected using an ECL chemiluminescence kit (Millipore, catalog no. WBKLS0050) and imaged with a Chemidoc XRS1 instrument (Bio-Rad, CA, USA). Band intensities were analyzed using ImageJ software.

### qRT-PCR

Total RNA was isolated as described previously [75]. Quantitative real-time PCR (qRT-PCR) reactions were set up using the HiScript II One Step qRT-PCR SYBR Green Kit (Vazyme, Q221-01) according to the manufacturer’s instructions and analyzed on the QuantStudio 6 Flex Real-Time PCR System (Thermo, 4485689) under the following cycling conditions: 50°C for 15 min; 95°C for 30 s; 40 cycles of 95°C for 10 s, and 60°C for 34 s. Fold change in mRNA expression was determined using the 2^^-ΔΔCt^ method relative to uninfected samples, after normalization to the housekeeping gene *GAPDH* (geometric mean). Statistical analysis was conducted by comparing ΔCt values of the respective RNA in uninfected and infected cells and results were plotted using Prism 9.2.0 (GraphPad). Primer sequences are listed in S2 Table.

### *In vitro* transcription RNA assay

FSE DNA [36] was obtained by PCR amplification using the primers (F: 5’-CTTATCGAAATTAATACGACTCACTATAGGG-3’; R: 5’-CCATAGAGCCCACCGCAT-3’) and purified with the TIANgel Purification Kit (TIANGEN, DP209). The purified PCR products were used for in vitro transcription with the MEGAscript Kit (Thermo, AM1333) according to the manufacturer’s protocol.

### Biotinylated RNA pulldown assay

RNAs were biotinylated using the Pierce RNA 3’ End Desthiobiotinylation Kit (Thermo, 20163). Negative RNA [poly(A)_25_] and non-related RNA (5’-CCUGGUUUUUAAGGAGUGUCGCCAGAGUGCCGCGAAUGAAAAA-3’) served as controls. RNA-binding proteins (RBPs) were enriched with the RNA-Protein Pull-Down Kit (Thermo, 20164). Briefly, biotinylated RNAs were incubated with streptavidin magnetic beads for 30 minutes at room temperature. RNA-bound beads were then incubated with 200 μg clarified protein lysate in 100 μL binding buffer and rotated for 60 minutes at 4°C. After incubation, the beads were washed three times with wash buffer, and elution buffer was added to the beads, followed by incubation at 37°C for 30 minutes with agitation. RNA-bound hnRNPC proteins were analyzed by immunoblotting.

### RNA immunoprecipitation (RIP)

Huh7 cells were transfected with IVT RNAs, and cells were collected for RIP experiments 16 hours post transfection. Two million cells were used for each IP. FSE RNA was enriched using the RIP Assay Kit (Beyotime, P1801S). Briefly, cells were lysed in 300 μL lysis buffer, centrifuged, and the supernatant was collected. 10% of the lysate was saved as whole cell lysate input. 1 μg specific antibody was incubated with protein A/G agarose at 4°C for 4 hours. Next, the cell lysate supernatant and the prepared complex were rotationally incubated at 4°C for 4 hours. After incubation, the agarose was washed four times with NT2 wash buffer and resuspended in 100 μL elution buffer. RNAs were isolated as described previously and reverse transcribed with the HiScript IV All-in-One Ultra RT SuperMix for qPCR (Vazyme, R433). qPCR analysis was subsequently performed with Taq Pro Universal SYBR qPCR Master Mix (Vazyme, Q712).

### Cytoplasmic/nuclear protein extraction

Cytosolic and nuclear proteins were extracted using the Nuclear and Cytoplasmic Protein Extraction Kit (Beyotime, P0028) according to the manufacturer’s protocol.

### FISH and IF staining

The HCoV-OC43 FSE RNA probe was designed and synthesized by Servicebio (Wuhan, China). The probes were used to detect the location of FSE RNA in H460 cells with a FISH Kit (Servicebio, China) according to the manufacturer’s instructions. For IF, cells grown on glass coverslips were fixed with 4% paraformaldehyde (PFA, Sigma Aldrich), permeabilized with 0.2% Triton X-100, blocked with BSA and incubated with anti-hnRNPC antibody (1:100, Santa Cruz, USA) at 4°C overnight. Cells were then incubated for 1 hour with the secondary antibody CY3-conjugated goat anti-mouse IgG (H  +  L) (1:300, Servicebio, China) at 37°C. DAPI was used to stain the nuclei of H460 cells. Sections were observed and images were collected using a Nikon upright fluorescence microscope (NIKON ECLIPSE CI, Japan).

### SPR assay

SPR analysis was performed using a Reichert 4SPR system (Reichert Technologies, USA) with SR7000 gold sensor chips featuring a Ni-NTA surface (Reichert, 3206063). The chip was pretreated with 40 mM nickel sulfate (Sigma, 656895), and 40 μg of recombinant human hnRNPC (FineTest, P8191) was immobilized via Ni-NTA interaction. FSE RNA sequences were amplified from viral FSE plasmids with the primers (5’-CTTATCGAAATTAATACGACTCACTATAGGG-3’ and 5’-CCATAGAGCCCACCGCAT-3’) using PCR and Phanta UniFi DNA Polymerase (Vazyme, P516). The RNAs were then transcribed *in vitro* using the MEGAscript Kit (Thermo, AM1333). The RNA sequences are listed in S4 Table. SPR data were processed and analyzed using TraceDrawer (Ridgeview Instruments AB). The *K*_D_ (M) value was determined using the OneToOne model of kinetic evaluation.

### mRNA stability assays

H460 or Huh7 cells (2 × 10^5^ cells per well) were seeded in 12-well plates and cultured for 24 hours. The cells were then inoculated with HCoV-OC43 or HCoV-229E at an MOI of 1 at 37°C or mock-infected. At 24 hours post-infection, mock-infected and coronavirus-infected cells were washed with PBS before treatment with actinomycin D (ActD) (10 μg/mL) prepared in complete cell medium for the indicated times. Total RNA was isolated from infected cells at the indicated time points using the RaPure Total RNA Micro Kit (Magen, R4111-03). QRT-PCR was performed with the HiScript II One Step qRT-PCR SYBR Green Kit (Vazyme, Q221) using the QuantStudio 6 Flex Real-Time PCR System. Statistical analysis normalized the average Ct value of each time point to the average Ct value at t = 0 to obtain the ∆Ct value (∆Ct = average Ct at each time point - average Ct at t = 0). The relative abundance at each time point was then calculated.

### Protein stability assays

H460 or Huh7 cells (4 × 10^5^ cells per well) were seeded in 6-well plates and cultured for 24 hours. The cells were then inoculated with HCoV-OC43 or HCoV-229E at an MOI of 1 at 37°C or mock-infected. At 24 hours post-infection, both mock-infected and coronavirus-infected cells were washed with PBS before treatment with cycloheximide (CHX) (100 μg/mL) prepared in complete cell medium for the indicated times. Cells were then lysed in RIPA buffer with protease inhibitors for immunoblotting using the indicated antibodies.

### Constructs and cell transfection

Full-length hnRNPC plasmids (hnRNPC1-his and hnRNPC2-Flag) were constructed by MIAOLING BIOLOGY (Wuhan, China). Truncated and mutant hnRNPC plasmids and mCherry-viral FSE-EGFP plasmids were constructed by Taihe Biotechnology Company (Beijing, China). Plasmids were transfected into Huh7 cells using Lipofectamine 3000 (Thermo, L3000015). Specific small interfering RNA (siRNA) oligonucleotides targeting hnRNPC were synthesized by Sangon Biotech (Shanghai, China). Lipofectamine RNAiMAX (Thermo, 13778150) was used to transfect siRNAs according to the manufacturer’s instructions. The siRNA sequences are listed in S3 Table.

### –1 PRF efficacy assay in cells

Huh7 cells (4 × 10^5^ cells per well) were seeded in 6-well plates and cultured for 24 hours. The cells were then transiently transfected with a –1 PRF construct encoding the dual-fluorescence mCherry-EGFP translation reporter. Images were acquired using a Zeiss Axio Observer 7 (Carl Zeiss, Germany) and analyzed with Zen 3.10 Edition software (Carl Zeiss). Fluorescence intensity (FI) was measured at λ_ex = 488 nm and λ_em = 528 nm for EGFP, and at λ_ex = 561 nm and λ_em = 610 nm for mCherry, using a microplate reader SpectraMax iD5 (Molecular Devices, USA). The ratio of EGFP to mCherry fluorescence intensity (E/m) was calculated as follows: –1 PRF efficiency = EGFP_intensity_ ⁄ mCherry_intensity_. The cells were then harvested for immunoblotting, and –1 PRF efficiency was calculated as previously described [76] using the formula: Density of EGFP band ⁄ (Density of EGFP band + Density of mCherry band).

### –1 PRF efficacy assay in rabbit reticulocyte lysate

The plasmid (pcDNA3.1(+)-mCherry-FSE_CoV2_-EGFP) was digested with EcoR I (NEB, R0101S) at 37°C for 3 hours and then treated with Mung Bean Nuclease (NEB, M0250S) at 30°C for 30 minutes. The digestion products were purified using the Wizard SV Gel and PCR Clean-Up System (Promega, A9282) according to the manufacturer’s instructions. The purified linear DNA was used as a template for in vitro transcription with the MEGAscript Kit (Thermo Fisher, AM1333). Transcribed RNA was purified using the Monarch RNA Cleanup Kit (NEB, T2040L) and subsequently used for *in vitro* translation with the RRL TNT T7 Quick Coupled Transcription/Translation System (Promega, L1170). Typical reactions contained 75% (v/v) RRL, 20 μM methionine, 1.5 μg template mRNA, and recombinant hnRNPC protein (OriGene, TP315956) dissolved in nuclease-free water at final concentrations ranging from 0 to 4 μM. Translation products were analyzed by immunoblotting.

### Ribosome profiling

Ribosome profiling was performed by CloudSeq Inc. (Shanghai, China) following the GenSeq Ribo Profile Kit manual (GenSeq, Inc.). Briefly, cycloheximide-treated Huh7 cells were lysed with lysis buffer and digested with nuclease. Ribosome footprints were purified by size-exclusion chromatography, and ribosome-protected fragments were size-selected by polyacrylamide gel electrophoresis (PAGE), followed by rRNA depletion. Purified RNA fragments were end-repaired, ligated with 3’ adapters and reverse-transcribed to cDNA. The cDNA products were purified by PAGE, circularized, and amplified by PCR. The resulting libraries were purified and sequenced on a NovaSeq platform (Illumina).

Paired-end sequencing reads were quality-filtered using a Q30 threshold. Adapter sequences and low-quality reads were removed with Cutadapt (v1.9.3). High-quality reads were aligned to the reference genome using TopHat2. Raw read counts were obtained with HTSeq (v0.9.1). Normalization and differential expression analyses were performed with edgeR, and differentially expressed mRNAs were identified based on p-values and fold-change criteria.

### Molecular docking

Molecular docking was performed using the GRAMM (Global RAnge Molecular Matching) [77] for protein-RNA docking and the LibDock module in Discovery Studio Client 2025 (BIOVIA, USA) for protein-ligand docking. The 3D structures of hnRNPC (PDB ID: 2MXY) and SARS-CoV-2 FSE RNA (PDB ID: 8VCI) were obtained from the RCSB database, while the commercial compound libraries L1000 and L6000 were provided by TargetMol (Shanghai, China). Docking results were visualized and analyzed using the PyMOL Molecular Graphics System (version 3.0.3). The protein structure was prepared by removing water molecules and heteroatoms, followed by the addition of hydrogen atoms and assignment of the CHARMm force field. For protein-ligand docking, the binding site was defined by a sphere (radius: 10 Å) centered on the key residues. For each ligand, multiple docking poses were generated and ranked by the LibDock score. The top-ranked compounds were further evaluated and selected based on binding mode, formation of key interactions (hydrogen bonding, hydrophobic contacts, etc.), and consistency with known structure-activity relationship (SAR) data.

### Antiviral assays

H460 or Huh7 cells (1.5 × 10^4^ cells per well) were seeded in 96-well plates and cultured for 24 hours. After the media was removed, the cells were infected with HCoV-OC43 or HCoV-229E (MOI of 1) and simultaneously treated with different dilutions of test compounds or positive control drugs. After 72 hours of incubation, OD_450_ values were measured using the CCK-8 assay. Drug interactions were analyzed using the ZIP synergy score in Synergy Finder, interpreted as follows: antagonistic (score < -10), additive (score between -10 and 10), and synergistic (score > 10).

### Statistical analyses

All statistical analyses were performed using GraphPad Prism version 9.5.0. Data are presented as mean   ±   standard deviation (s.d.) with individual data points. One-way analysis of variance (ANOVA) with Dunnett’s post hoc test was used for comparisons among multiple groups. Differences between two groups were compared using a two-tailed unpaired Student’s *t-test* or a two-tailed paired *t-test*. A *p* value less than 0.05 was considered statistically significant.

## Supporting information

S1 Fig Gene ontology (GO) term analysis and Kyoto Encyclopedia of Genes and Genomes (KEGG) term analysis results. (**A-B**) GO enrichment analysis (**A**) and KEGG enrichment analysis (**B**) of proteins that bind to SARS-CoV-2 FSE RNA in HCoV-OC43-infected H460 cells and HCoV-229E-infected Huh7 cells.
(TIF)

S2 Fig FSE sequence alignments of various coronaviruses. (TIF)

S3 Fig hnRNPC binds to coronaviral FSE RNAs. (**A**) Protein levels detected by immunoblotting in Huh7 cell lysates. Biotinylated IVT RNA was used in RNA pull-down assays. (**B-C**) Representative immunoblot examining hnRNPC protein in input and immunoprecipitation (IP) samples for RNA immunoprecipitation (RIP) assay (left panel). HCoV-OC43 FSE RNA (**B**) and HCoV-229E FSE RNA (**C**) were quantified by qRT-PCR after enrichment from RIP (right panel). (**D-E**) SPR analysis of affinity binding between hnRNPC and HCoV-OC43 (**D**) or HCoV-229E (**E**) FSE RNA. Data were shown as mean  ±  s.d. with individual data points. Statistical analyses were performed using an unpaired, two-tailed Student’s *t-test*.
(TIF)

S4 Fig Expression of Flag-tagged hnRNPC. Immunoblot analysis of Flag-tagged hnRNPC in Huh7 cells transfected with full-length or truncated hnRNPC plasmid (D1: 1–103 aa, D2: 1–140 aa, D3: 1–179 aa, D4: 1–207 aa, D5: 104–306 aa).
(TIF)

S5 Fig RNA binding activity of WT and mutated hnRNPC with HCoV-OC43 FSE RNA in Huh7 cells. HCoV-OC43 FSE RNA was quantified by qRT-PCR after enrichment from RIP. Data were shown as mean  ±  s.d. with individual data points. Statistical analyses were performed using one-way ANOVA followed by Dunnett’s post hoc comparison test.
(TIF)

S6 Fig Effects of protein overexpression on mCherry-EGFP control (lacking a –1 PRF element) and mCherry-FSE_CoV-2_-EGFP reporter in Huh7 cells. (**A**) Relative –1 PRF efficiency measured by fluorescence. (**B**) Relative –1 PRF efficiency measured by immunoblotting. Left panel, representative immunoblots. Right panel, quantification of relative –1 PRF efficiency. 17-beta-hydroxysteroid dehydrogenase 13 (HSD17B13) served as a negative control. Data were shown as mean  ±  s.d. with individual data points. Statistical analyses were performed using one-way ANOVA followed by Dunnett’s post hoc comparison test.
(TIF)

S7 Fig Effects of hnRNPC mutants on –1 PRF efficiency of HCoV-OC43. Protein levels measured by immunoblotting in Huh7 cells transfected with mCherry-FSE_OC43_-EGFP. Left panel, representative immunoblots. Right panel, quantification of relative –1 PRF efficiency. Data were shown as mean  ±  s.d. with individual data points. Statistical analyses were performed using one-way ANOVA followed by Dunnett’s post hoc comparison test.
(TIF)

S8 Fig Ribosome profiling sequencing. (**A**) Schematic representation of ribosome profiling sequencing (Ribo-seq). (**B-D**) Ribo-seq read counts within the genome of HCoV-229E gp1 (ORF1ab) (**B**), relative translation ratios of ORF1b to ORF1a (**C**), and relative translation ratios of RdRp to 3CL^pro^ (**D**) in protein overexpressing Huh7 cells at 24 hours post infection with HCoV-229E (MOI = 1). For panels (**C**) and (**D**), read counts were normalized to the length of the corresponding coding regions (per kilobase) prior to ratio calculation.
(TIF)

S9 Fig Analysis of the antiviral activity of hnRNPC deletion mutants on HCoV-OC43. RNA levels were quantified by qRT-PCR in H460 cells transfected with hnRNPC deletion mutant plasmids 48 h post infection with HCoV-OC43 (MOI = 0.01).
(TIF)

S10 Fig Effects of hnRNPC mutants on HCoV-OC43 replication. (**A-B**) RNA levels quantified by qRT-PCR (**A**) and protein levels measured by immunoblotting (**B**) in H460 cells after 48 hours of HCoV-OC43 infection (MOI = 0.01). Data were shown as mean  ±  s.d. with individual data points. Statistical analyses were performed using one-way ANOVA followed by Dunnett’s post hoc comparison test.
(TIF)

S11 Fig hnRNPC acts as a host cofactor for HCoV-229E infection. (**A-B**) RNA levels quantified by qRT-PCR (**A**) and protein levels detected by immunoblotting (**B**) in Huh7 cells after 48 and 72 hours of HCoV-229E infection (MOI = 0.01). (**C**) Protein levels measured by immunoblotting in Huh7 cells transfected with siRNA. (**D-E**) RNA levels quantified by qRT-PCR (**D**) and protein levels detected by immunoblotting (**E**) in siRNA-treated Huh7 cells after HCoV-229E infection (MOI = 0.1) for 24, 48 and 72 hours. Data were shown as mean  ±  s.d. with individual data points. Statistical analyses were performed using one-way ANOVA followed by Dunnett’s post hoc comparison test (**A-B**); unpaired, two-tailed Student’s *t-test* (**D-E**).
(TIF)

S12 Fig Binding affinity between hnRNPC and compound detected by SPR. (TIF)

S13 Fig Elbasvir reduced the efficacy of mCherry-FSE_CoV-2_-EGFP reporter but not the mCherry-EGFP control (lacking a –1 PRF element) in Huh7 cells detected by immunoblotting. Left panel, representative immunoblots. Right panel, quantification of relative –1 PRF efficiency. Data were shown as mean  ±  s.d. with individual data points. Statistical analyses were performed using an unpaired, two-tailed Student’s t-test.
(TIF)

S14 Fig Elbasvir reduced the efficacy of mCherry-FSE_CoV-2_-EGFP reporters detected by immunoblotting in Huh7 cells overexpressing hnRNPC2 NLS-deficient. (TIF)

S15 Fig RIP-qPCR analysis. FSE RNA levels of SARS-CoV-2 quantified by qRT-PCR after enrichment from RIP in Huh7 cells treated with Carrimycin (**A**) or Merafloxacin (**B**).
(TIF)

S16 Fig Elbasvir in combination with Carrimycin additively inhibited HCoV-OC43 replication in H460 cells (A) and HCoV-229E replication in Huh7 cells (B). Left panel: Two-dimensional representation of dose response interaction matrix. Right panel: ZIP model and synergy score of the combined administration of Elbasvir and Carrimycin. Data were shown with mean ± s.d., *n* = 3.
(TIF)

S1 Table Data information of host factors captured by SARS-CoV-2 FSE RNA. (DOCX)

S2 Table Primers for qRT-PCR. (DOCX)

S3 Table Sequences of siRNAs. (DOCX)

S4 Table RNA sequences. (DOCX)

S1 Data Data. (XLSX)

S1 File Rawgel. (PDF)
