# Supplementary material for: hnRNPC facilitates coronavirus replication by directly binding the frameshift-stimulatory element of viral genomic RNA
Source: PLoS Pathog. 2026 Aug 7;22(8):e1014491. doi: 10.1371/journal.ppat.1014491 (PMC13450726; doi:10.1371/journal.ppat.1014491)
Supplement: Supplementary file 1 [file ppat.1014491.s022.pdf]

Fig 1C

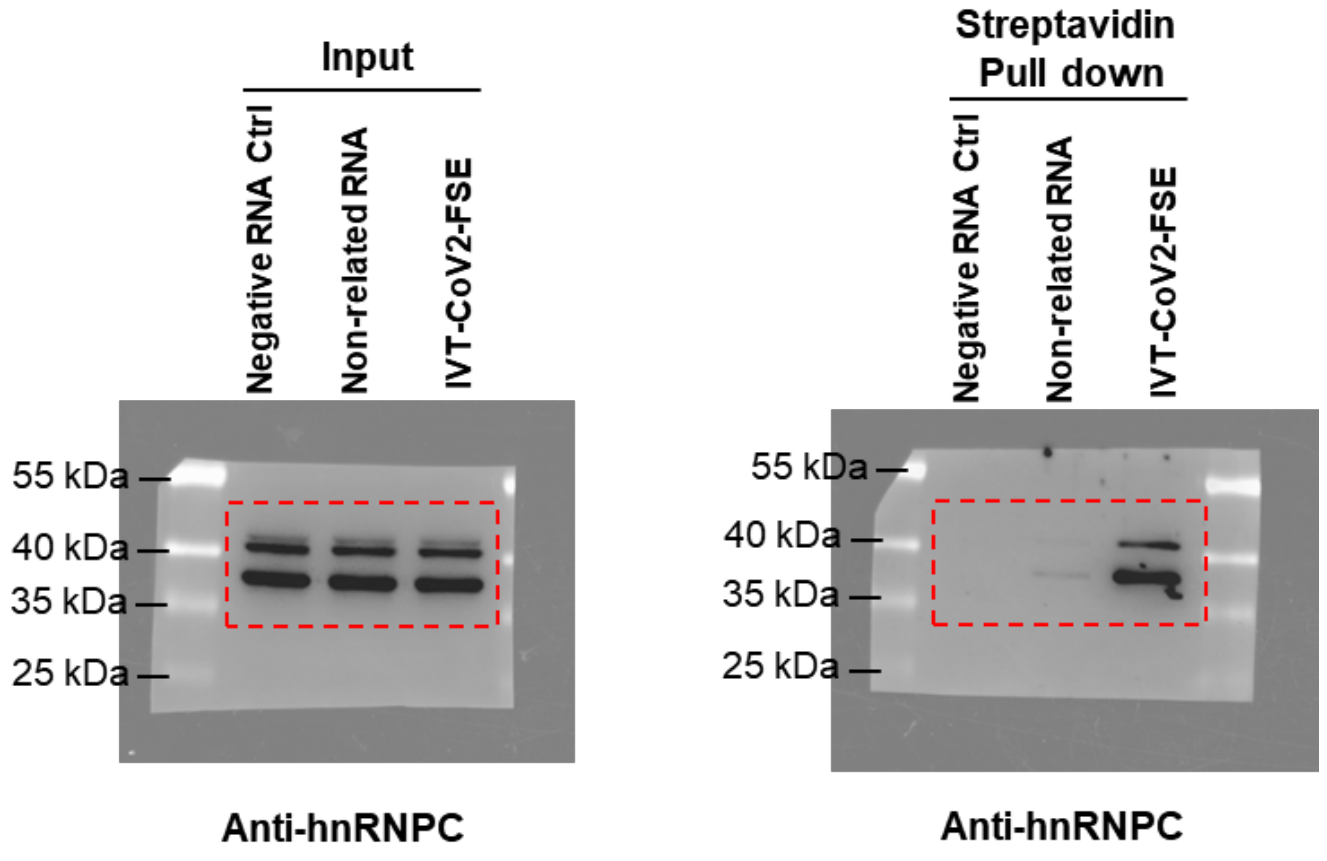

Fig 1E

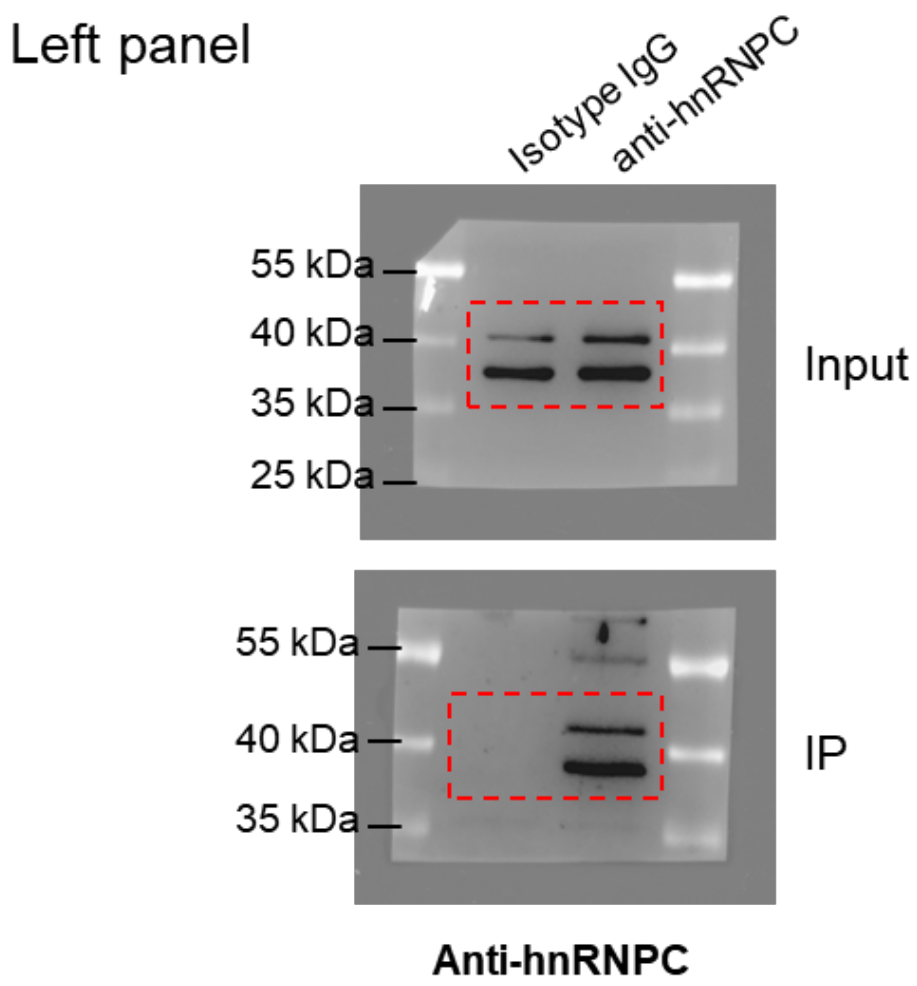

Fig 1F

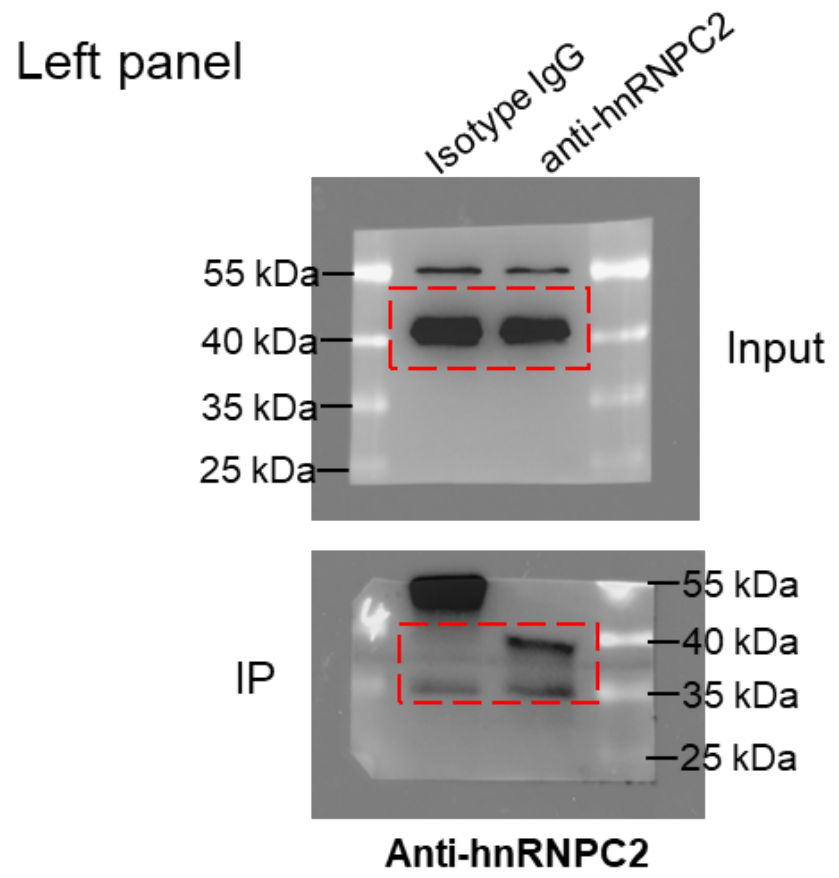

Fig 3C

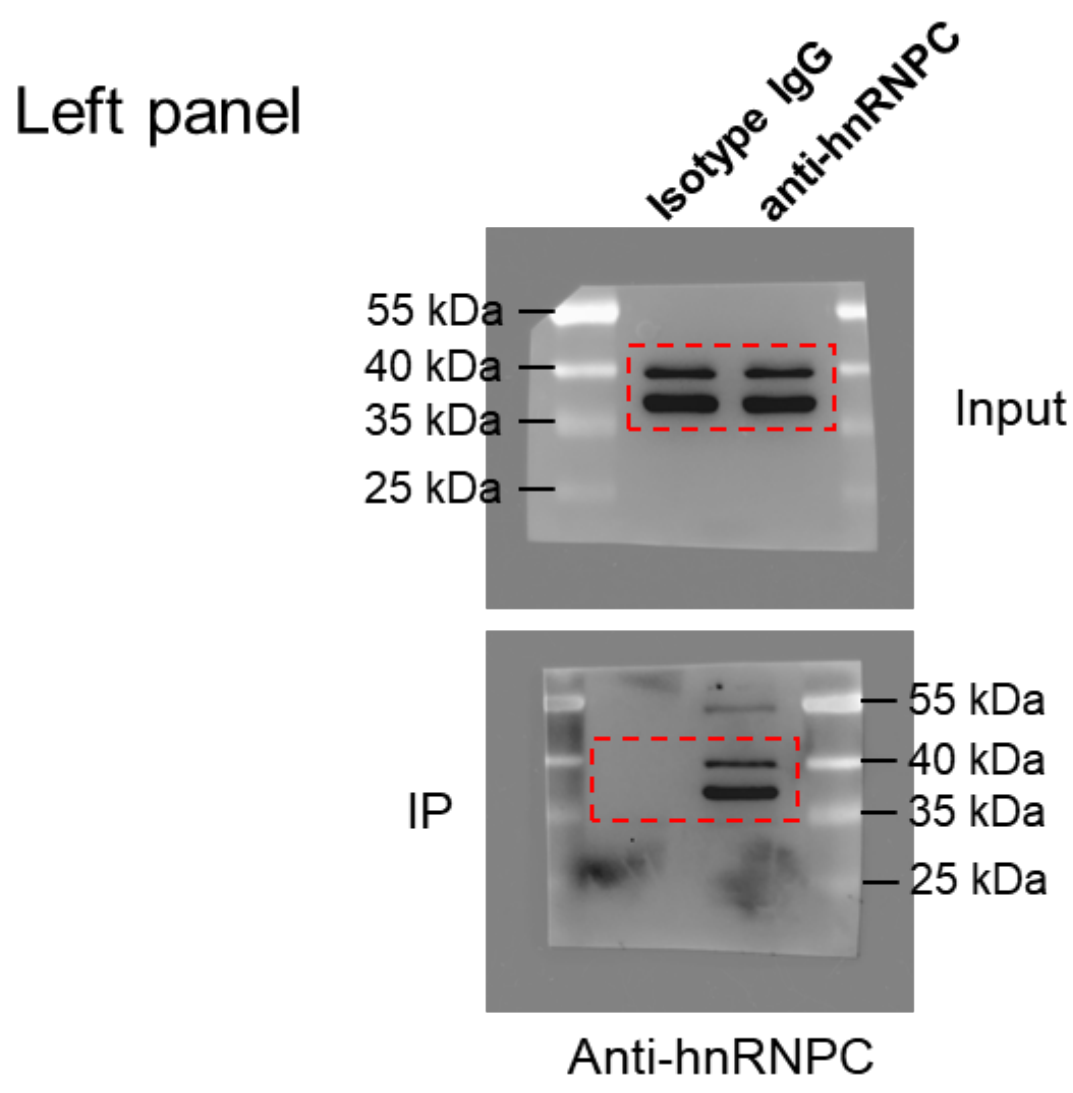

Left panel
Isotype IgG
anti-hnRNPC
55 kDa
40 kDa
35 kDa
25 kDa
Input
IP
55 kDa
40 kDa
35 kDa
25 kDa
Anti-hnRNPC

Fig 3D

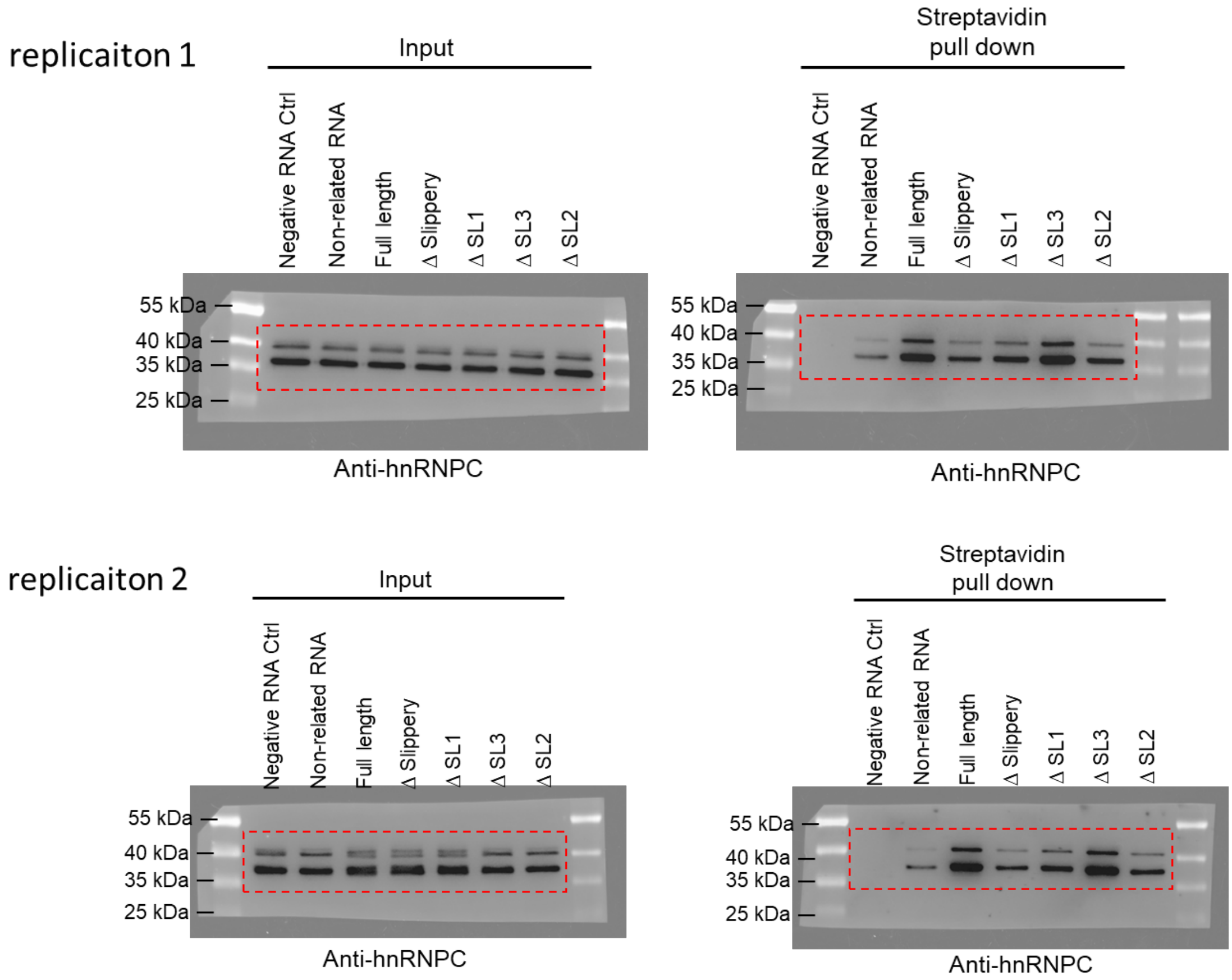

replicaiton 1
Input
Negative RNA Ctrl
Non-related RNA
Full length
Δ Slippery
Δ SL1
Δ SL3
Δ SL2
55 kDa
40 kDa
35 kDa
25 kDa
Anti-hnRNPC
Streptavidin pull down
Negative RNA Ctrl
Non-related RNA
Full length
Δ Slippery
Δ SL1
Δ SL3
Δ SL2
55 kDa
40 kDa
35 kDa
25 kDa
Anti-hnRNPC
replicaiton 2
Input
Negative RNA Ctrl
Non-related RNA
Full length
Δ Slippery
Δ SL1
Δ SL3
Δ SL2
55 kDa
40 kDa
35 kDa
25 kDa
Anti-hnRNPC
Streptavidin pull down
Negative RNA Ctrl
Non-related RNA
Full length
Δ Slippery
Δ SL1
Δ SL3
Δ SL2
55 kDa
40 kDa
35 kDa
25 kDa
Anti-hnRNPC

replicaiton 3

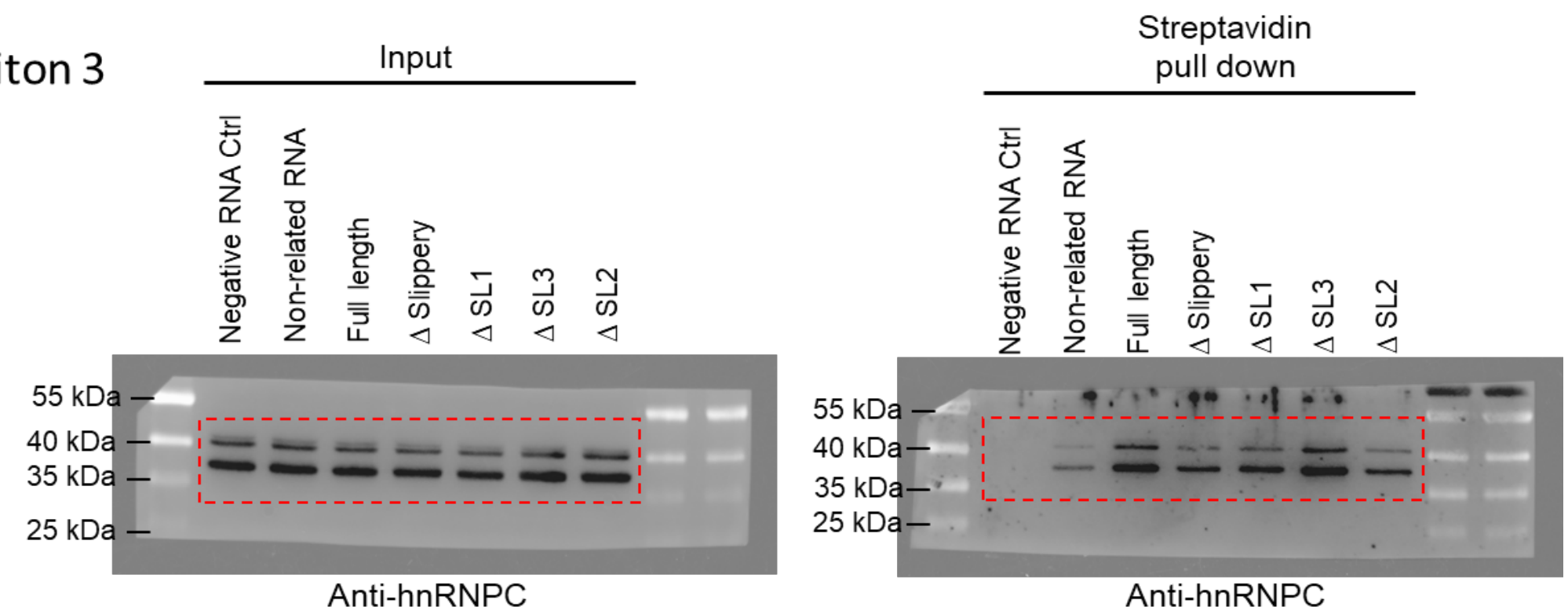

Fig 4A

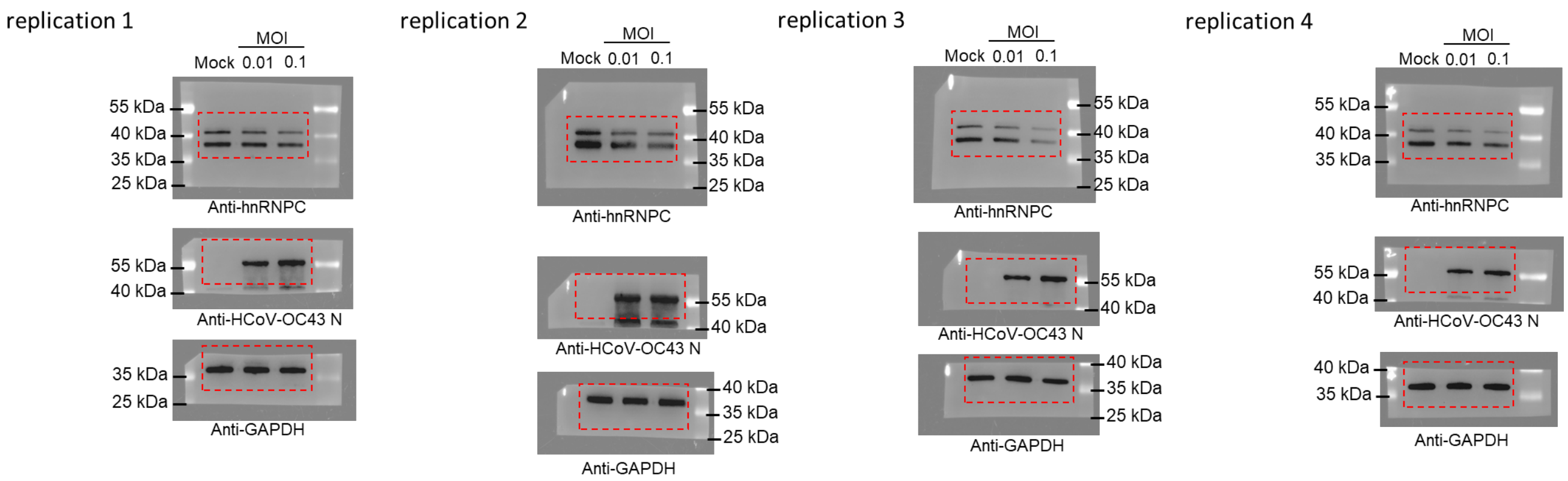

Fig 4B

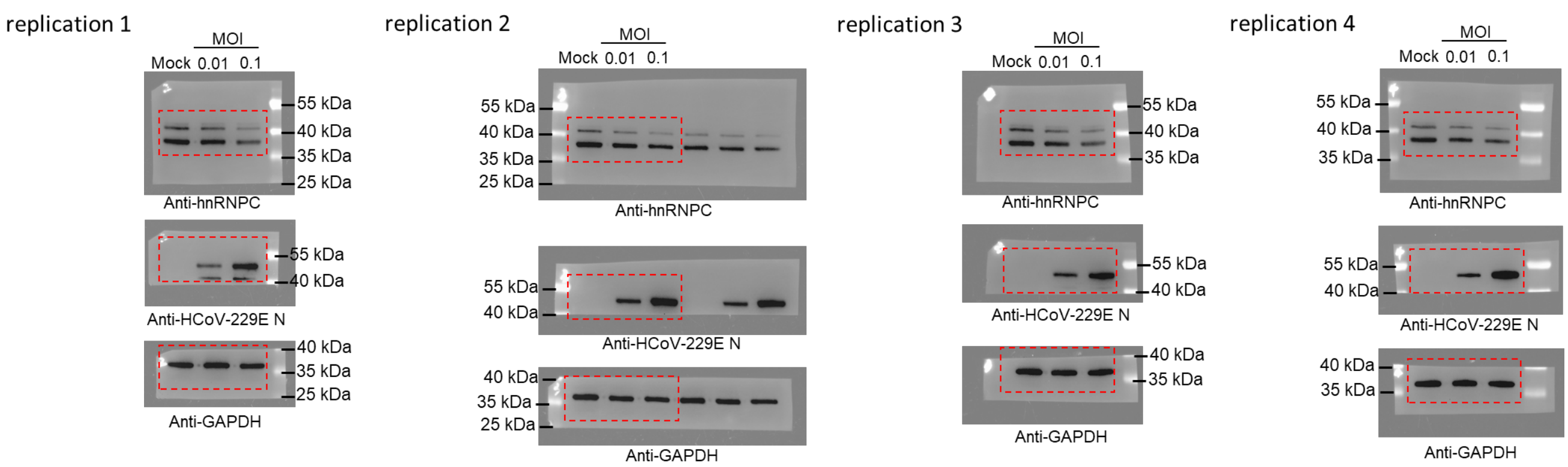

Fig 4G

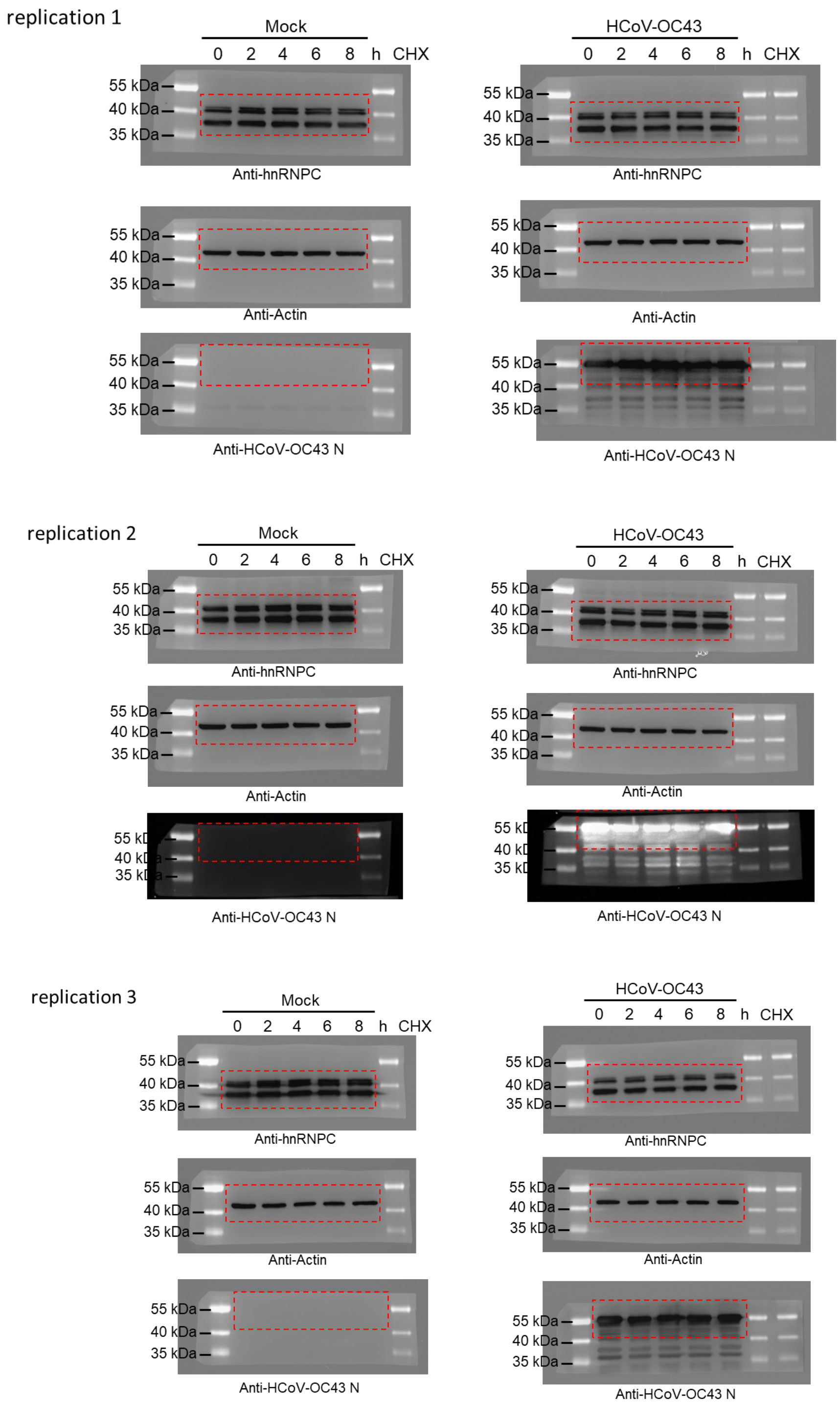
replication 1
Mock
0 2 4 6 8 h CHX
HCoV-OC43
55 kDa
40 kDa
35 kDa
Anti-hnRNPC
Anti-Actin
Anti-HCoV-OC43 N
replication 2
replication 3

Fig 4H

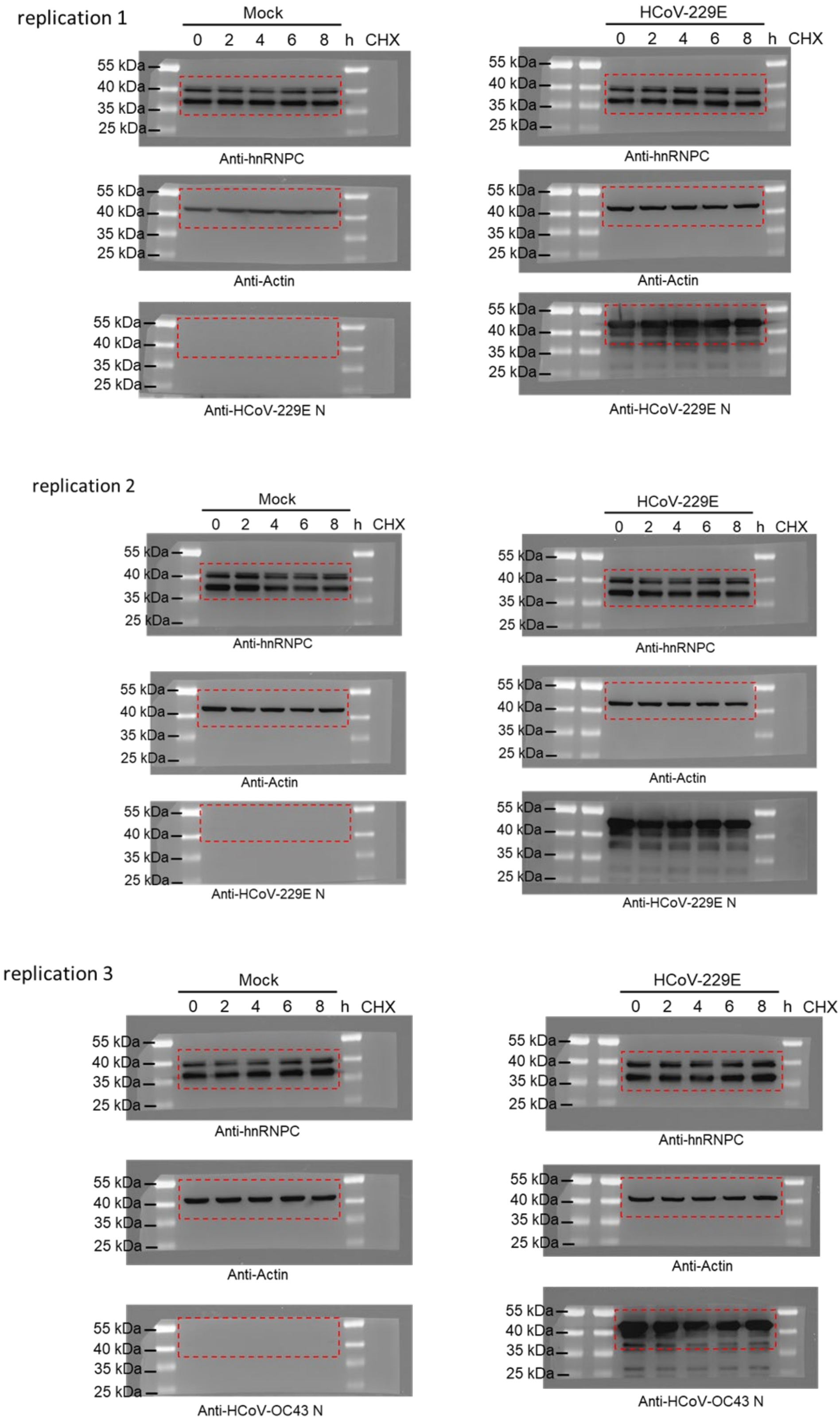
replication 1
Mock
0 2 4 6 8 h CHX
HCoV-229E
0 2 4 6 8 h CHX
55 kDa
40 kDa
35 kDa
25 kDa
Anti-hnRNPC
Anti-Actin
Anti-HCoV-229E N
replication 2
Mock
0 2 4 6 8 h CHX
HCoV-229E
0 2 4 6 8 h CHX
Anti-hnRNPC
Anti-Actin
Anti-HCoV-229E N
replication 3
Mock
0 2 4 6 8 h CHX
HCoV-229E
0 2 4 6 8 h CHX
Anti-hnRNPC
Anti-Actin
Anti-HCoV-OC43 N

Fig 5A

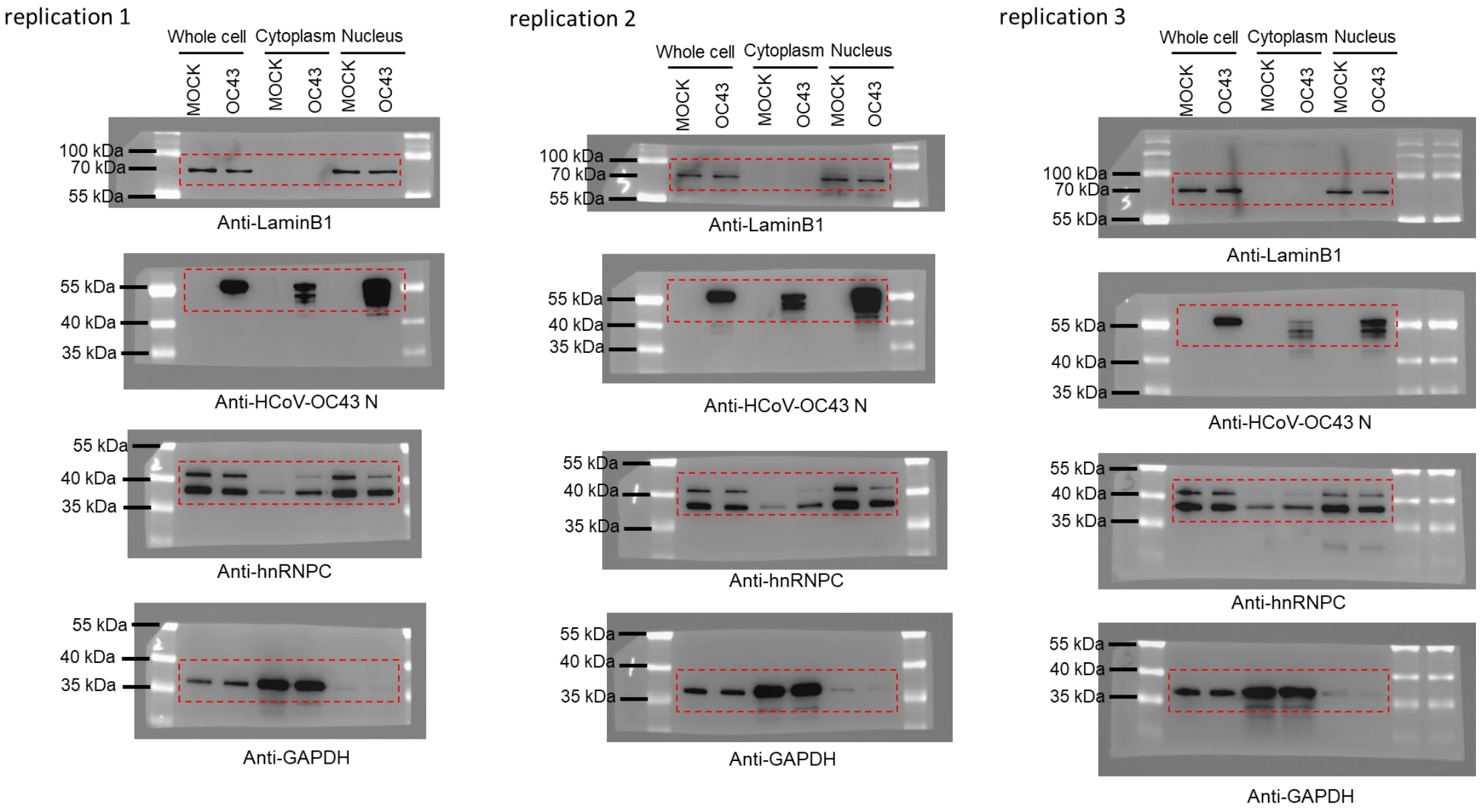
replication 1
Whole cell Cytoplasm Nucleus
MOCK OC43 MOCK OC43 MOCK OC43
100 kDa
70 kDa
55 kDa
Anti-LaminB1
55 kDa
40 kDa
35 kDa
Anti-HCoV-OC43 N
55 kDa
40 kDa
35 kDa
Anti-hnRNPC
55 kDa
40 kDa
35 kDa
Anti-GAPDH
replication 2
Whole cell Cytoplasm Nucleus
MOCK OC43 MOCK OC43 MOCK OC43
100 kDa
70 kDa
55 kDa
Anti-LaminB1
55 kDa
40 kDa
35 kDa
Anti-HCoV-OC43 N
55 kDa
40 kDa
35 kDa
Anti-hnRNPC
55 kDa
40 kDa
35 kDa
Anti-GAPDH
replication 3
Whole cell Cytoplasm Nucleus
MOCK OC43 MOCK OC43 MOCK OC43
100 kDa
70 kDa
55 kDa
Anti-LaminB1
55 kDa
40 kDa
35 kDa
Anti-HCoV-OC43 N
55 kDa
40 kDa
35 kDa
Anti-hnRNPC
55 kDa
40 kDa
35 kDa
Anti-GAPDH

Fig 5B

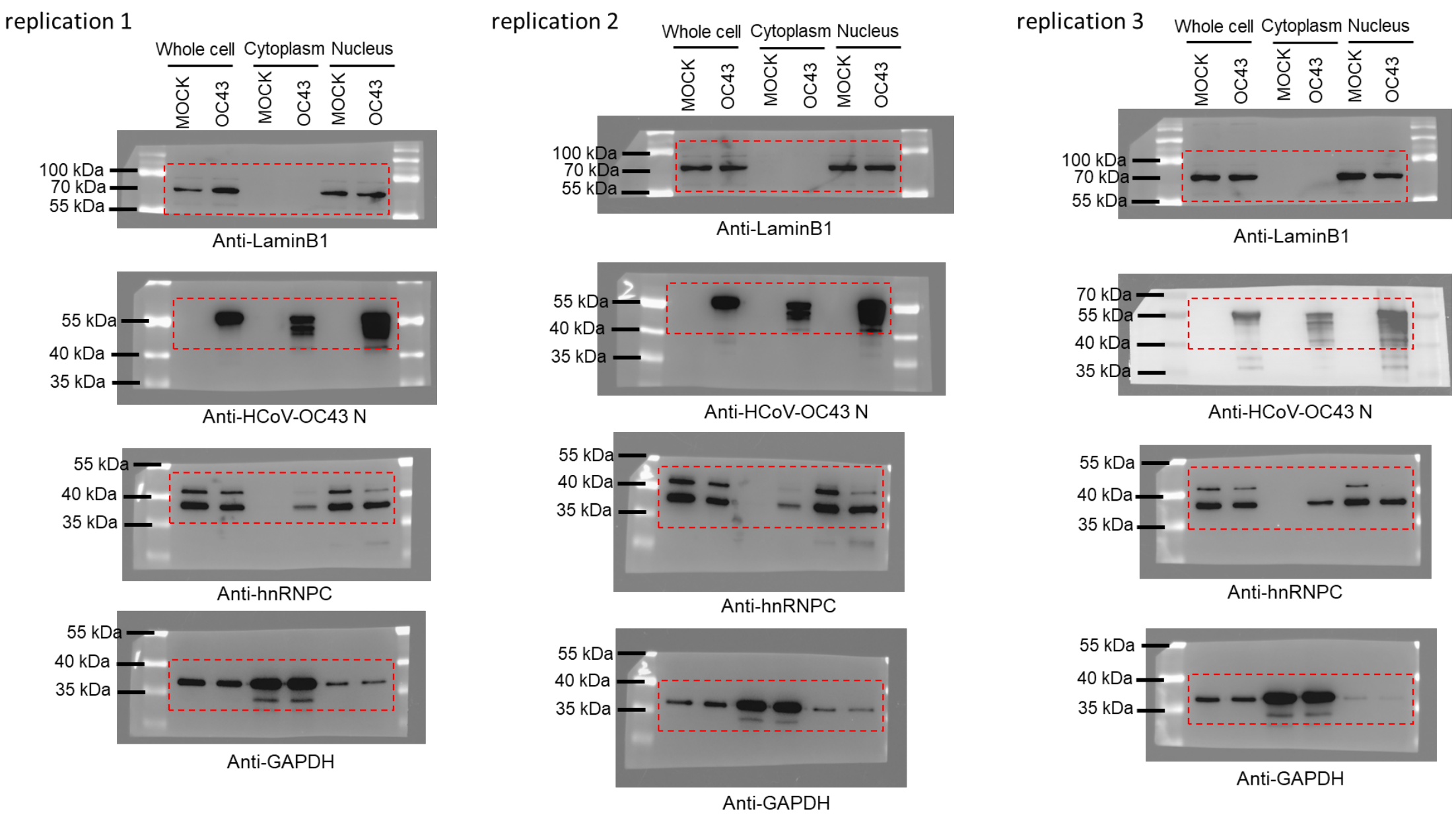
replication 1
Whole cell Cytoplasm Nucleus
MOCK OC43 MOCK OC43 MOCK OC43
100 kDa
70 kDa
55 kDa
Anti-LaminB1
55 kDa
40 kDa
35 kDa
Anti-HCoV-OC43 N
55 kDa
40 kDa
35 kDa
Anti-hnRNPC
55 kDa
40 kDa
35 kDa
Anti-GAPDH
replication 2
Whole cell Cytoplasm Nucleus
MOCK OC43 MOCK OC43 MOCK OC43
100 kDa
70 kDa
55 kDa
Anti-LaminB1
55 kDa
40 kDa
35 kDa
Anti-HCoV-OC43 N
55 kDa
40 kDa
35 kDa
Anti-hnRNPC
55 kDa
40 kDa
35 kDa
Anti-GAPDH
replication 3
Whole cell Cytoplasm Nucleus
MOCK OC43 MOCK OC43 MOCK OC43
100 kDa
70 kDa
55 kDa
Anti-LaminB1
70 kDa
55 kDa
40 kDa
35 kDa
Anti-HCoV-OC43 N
55 kDa
40 kDa
35 kDa
Anti-hnRNPC
55 kDa
40 kDa
35 kDa
Anti-GAPDH

Fig 6C

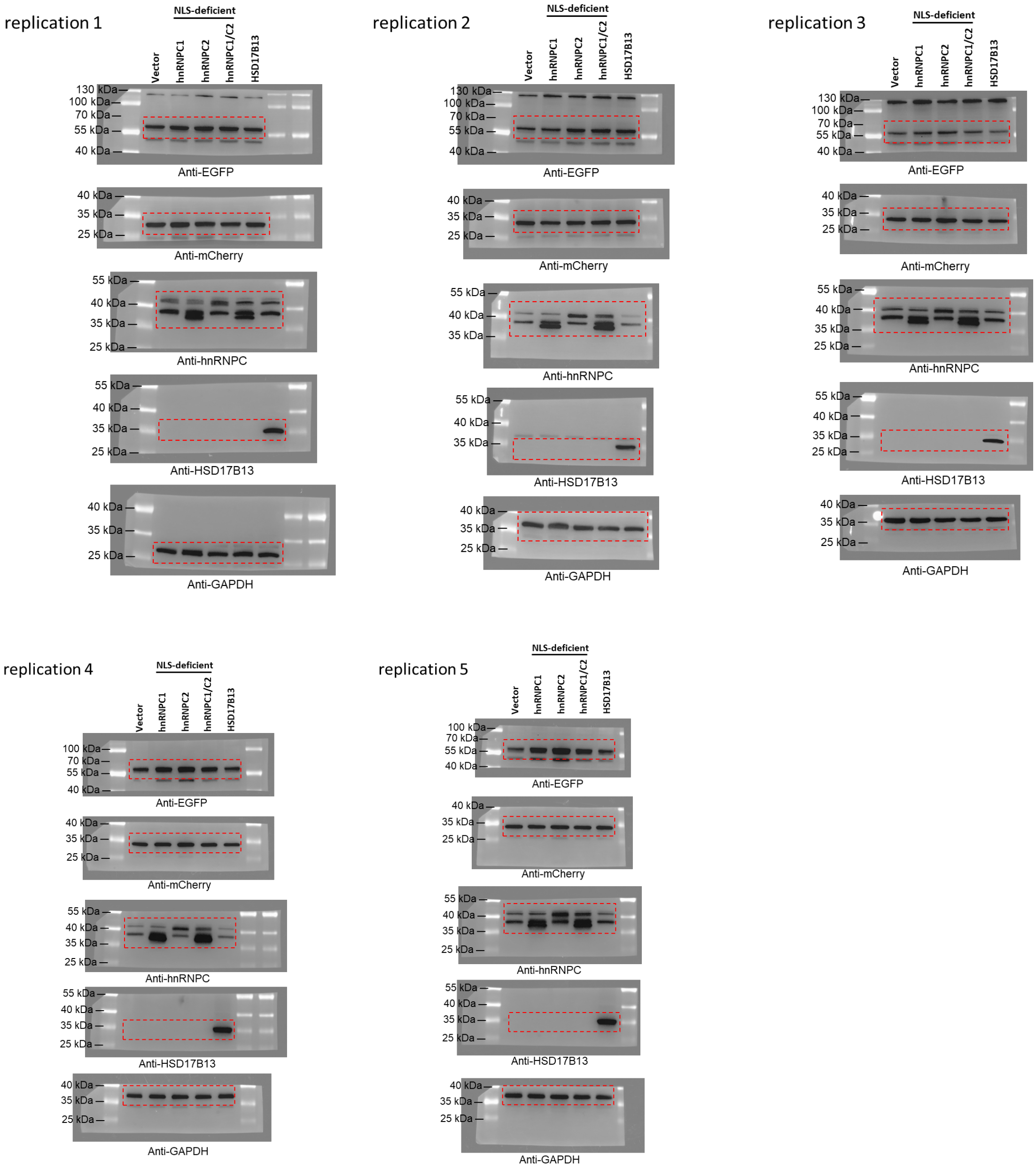
replication 1
replication 2
replication 3
replication 4
replication 5
NLS-deficient
Vector
hnRNPC1
hnRNPC2
hnRNPC1/C2
HSD17B13
130 kDa
100 kDa
70 kDa
55 kDa
40 kDa
35 kDa
25 kDa
Anti-EGFP
Anti-mCherry
Anti-hnRNPC
Anti-HSD17B13
Anti-GAPDH

Fig 6D

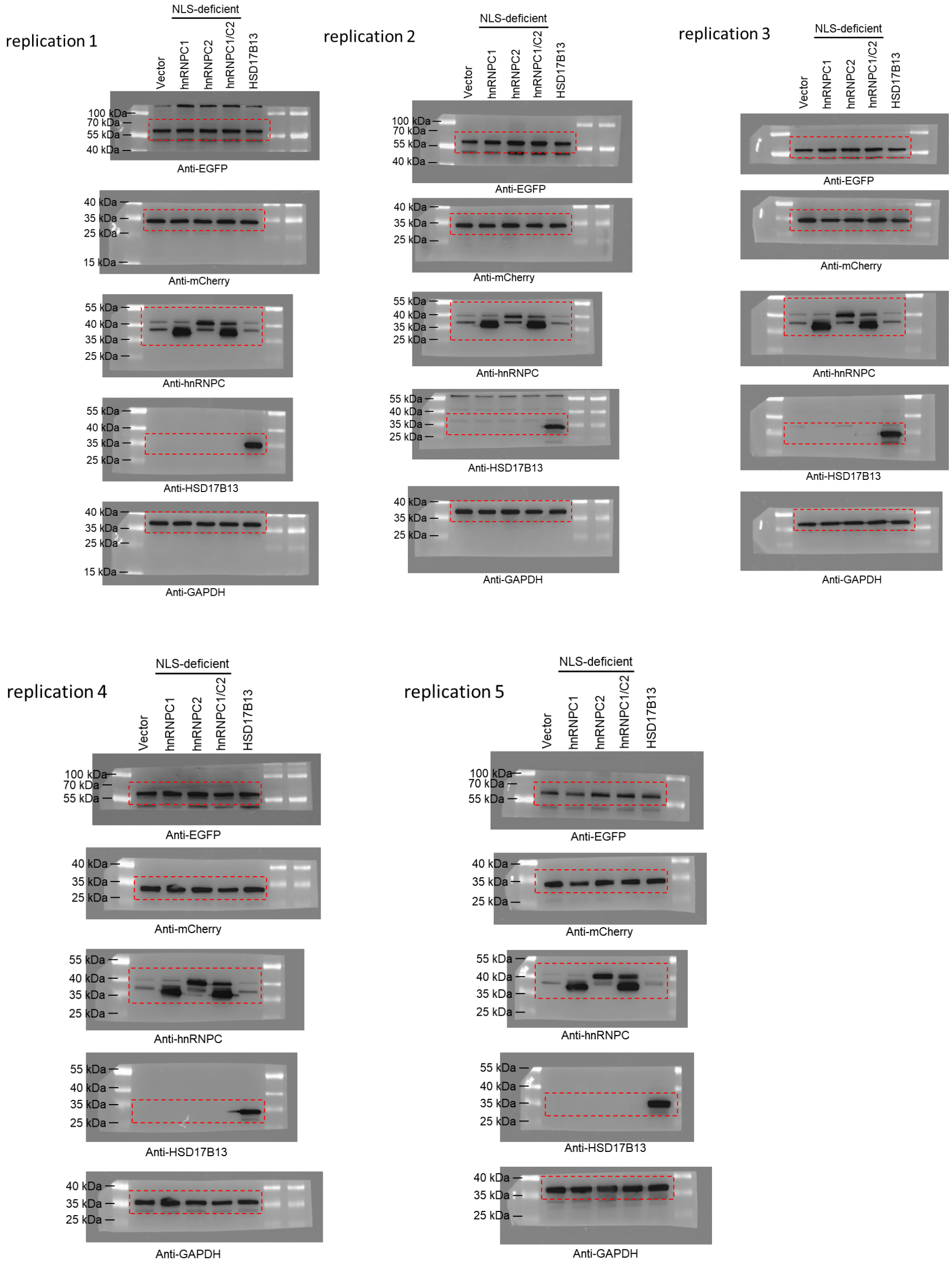

replication 1
replication 2
replication 3
replication 4
replication 5
NLS-deficient
Vector
hnRNPC1
hnRNPC2
hnRNPC1/C2
HSD17B13
100 kDa
70 kDa
55 kDa
40 kDa
35 kDa
25 kDa
15 kDa
Anti-EGFP
Anti-mCherry
Anti-hnRNPC
Anti-HSD17B13
Anti-GAPDH

Fig 6E

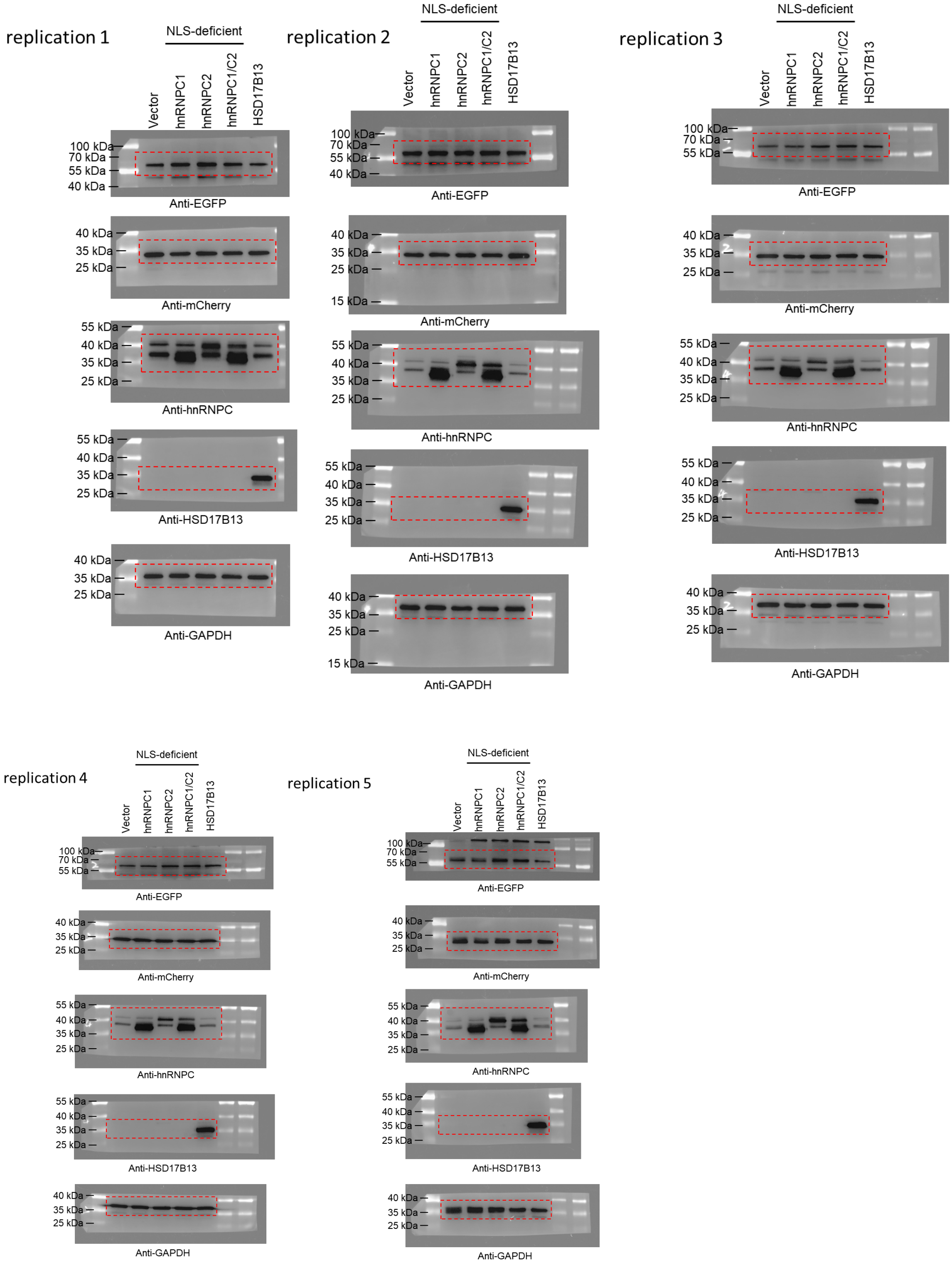

replication 1
NLS-deficient
Vector
hnRNPC1
hnRNPC2
hnRNPC1/C2
HSD17B13
100 kDa
70 kDa
55 kDa
40 kDa
Anti-EGFP
40 kDa
35 kDa
25 kDa
Anti-mCherry
55 kDa
40 kDa
35 kDa
25 kDa
Anti-hnRNPC
55 kDa
40 kDa
35 kDa
25 kDa
Anti-HSD17B13
40 kDa
35 kDa
25 kDa
Anti-GAPDH
replication 2
NLS-deficient
Vector
hnRNPC1
hnRNPC2
hnRNPC1/C2
HSD17B13
100 kDa
70 kDa
55 kDa
40 kDa
Anti-EGFP
40 kDa
35 kDa
25 kDa
15 kDa
Anti-mCherry
55 kDa
40 kDa
35 kDa
25 kDa
Anti-hnRNPC
55 kDa
40 kDa
35 kDa
25 kDa
Anti-HSD17B13
40 kDa
35 kDa
25 kDa
15 kDa
Anti-GAPDH
replication 3
NLS-deficient
Vector
hnRNPC1
hnRNPC2
hnRNPC1/C2
HSD17B13
100 kDa
70 kDa
55 kDa
Anti-EGFP
40 kDa
35 kDa
25 kDa
Anti-mCherry
55 kDa
40 kDa
35 kDa
25 kDa
Anti-hnRNPC
55 kDa
40 kDa
35 kDa
25 kDa
Anti-HSD17B13
40 kDa
35 kDa
25 kDa
Anti-GAPDH
replication 4
NLS-deficient
Vector
hnRNPC1
hnRNPC2
hnRNPC1/C2
HSD17B13
100 kDa
70 kDa
55 kDa
Anti-EGFP
40 kDa
35 kDa
25 kDa
Anti-mCherry
55 kDa
40 kDa
35 kDa
25 kDa
Anti-hnRNPC
55 kDa
40 kDa
35 kDa
25 kDa
Anti-HSD17B13
40 kDa
35 kDa
25 kDa
Anti-GAPDH
replication 5
NLS-deficient
Vector
hnRNPC1
hnRNPC2
hnRNPC1/C2
HSD17B13
100 kDa
70 kDa
55 kDa
Anti-EGFP
40 kDa
35 kDa
25 kDa
Anti-mCherry
55 kDa
40 kDa
35 kDa
25 kDa
Anti-hnRNPC
55 kDa
40 kDa
35 kDa
25 kDa
Anti-HSD17B13
40 kDa
35 kDa
25 kDa
Anti-GAPDH

Fig 6F

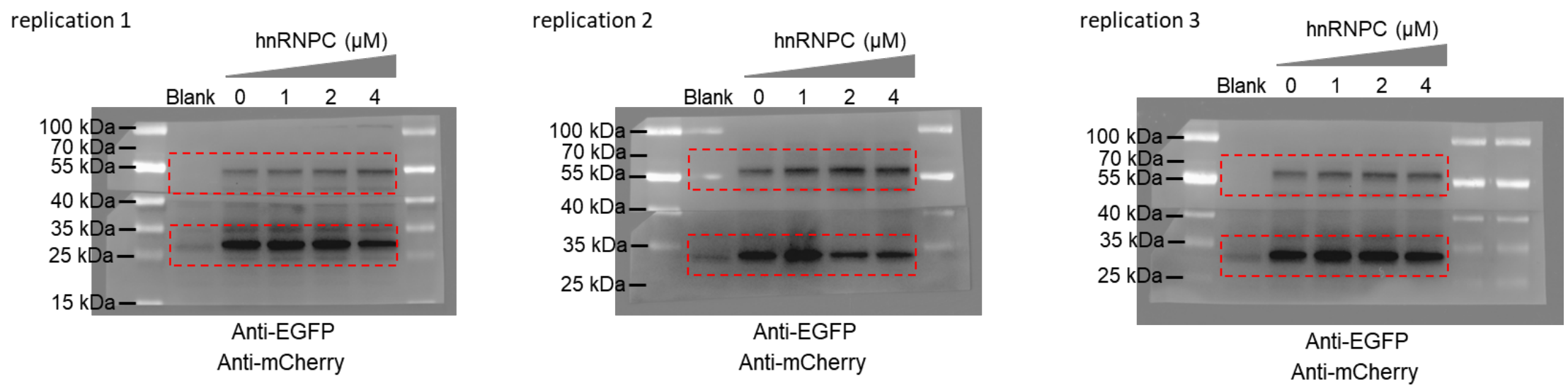

replication 1
hnRNPC (μM)
Blank 0 1 2 4
100 kDa
70 kDa
55 kDa
40 kDa
35 kDa
25 kDa
15 kDa
Anti-EGFP
Anti-mCherry
replication 2
hnRNPC (μM)
Blank 0 1 2 4
100 kDa
70 kDa
55 kDa
40 kDa
35 kDa
25 kDa
Anti-EGFP
Anti-mCherry
replication 3
hnRNPC (μM)
Blank 0 1 2 4
100 kDa
70 kDa
55 kDa
40 kDa
35 kDa
25 kDa
Anti-EGFP
Anti-mCherry

Fig 7B

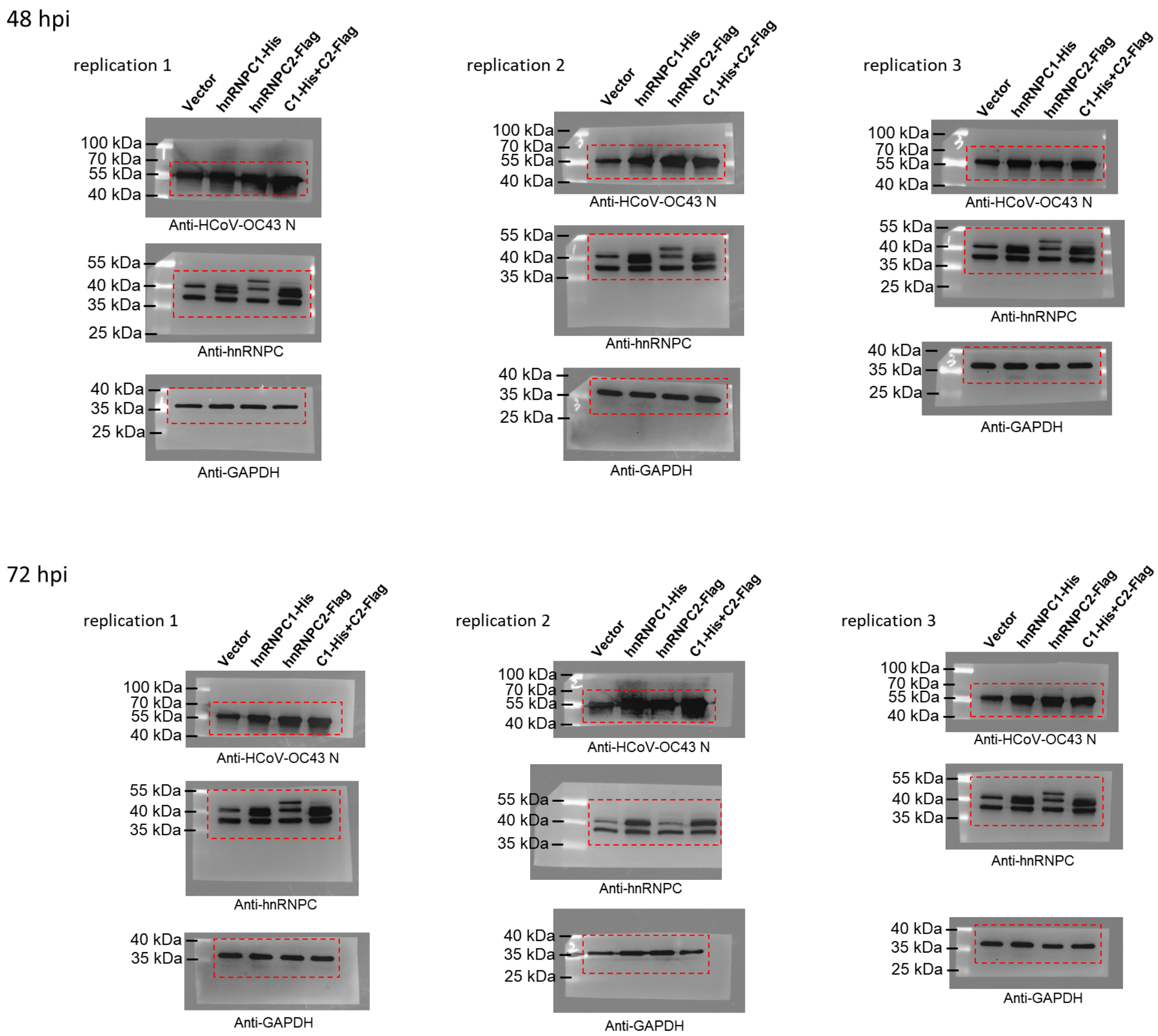

48 hpi
replication 1
Vector
hnRNPC1-His
hnRNPC2-Flag
C1-His+C2-Flag
100 kDa
70 kDa
55 kDa
40 kDa
Anti-HCoV-OC43 N
55 kDa
40 kDa
35 kDa
25 kDa
Anti-hnRNPC
40 kDa
35 kDa
25 kDa
Anti-GAPDH
replication 2
Vector
hnRNPC1-His
hnRNPC2-Flag
C1-His+C2-Flag
100 kDa
70 kDa
55 kDa
40 kDa
Anti-HCoV-OC43 N
55 kDa
40 kDa
35 kDa
Anti-hnRNPC
40 kDa
35 kDa
25 kDa
Anti-GAPDH
replication 3
Vector
hnRNPC1-His
hnRNPC2-Flag
C1-His+C2-Flag
100 kDa
70 kDa
55 kDa
40 kDa
Anti-HCoV-OC43 N
55 kDa
40 kDa
35 kDa
25 kDa
Anti-hnRNPC
40 kDa
35 kDa
25 kDa
Anti-GAPDH
72 hpi
replication 1
Vector
hnRNPC1-His
hnRNPC2-Flag
C1-His+C2-Flag
100 kDa
70 kDa
55 kDa
40 kDa
Anti-HCoV-OC43 N
55 kDa
40 kDa
35 kDa
Anti-hnRNPC
40 kDa
35 kDa
Anti-GAPDH
replication 2
Vector
hnRNPC1-His
hnRNPC2-Flag
C1-His+C2-Flag
100 kDa
70 kDa
55 kDa
40 kDa
Anti-HCoV-OC43 N
55 kDa
40 kDa
35 kDa
Anti-hnRNPC
40 kDa
35 kDa
25 kDa
Anti-GAPDH
replication 3
Vector
hnRNPC1-His
hnRNPC2-Flag
C1-His+C2-Flag
100 kDa
70 kDa
55 kDa
40 kDa
Anti-HCoV-OC43 N
55 kDa
40 kDa
35 kDa
Anti-hnRNPC
40 kDa
35 kDa
25 kDa
Anti-GAPDH

Fig 7C

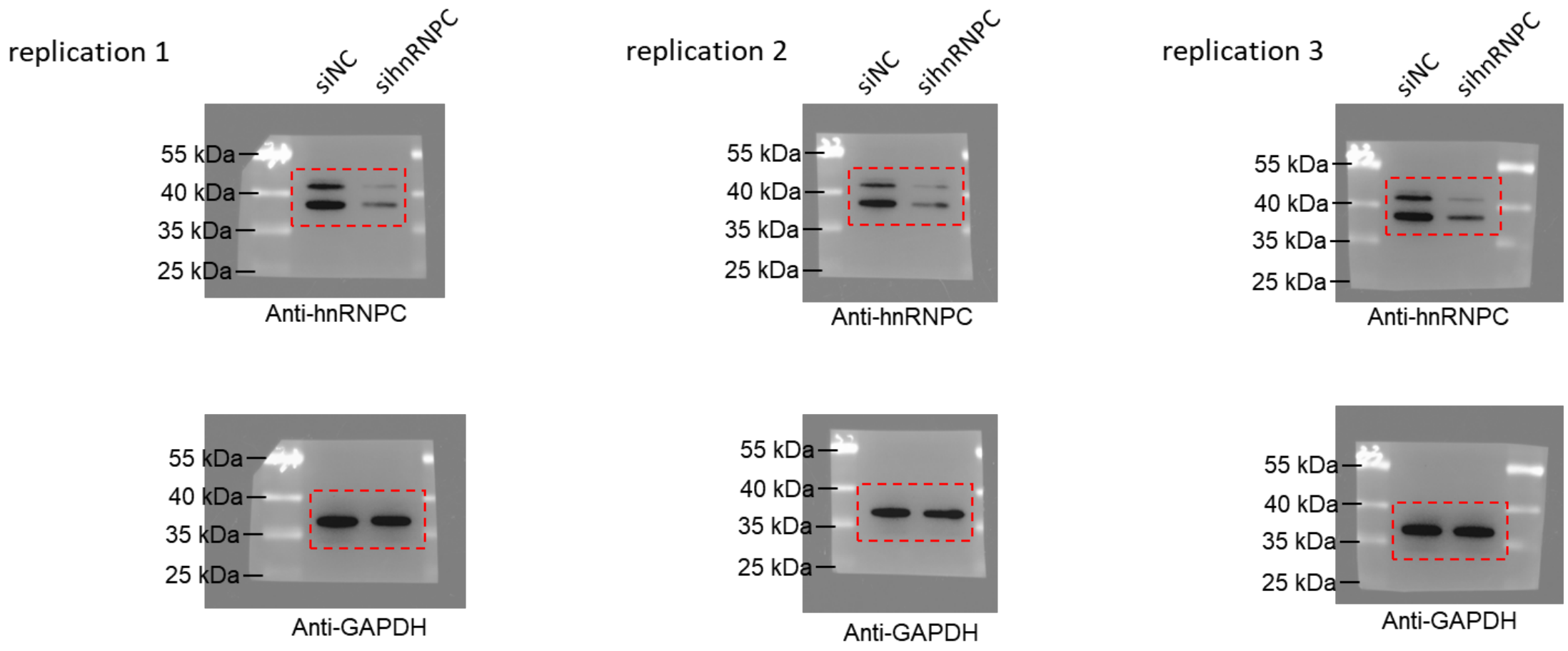

Fig 7E

24 hpi

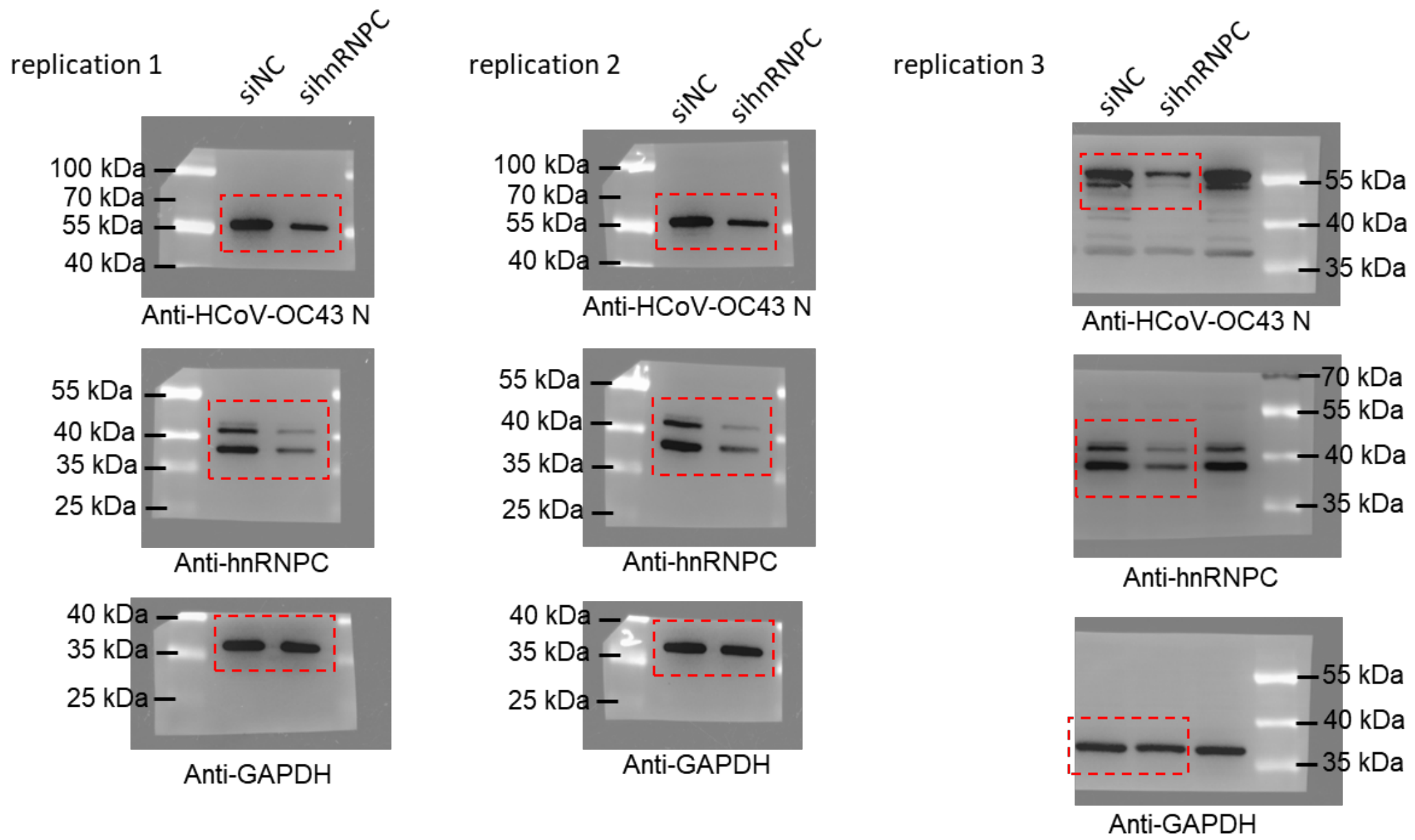

48 hpi

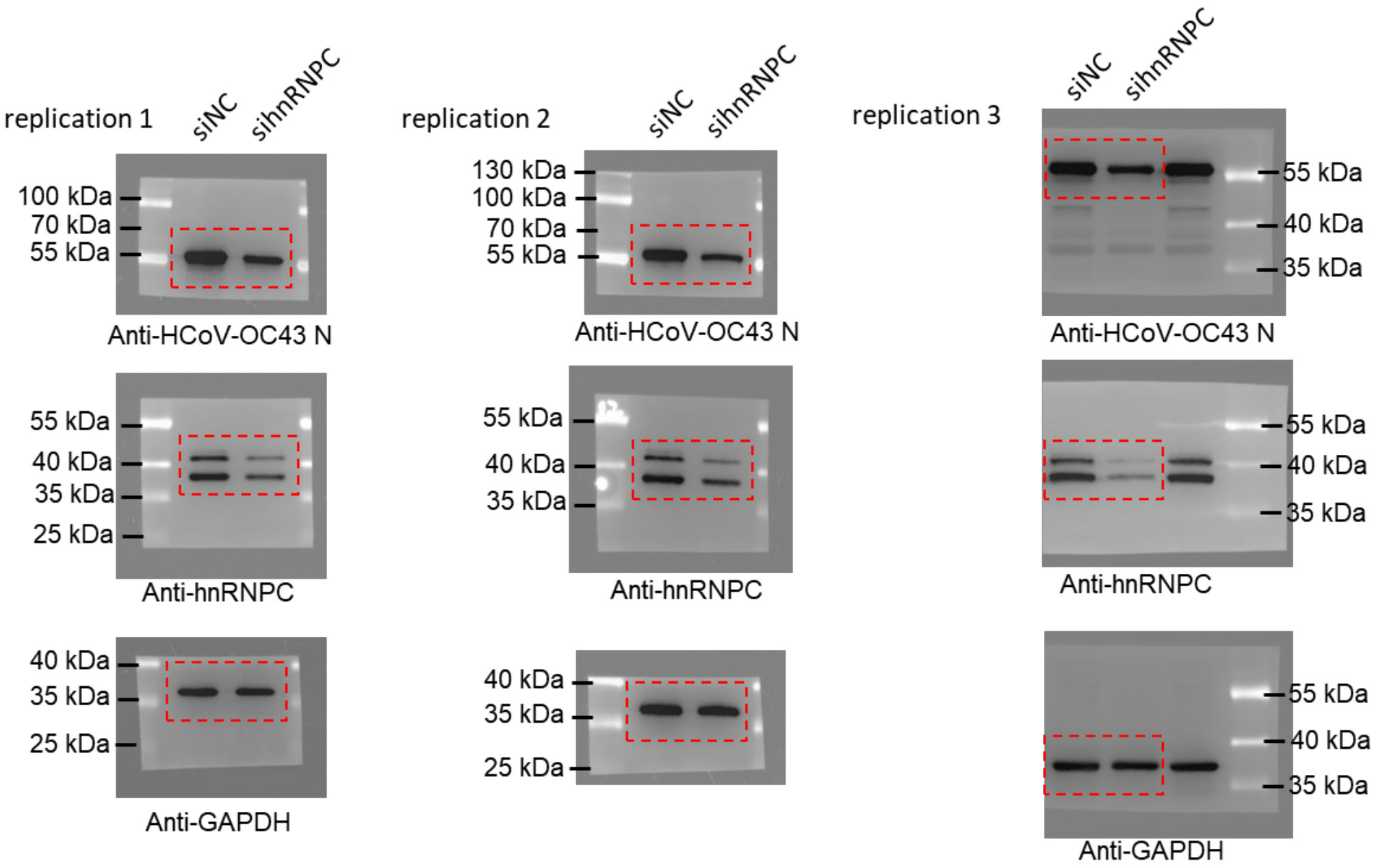
replication 1
siNC
sihnRNPC
100 kDa
70 kDa
55 kDa
Anti-HCoV-OC43 N
55 kDa
40 kDa
35 kDa
25 kDa
Anti-hnRNPC
40 kDa
35 kDa
25 kDa
Anti-GAPDH
replication 2
siNC
sihnRNPC
130 kDa
100 kDa
70 kDa
55 kDa
Anti-HCoV-OC43 N
55 kDa
40 kDa
35 kDa
Anti-hnRNPC
40 kDa
35 kDa
25 kDa
replication 3
siNC
sihnRNPC
55 kDa
40 kDa
35 kDa
Anti-HCoV-OC43 N
55 kDa
40 kDa
35 kDa
Anti-hnRNPC
55 kDa
40 kDa
35 kDa
Anti-GAPDH

72 hpi

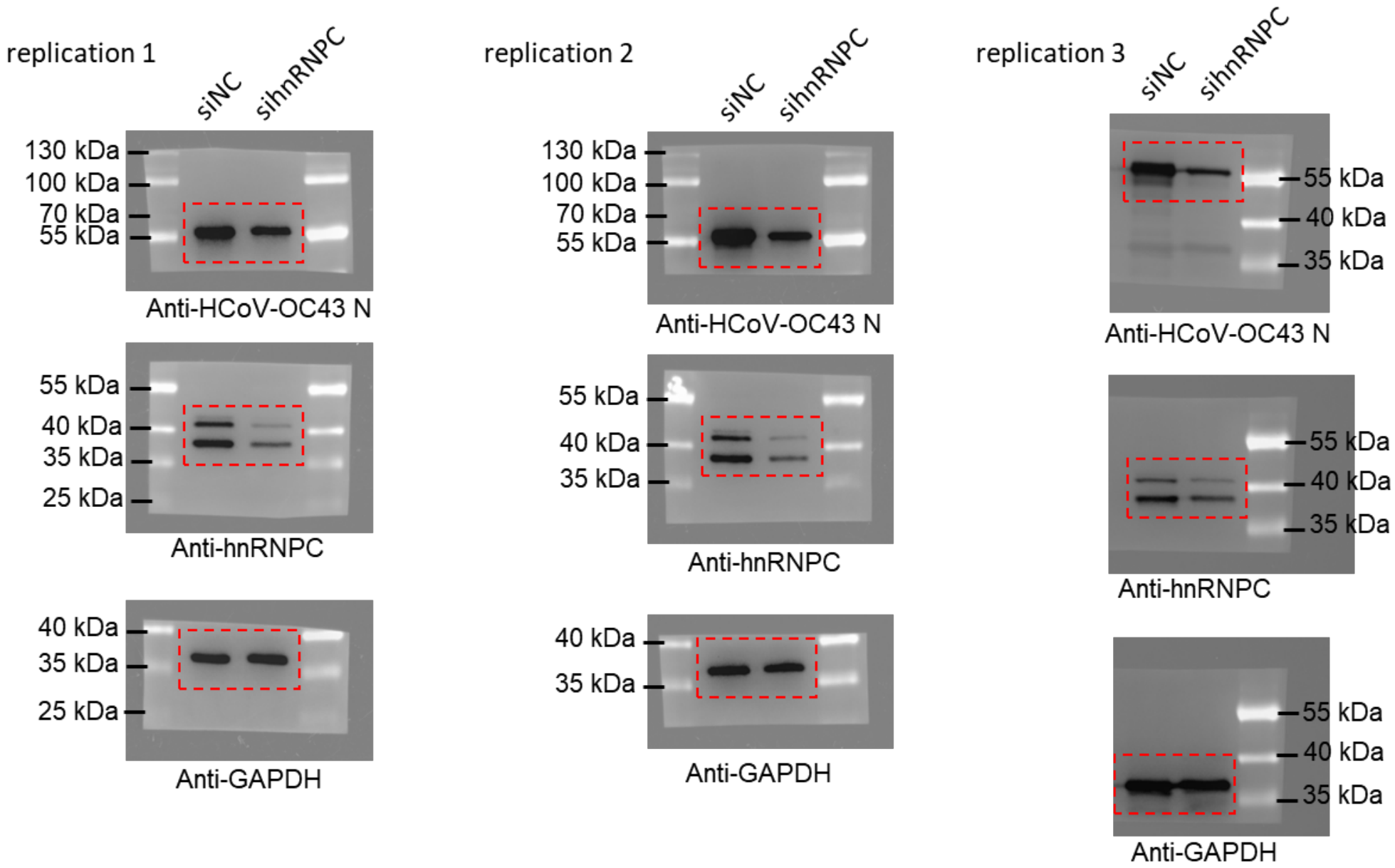
replication 1
siNC
sihnRNPC
130 kDa
100 kDa
70 kDa
55 kDa
Anti-HCoV-OC43 N
55 kDa
40 kDa
35 kDa
25 kDa
Anti-hnRNPC
40 kDa
35 kDa
25 kDa
Anti-GAPDH
replication 2
siNC
sihnRNPC
130 kDa
100 kDa
70 kDa
55 kDa
Anti-HCoV-OC43 N
55 kDa
40 kDa
35 kDa
Anti-hnRNPC
40 kDa
35 kDa
Anti-GAPDH
replication 3
siNC
sihnRNPC
55 kDa
40 kDa
35 kDa
Anti-HCoV-OC43 N
55 kDa
40 kDa
35 kDa
Anti-hnRNPC
55 kDa
40 kDa
35 kDa
Anti-GAPDH

Fig 8F

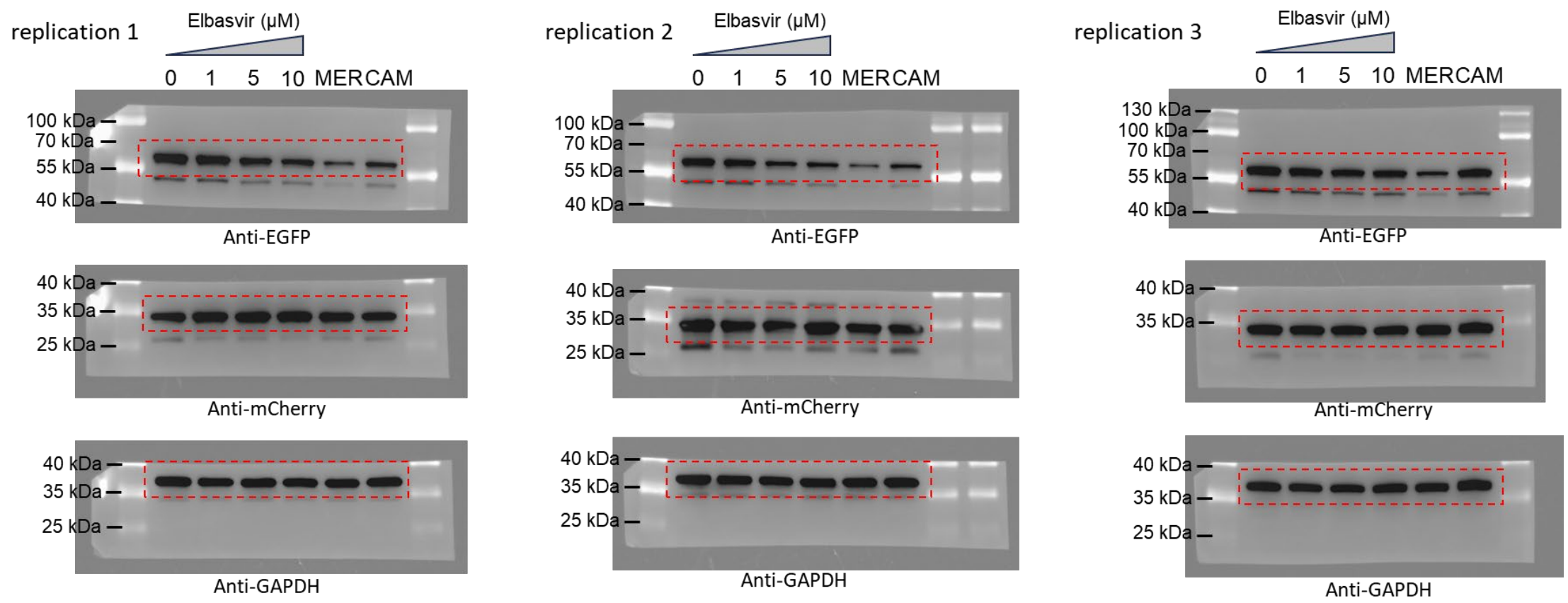

Fig 8G

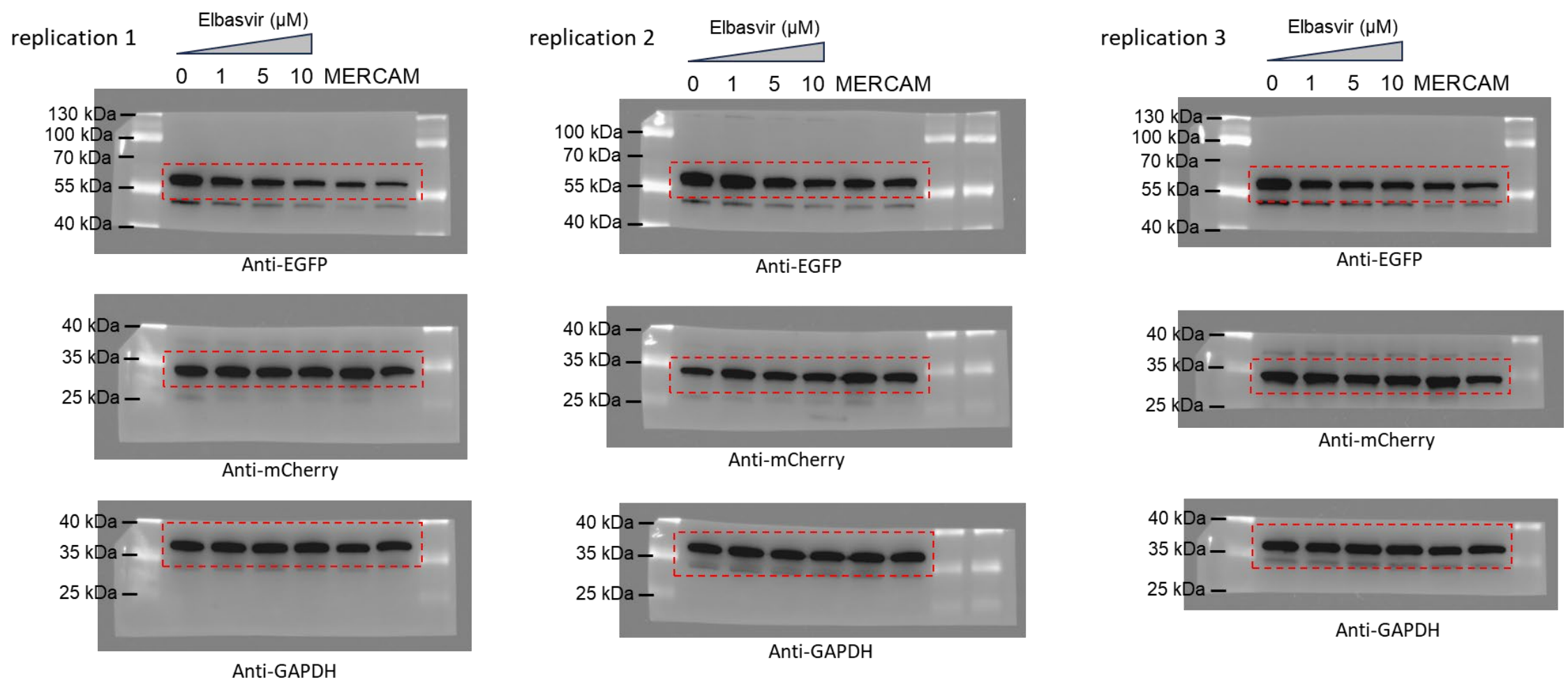

Fig 8H

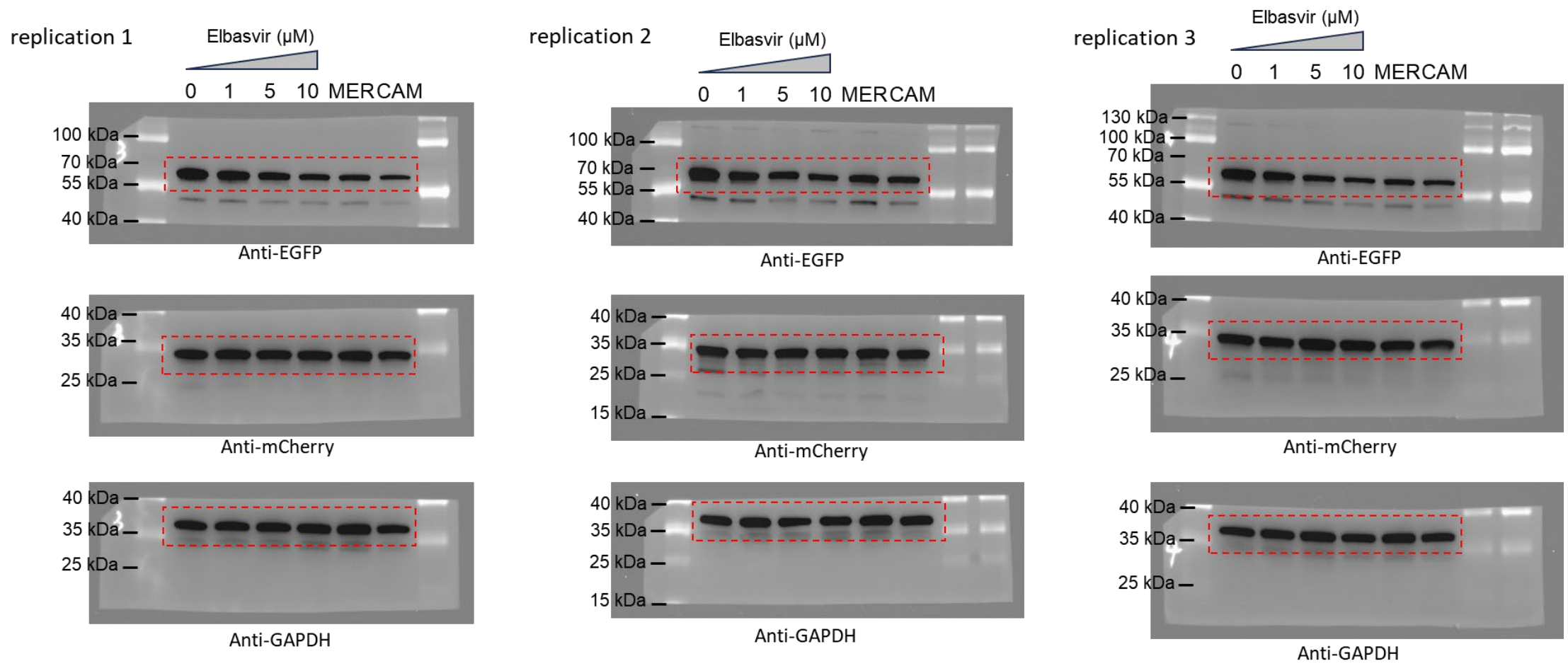

S3A Fig

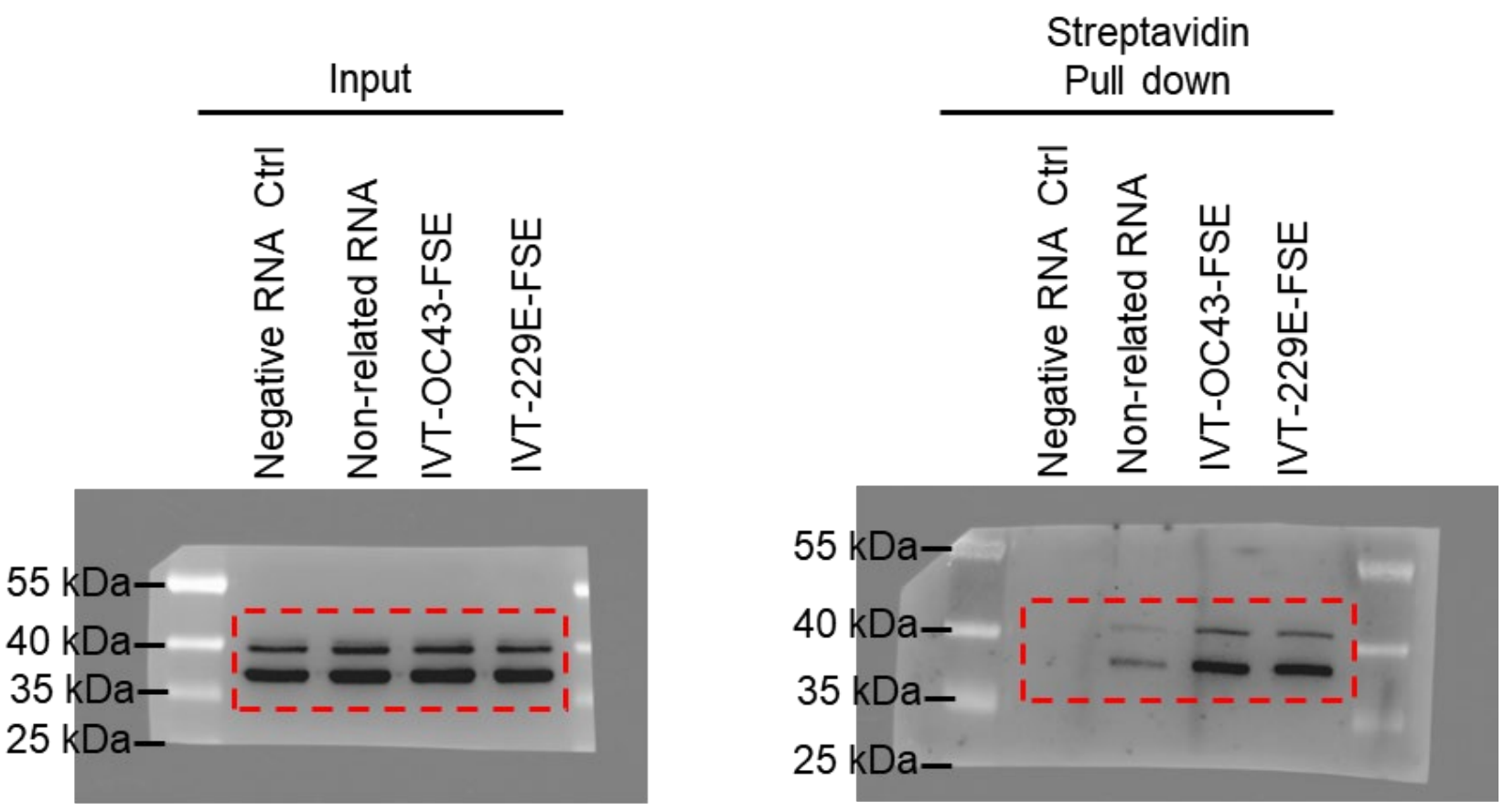

S3B Fig

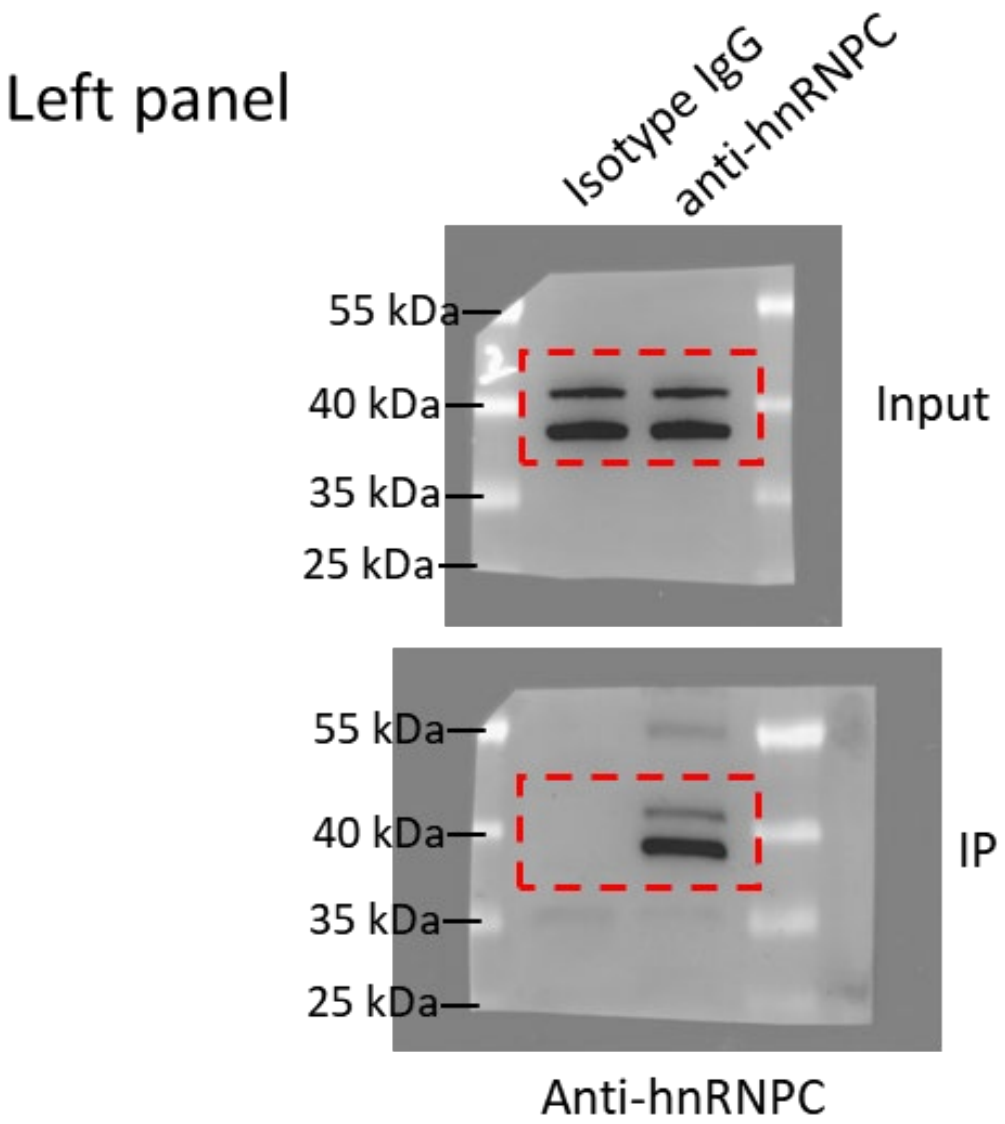

S3C Fig

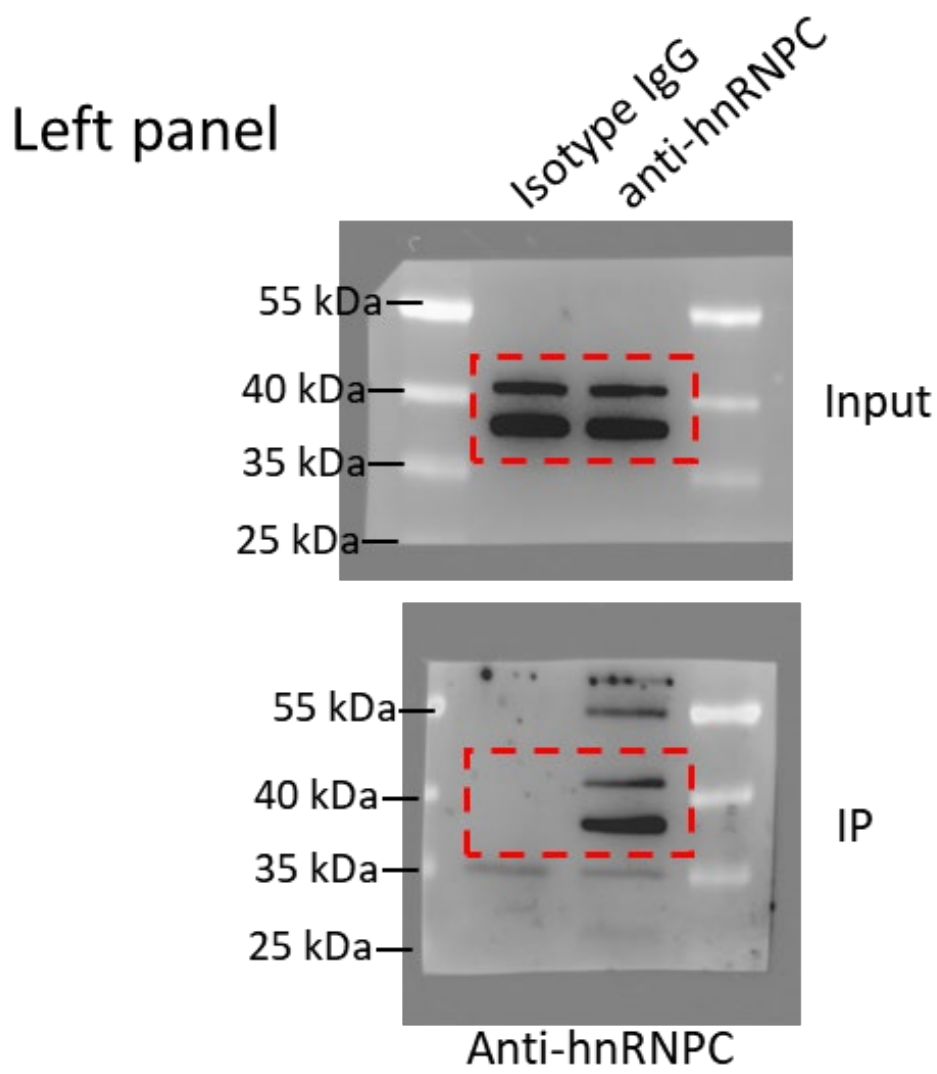

S4 Fig

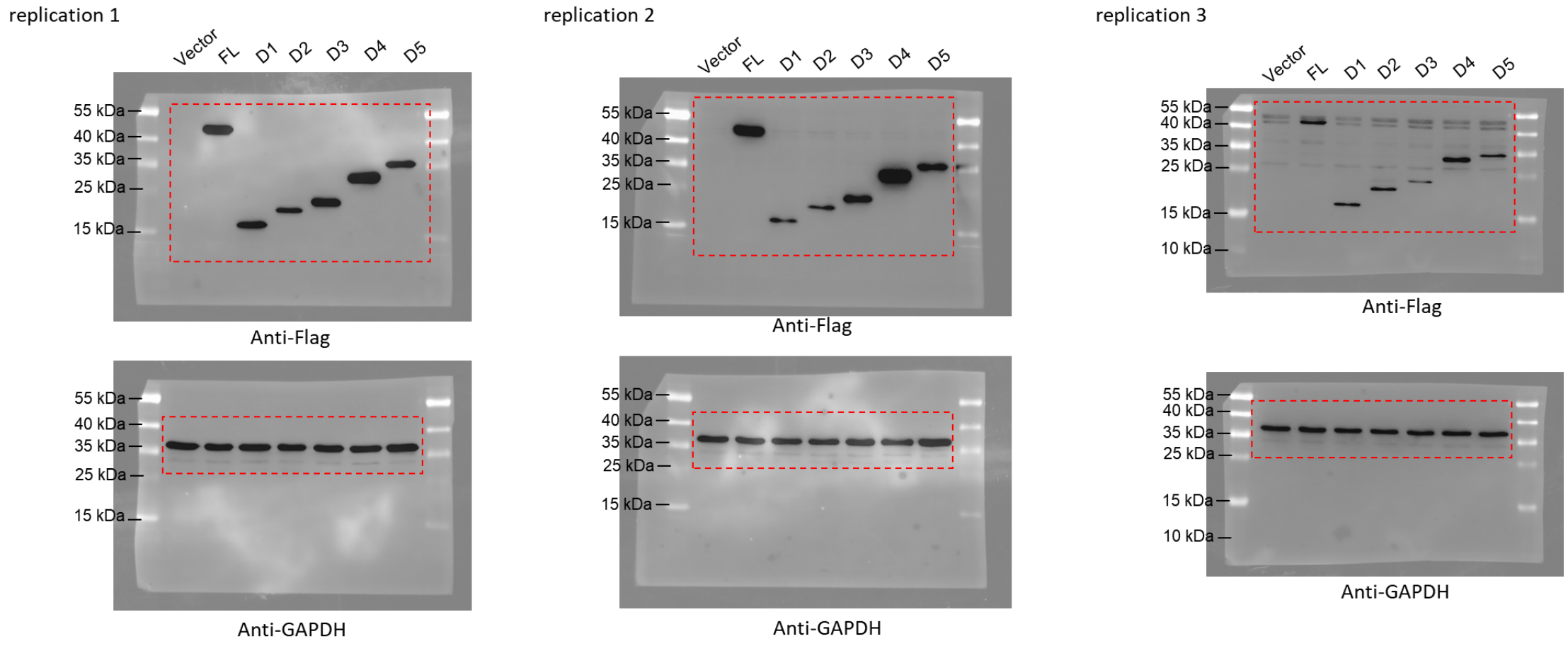

S6B Fig

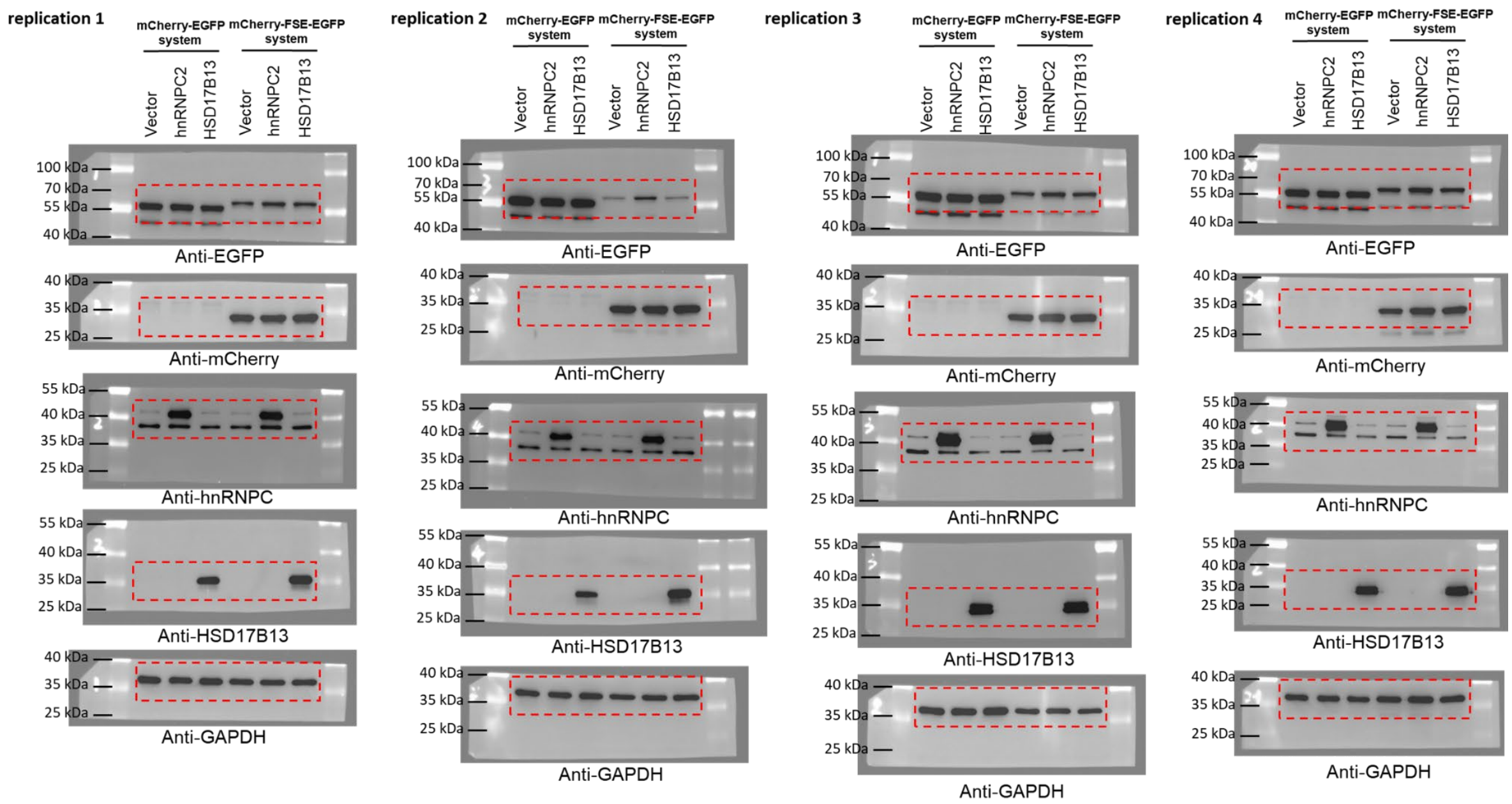

S7 Fig

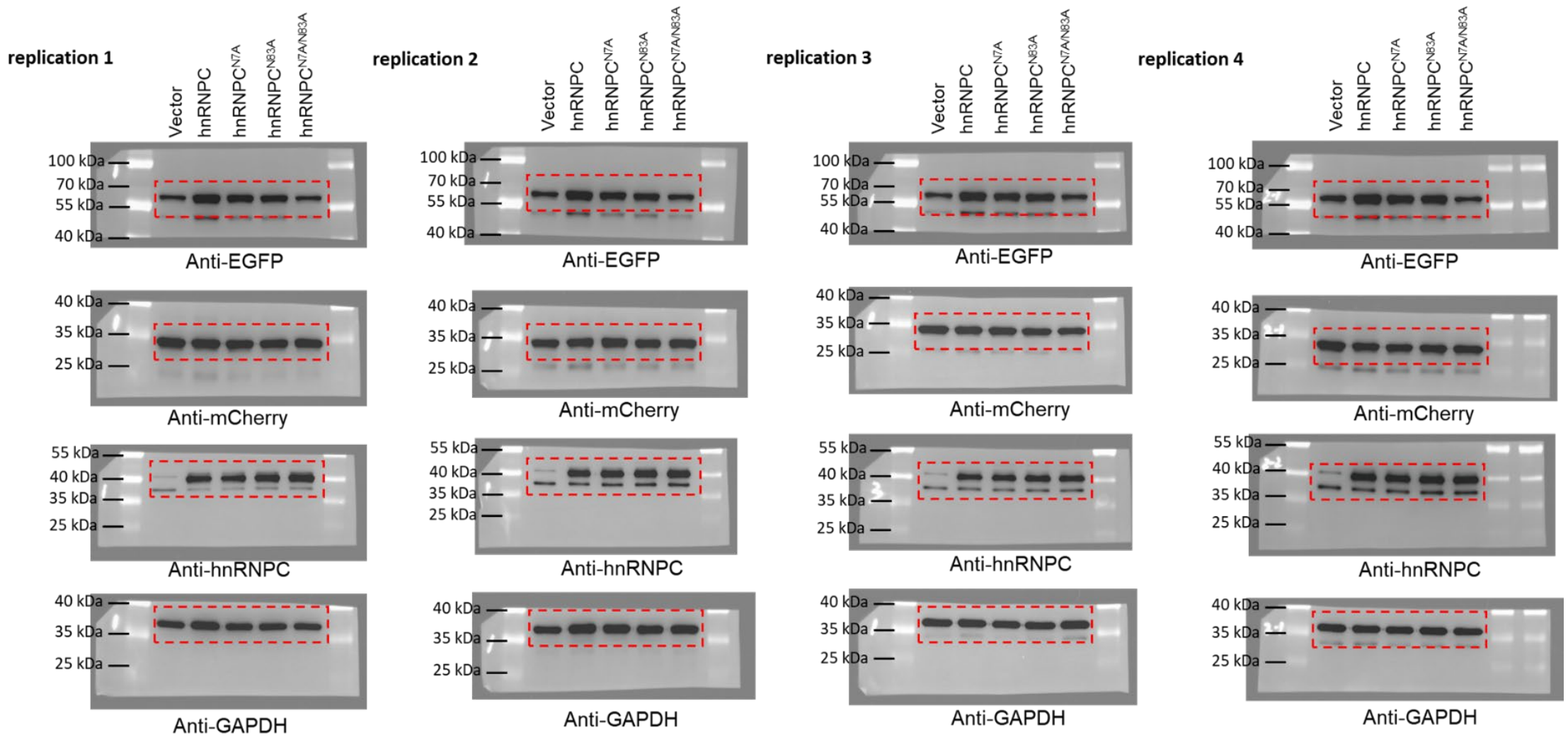

S10B Fig

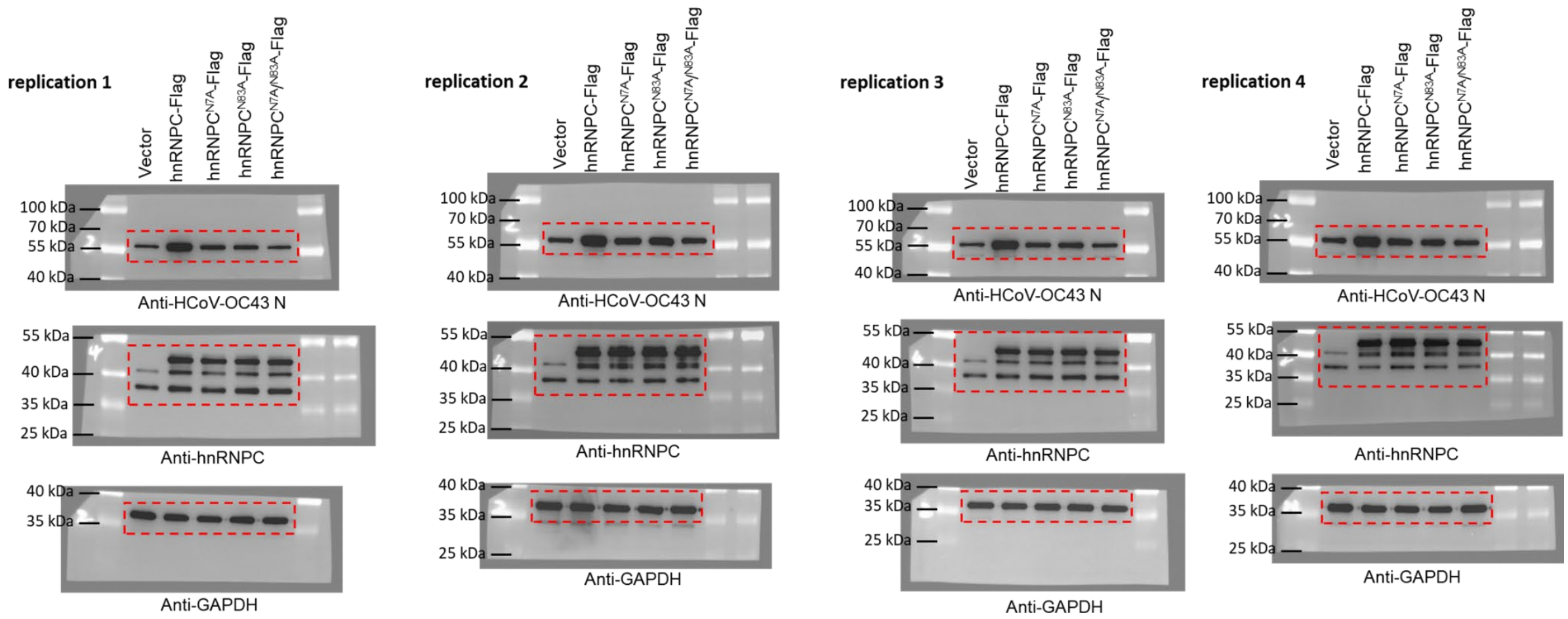

S11B Fig

48 hpi

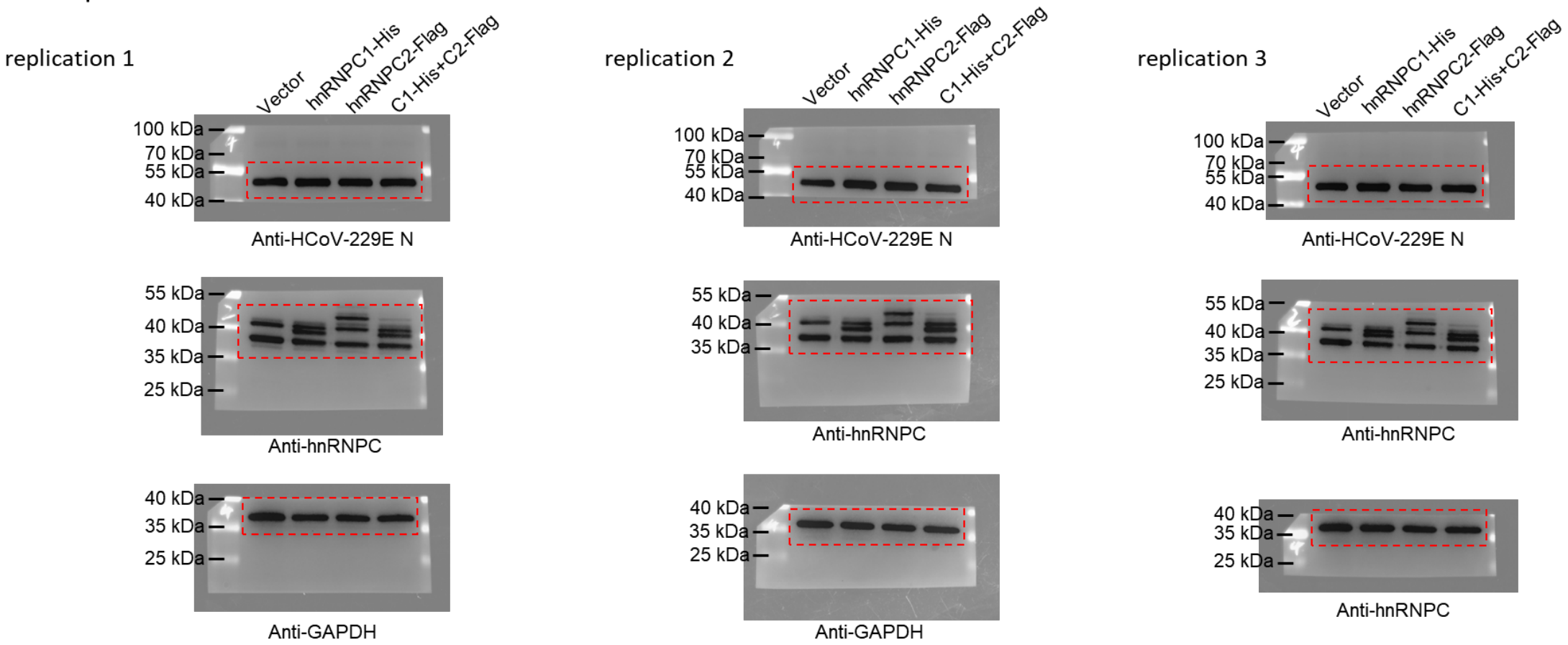

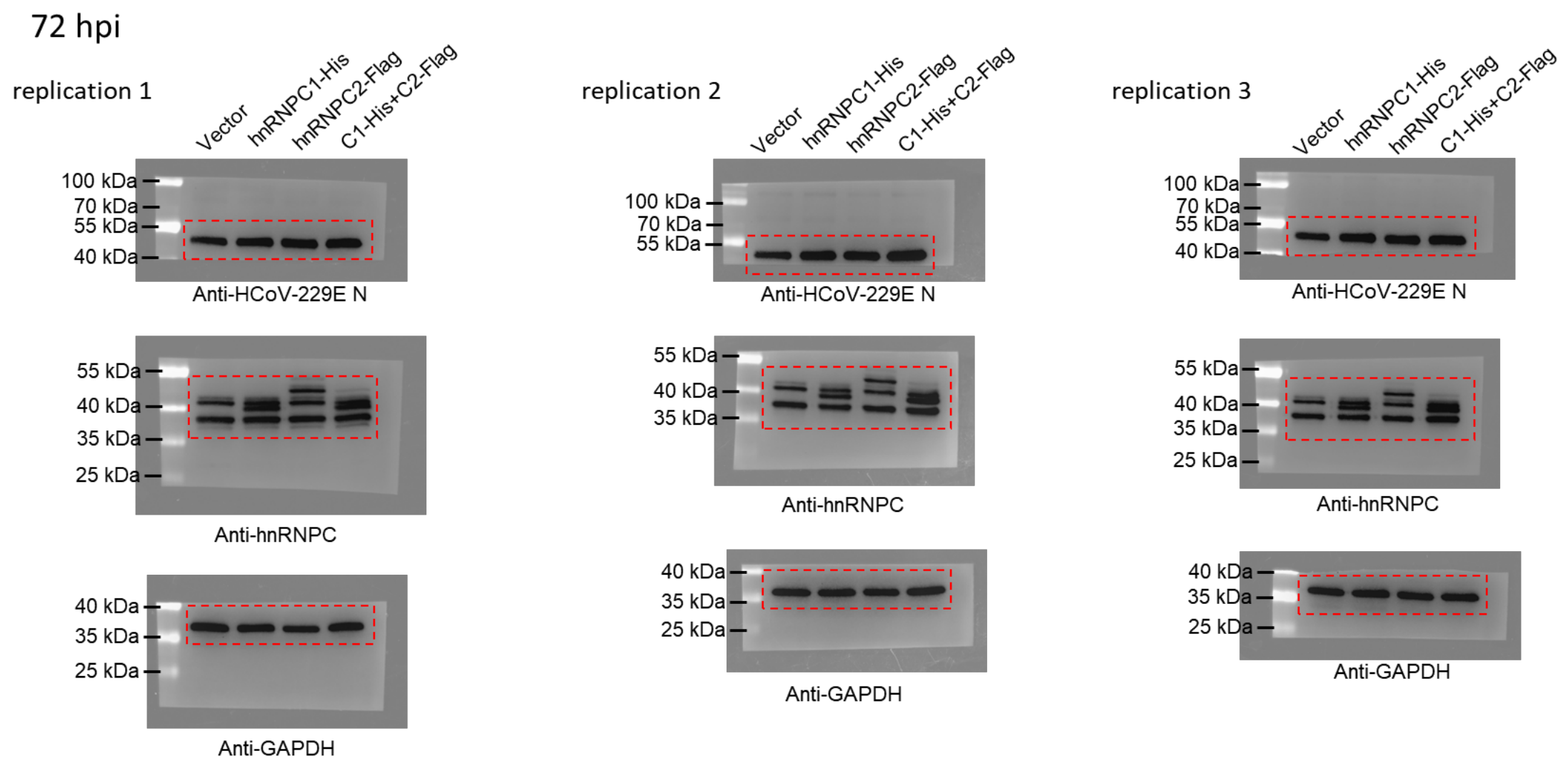

S11C Fig

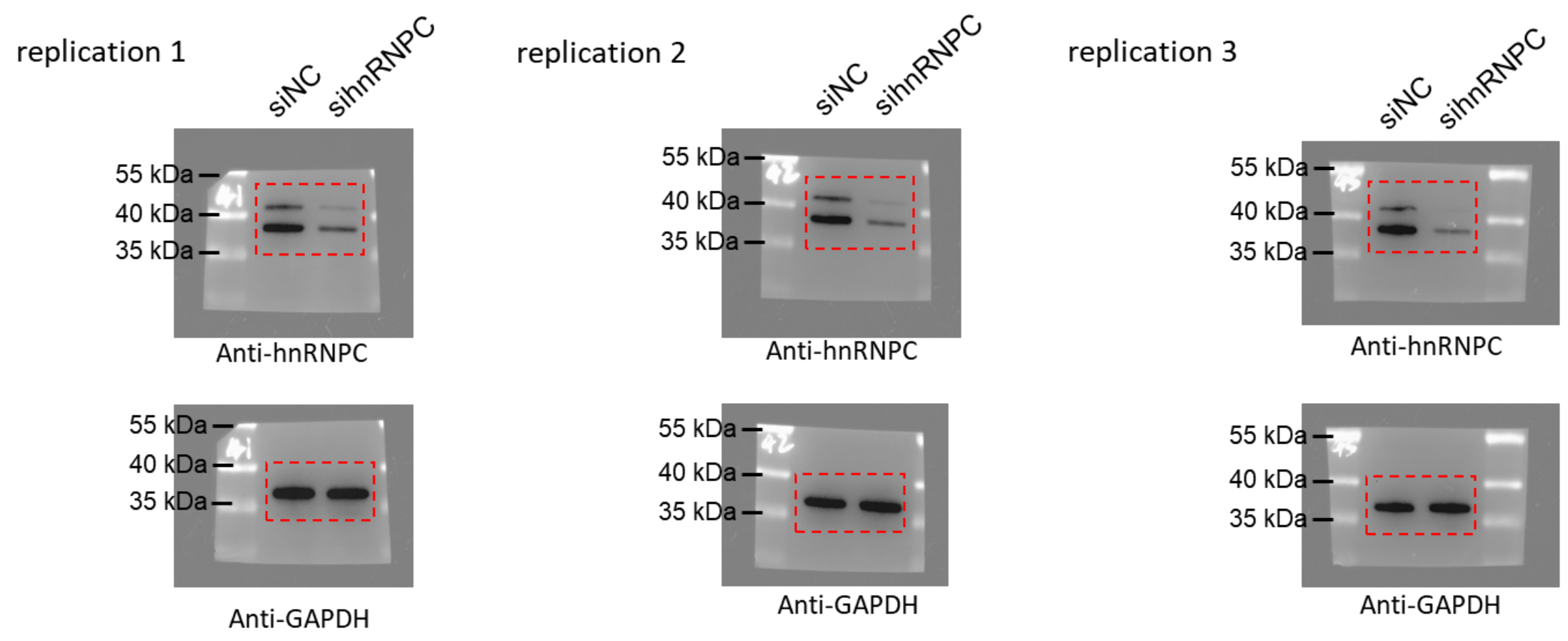

S11E Fig

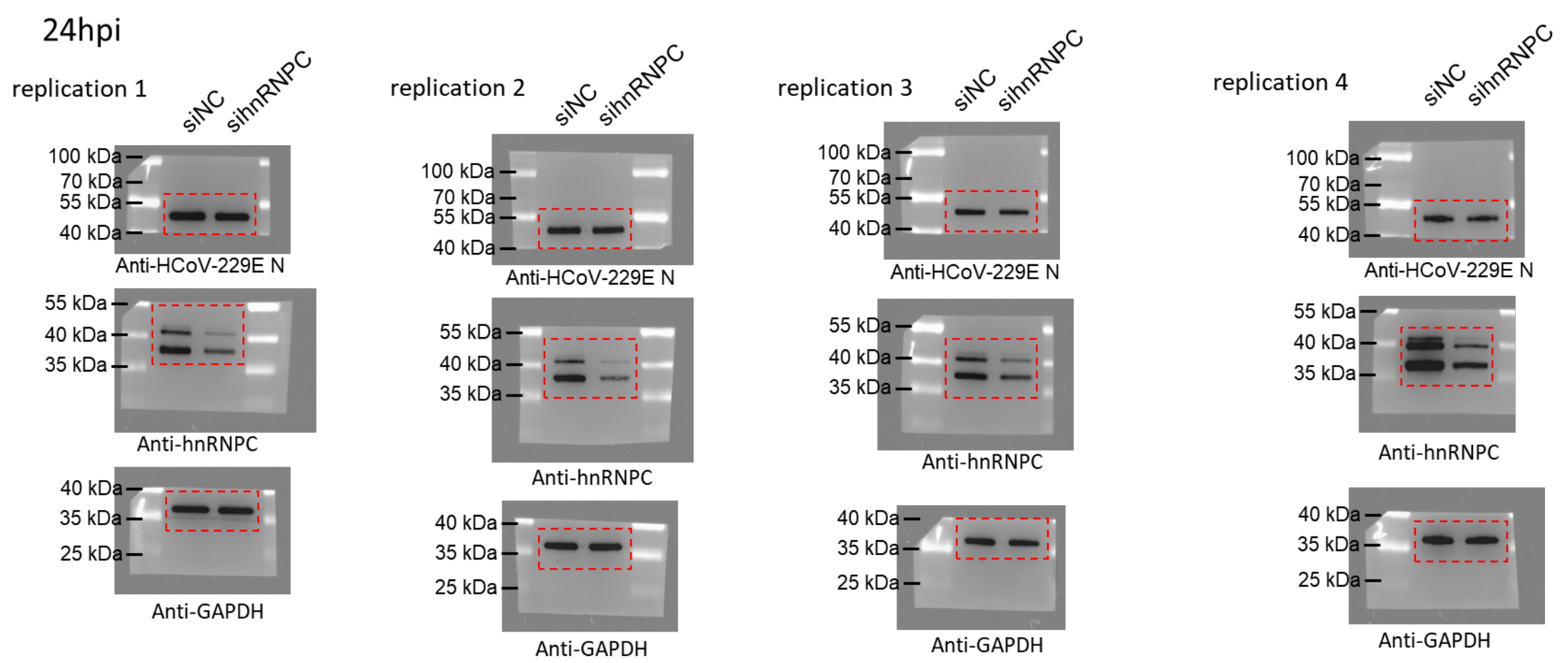

48 hpi

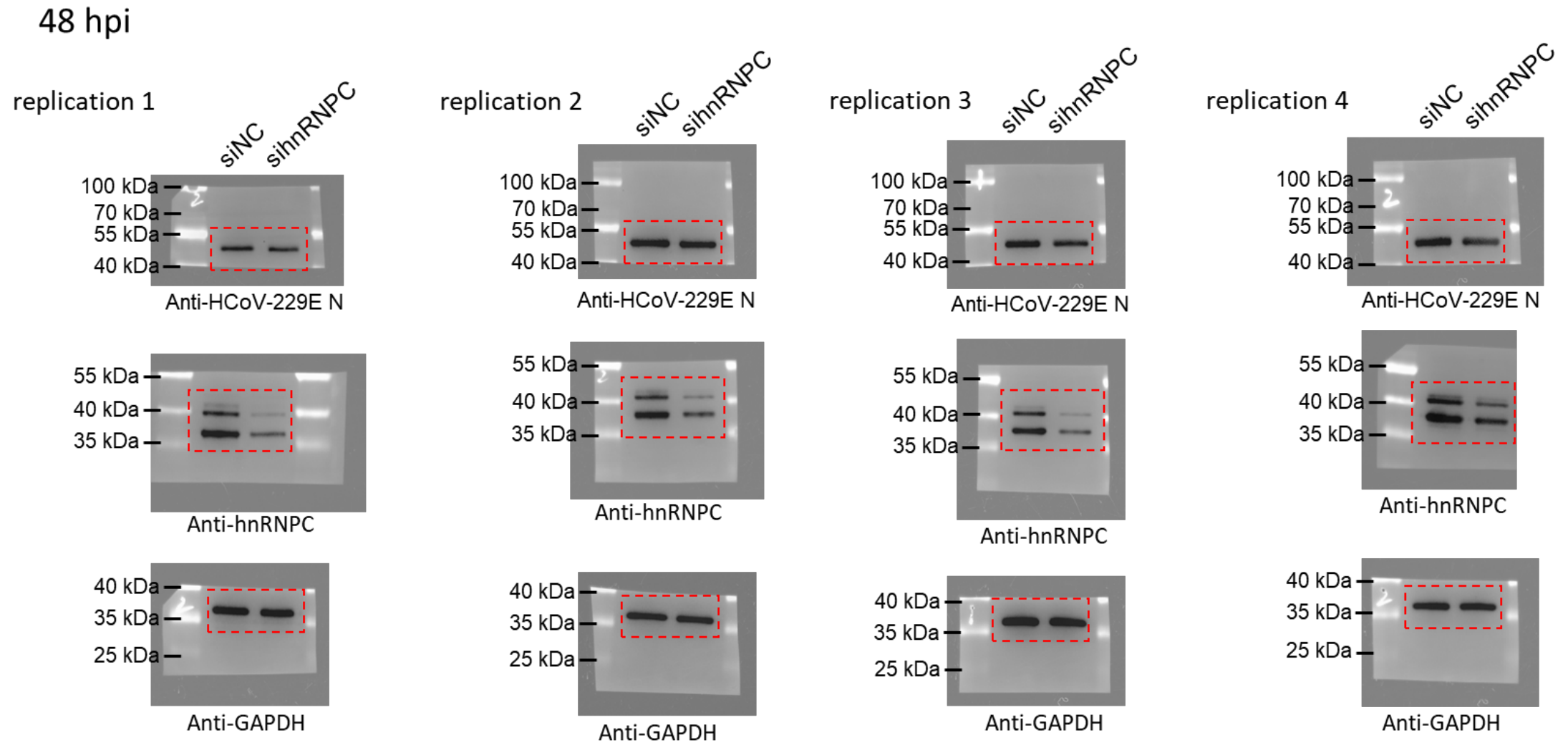

72 hpi

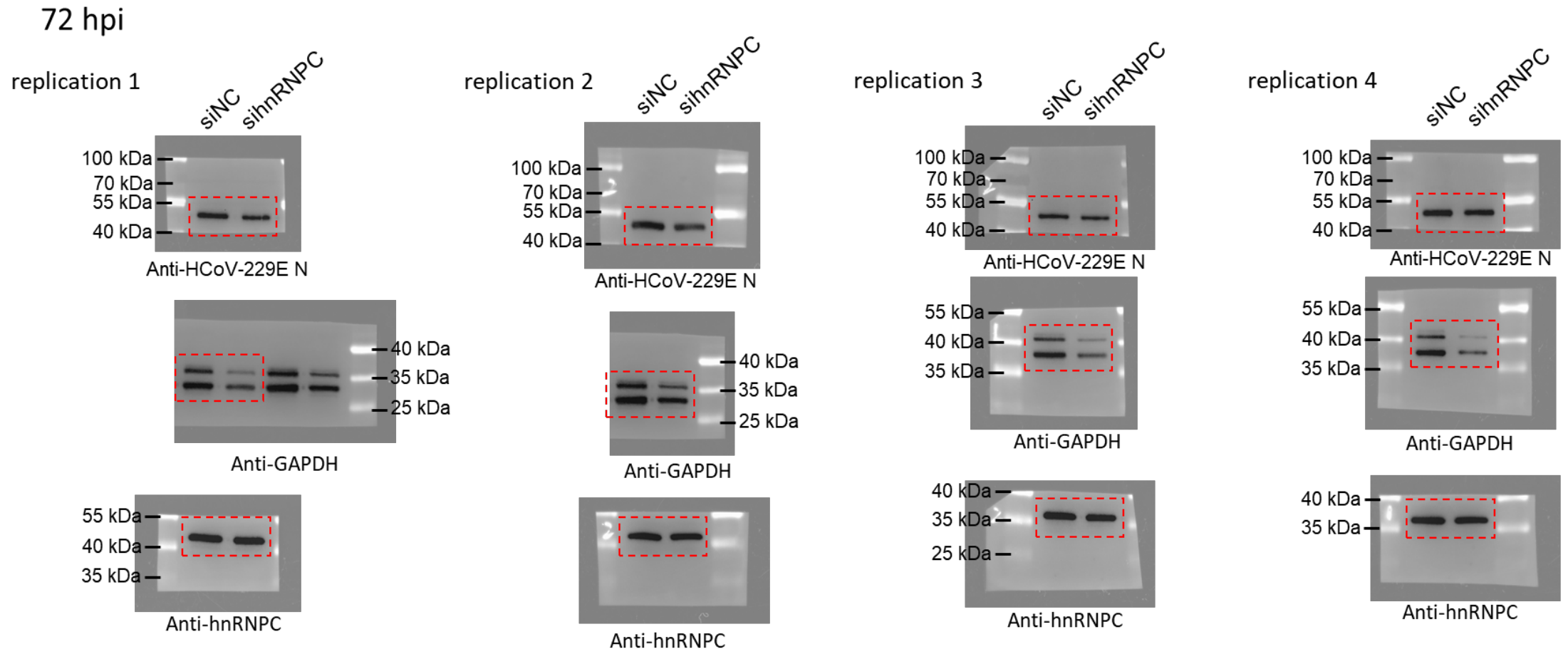

S13 Fig

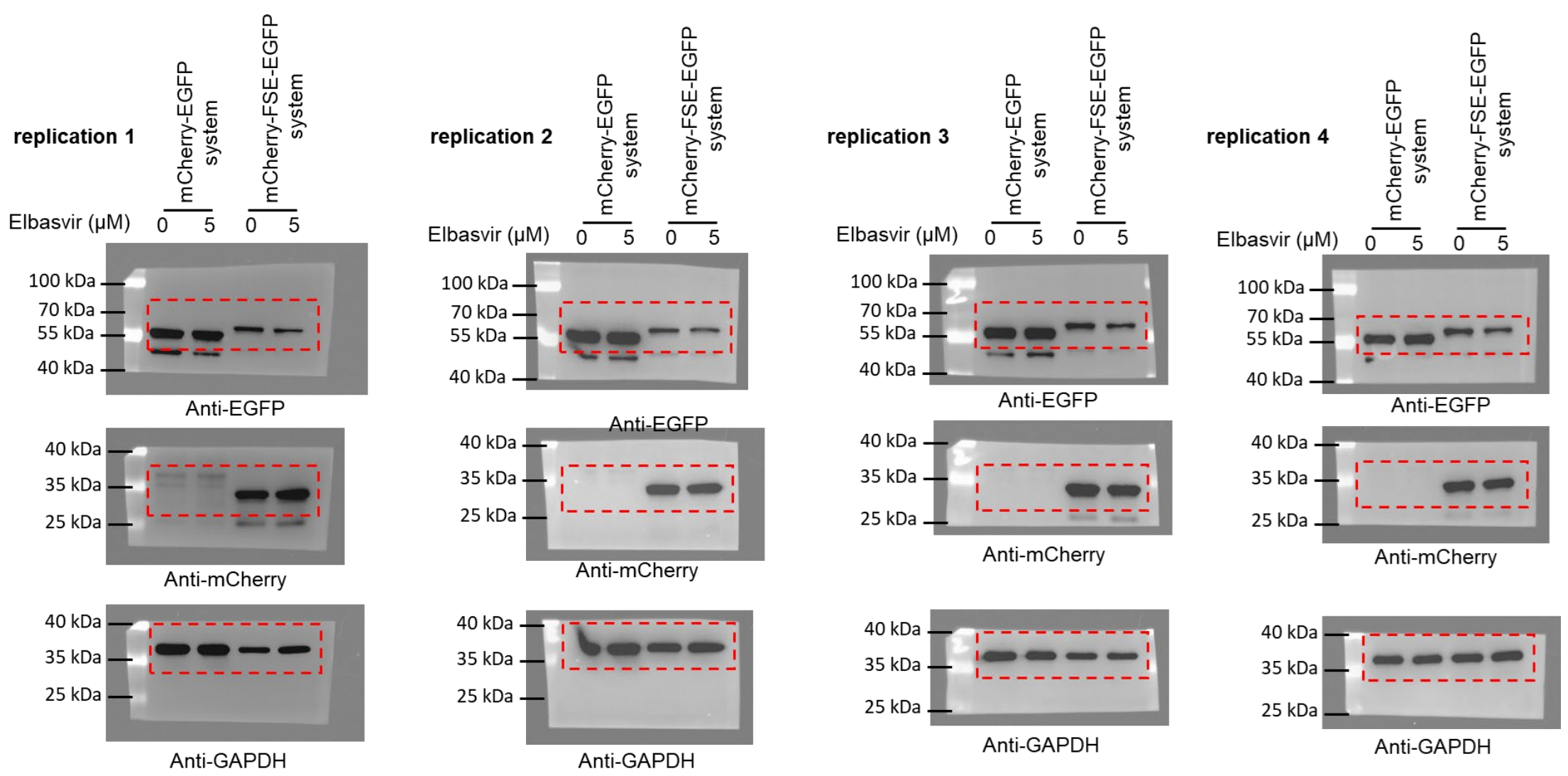

S14 Fig

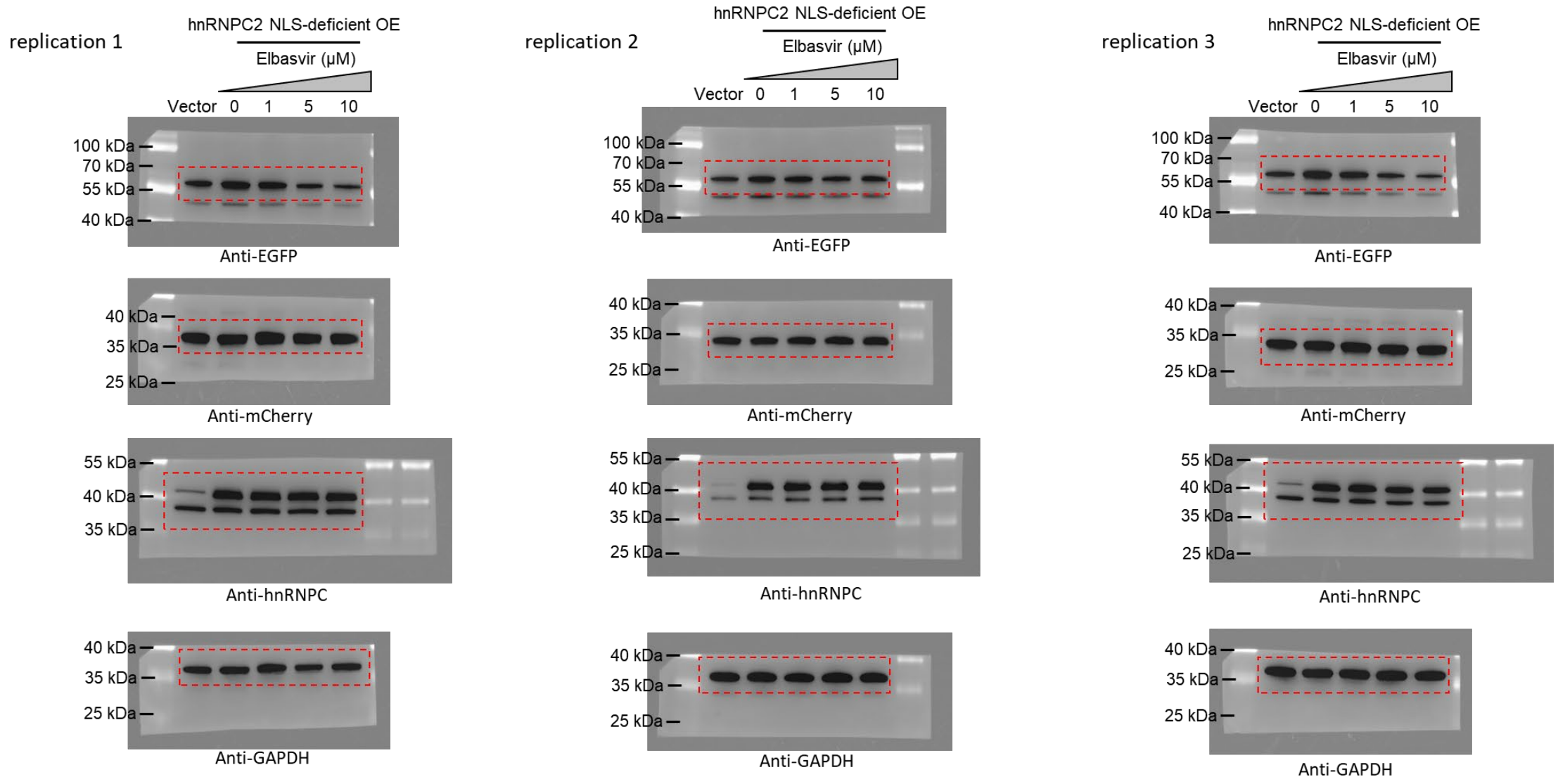
replication 1
hnRNPC2 NLS-deficient OE
Elbasvir (μM)
Vector
0
1
5
10
100 kDa
70 kDa
55 kDa
40 kDa
Anti-EGFP
40 kDa
35 kDa
25 kDa
Anti-mCherry
55 kDa
40 kDa
35 kDa
Anti-hnRNPC
40 kDa
35 kDa
25 kDa
Anti-GAPDH
replication 2
hnRNPC2 NLS-deficient OE
Elbasvir (μM)
Vector
0
1
5
10
100 kDa
70 kDa
55 kDa
40 kDa
Anti-EGFP
40 kDa
35 kDa
25 kDa
Anti-mCherry
55 kDa
40 kDa
35 kDa
25 kDa
Anti-hnRNPC
40 kDa
35 kDa
25 kDa
Anti-GAPDH
replication 3
hnRNPC2 NLS-deficient OE
Elbasvir (μM)
Vector
0
1
5
10
100 kDa
70 kDa
55 kDa
40 kDa
Anti-EGFP
40 kDa
35 kDa
25 kDa
Anti-mCherry
55 kDa
40 kDa
35 kDa
25 kDa
Anti-hnRNPC
40 kDa
35 kDa
25 kDa
Anti-GAPDH
