# Supplementary material for: hnRNPC facilitates coronavirus replication by directly binding the frameshift-stimulatory element of viral genomic RNA
Source: PLoS Pathog. 2026 Aug 7;22(8):e1014491. doi: 10.1371/journal.ppat.1014491 (PMC13450726; doi:10.1371/journal.ppat.1014491)
Supplement: Supplementary file 2 [file ppat.1014491.s017.docx]

**S1 Table.** **Data information of host factors captured by SARS-CoV-2 FSE RNA.**

| Gene symbol | Sum PEP score | | Abundance | |
| --- | --- | --- | --- | --- |
|  | HCoV-OC43 | HCoV-229E | HCoV-OC43 | HCoV-229E |
| HNRNPC | 7.438 | 3.172 | 338847488 | 13932218 |
| GRSF1 | 3.063 | 1.800 | 131544192 | 2272203 |
| MTHFD1L | 2.543 | 6.082 | 25628510 | 11971726 |
| SLC25A18 | 4.824 | 3.838 | 10302548 | - |
| TMEM245 | 1.273 | 1.470 | 65806296 | 14840648 |
