# Supplementary material for: hnRNPC facilitates coronavirus replication by directly binding the frameshift-stimulatory element of viral genomic RNA
Source: PLoS Pathog. 2026 Aug 7;22(8):e1014491. doi: 10.1371/journal.ppat.1014491 (PMC13450726; doi:10.1371/journal.ppat.1014491)
Supplement: Supplementary file 3 [file ppat.1014491.s018.docx]

**S2 Table.** **Primers for qRT-PCR.**

| Gene name | Forward primer (5′-3′) | Reverse primer (5′-3′) |
| --- | --- | --- |
| HCoV-OC43 N | TACGGCACCGATATTGACGG | GTGCGCGAAGTAGATCTGGA |
| HCoV-OC43 RdRp | AGCGTAATGTCCTGCCAACA | AGGAACACCACGTGTAGCTG |
| HCoV-229E N | TGTCGTCTGGGTTGCTGTTGATG | AAGGAGCACGGGAGTCAGGTTC |
| HCoV-229E RdRp | TAAACGAGTCCGGGGCTCTA | CGTCAAATGCACGGACACAG |
| hnRNPC | CTGGCACCCTTTCTGAGGTT | AGCTGTCGCCATTTTCCTGA |
| SARS-CoV-2 FSE | AACGGGTTTGCGGTGTAAGT | AAGCCCTGTATACGACATCAGT |
| HCoV-OC43 FSE | CCTTTTTAAACGGGTTCGGGG | CAGTAGATAAACCATTGGCGCA |
| HCoV-229E FSE | TAAACGAGTCCGGGGCTCTA | CGTCAAATGCACGGACACAG |
| SARS-CoV-2 FSE  ΔSlippery | GGTTTGCGGTGTAAGTGCAG | AGCCCTGTATACGACATCAGT |
| SARS-CoV-2 FSE  ΔSL1 | CCCAAGCTGGCTAGCGTTTA | CAGTACTAGTGCCTGTGCCG |
| SARS-CoV-2 FSE  ΔSL2 | ATAGGGAGACCCAAGCTGGC | CTGCACTTACACCGCAAACC |
| SARS-CoV-2 FSE  ΔSL3 | CCCAAGCTGGCTAGCGTTTA | CCTGTACGGTGTAAGACGGG |
| Human GAPDH | CGGAGTCAACGGATTTGGTCGTAT | AGCCTTCTCCATGGTGGTGAAGAC |
