# Supplementary material for: hnRNPC facilitates coronavirus replication by directly binding the frameshift-stimulatory element of viral genomic RNA
Source: PLoS Pathog. 2026 Aug 7;22(8):e1014491. doi: 10.1371/journal.ppat.1014491 (PMC13450726; doi:10.1371/journal.ppat.1014491)
Supplement: Supplementary file 4 [file ppat.1014491.s019.docx]

**S3 Table.** **Sequences of siRNAs.**

| Gene name | Sense (5′-3′) | Antisense (5′-3′) |
| --- | --- | --- |
| NC | GCGCUUGUCUAAGAUCAAAUU | AAUUUGAUCUUAGACAAGCGC |
| hnRNPC | UUCUCCGAACGUGUCACGU | ACGUGACACGUUCGGAGAA |
