# Supplementary material for: hnRNPC facilitates coronavirus replication by directly binding the frameshift-stimulatory element of viral genomic RNA
Source: PLoS Pathog. 2026 Aug 7;22(8):e1014491. doi: 10.1371/journal.ppat.1014491 (PMC13450726; doi:10.1371/journal.ppat.1014491)
Supplement: Supplementary file 5 [file ppat.1014491.s020.docx]

**S4 Table. RNA sequences.**

| **Name** | **Sequence (5'–3')** |
| --- | --- |
| Positive RNA Control  (U-rich single-stranded RNA) | CUGGGCUUUUUUUUUCUCUUUCUCUCCUUUCUUUUUCUUCUUCCCUCCCUA |
| Negative RNA Control | poly(A)_25_ |
| Non-related RNA | CCUGGUUUUUAAGGAGUGUCGCCAGAGUGCCGCGAAUGAAAAA |
| SARS-CoV-2 FSE | UUUUUAAACGGGUUUGCGGUGUAAGUGCAGCCCGUCUUACACCGUGCGGCACAGGCACUAGUACUGAUGUCGUAUACAGGGCUUUUGAC |
| HCoV-OC43 FSE | UUUUUAAACGGGUUCGGGGUGCGAGUGUAGAUGCCCGUCUCGUACCCUGCGCCAAUGGUUUAUCUACUGAUGUACAAUUAAGGGCAUUUGAUGAAUUC |
| HCoV-229E FSE | UAUUUAAACGAGUCCGGGGCUCUAGUGCCGCUCGACUAGAGCCCUGUAAUGGUACAGACAUAGAUUACUGUGUCCGUGCAUUUGAC |
| SARS-CoV-2 FSE  ΔSlippery | UUGGGUUUGCGGUGUAAGUGCAGCCCGUCUUACACCGUGCGGCACAGGCACUAGUACUGAUGUCGUAUACAGGGCUUUUGAC |
| SARS-CoV-2 FSE  ΔSL1 | UUUUUAAACGGGUUUGUGCGGCACAGGCACUAGUACUGAUGUCGUAUACAGGGCUUUUGAC |
| SARS-CoV-2 FSE  ΔSL2 | UUUUUAAACGGGUUUGCGGUGUAAGUGCAGCCCGUCUUACACCGUGCGGCACAGGCACUAGUACUGAUGUCGUA |
| SARS-CoV-2 FSE  ΔSL3 | UUUUUAAACGGGUUUGCGGUGUAAGUGCAGCCCGUCUUACACCGUACAGGGCUUUUGAC |
| SARS-CoV-2 FSE  U mut | CGGCCAAACGGGCGGGCGGCGGAAGGGCAGCCCGGCCGACACCGGGCGGCACAGGCACGAGGACGGACGGCGGAGACAGGGCCCCCGAC |
